# Study on the Microwave-Assisted Batch and Continuous Flow Synthesis of *N*-Alkyl-Isoindolin-1-One-3-Phosphonates by a Special Kabachnik–Fields Condensation

**DOI:** 10.3390/molecules25143307

**Published:** 2020-07-21

**Authors:** Ádám Tajti, Nóra Tóth, Bettina Rávai, István Csontos, Pál Tamás Szabó, Erika Bálint

**Affiliations:** 1 Department of Organic Chemistry and Technology, Budapest University of Technology and Economics, 1521 Budapest, Hungary; tajti.adam@mail.bme.hu (Á.T.); toth.nora@mail.bme.hu (N.T.); betti.ravai08@gmail.com (B.R.); icsontos@mail.bme.hu (I.C.); 2 MS Metabolomics Laboratory, Instrumentation Center, Research Centre for Natural Sciences, Hungarian Academy of Sciences, Magyar tudósok krt. 2., H-1117 Budapest, Hungary; szabo.pal@ttk.mta.hu

**Keywords:** isoindolin-1-one phosphonates, α-aminophosphonate derivatives, three-component condensation, Kabachnik–Fields reaction, microwave, in situ FT-IR spectroscopy, continuous flow microwave reactor

## Abstract

A simple and efficient microwave (MW)-assisted method was elaborated for the catalyst-free synthesis of isoindolin-1-one-3-phosphonates by the three-component condensation of 2-formylbenzoic acid, aliphatic primary amines and various dialkyl phosphites. The batch and the continuous flow reactions were optimized in respect of the temperature, the reaction time and the molar ratio of the starting materials. To evaluate the potential of MW irradiation, comparative thermal experiments were also carried out. In order to obtain “real time” information about the condensation, the special Kabachnik–Fields reaction of 2-formylbenzoic acid, butylamine and diethyl phosphite was monitored by in situ FT-IR spectroscopy. The novel title compounds could be prepared in high yields at low temperature under a short reaction time. A suitable method could also be developed for the preparation of the isoindolin-1-one-3-phosphonates at a “few g” scale by using a continuous flow MW reactor.

## 1. Introduction

**Figure 1:**
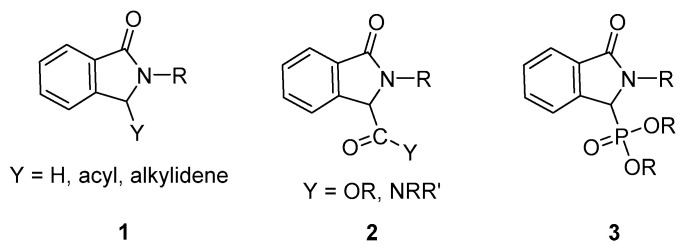
General formula of isoindolin-1-one derivatives (**1**), isoindolin-1-one carboxylates (**2**) and isoindolin-1-one phosphonates (**3**).

Isoindolin-1-ones (**1**) and their derivatives, such as 3-acyl- and 3-alkylidene-isoindolin-1-ones, may be considered as an important compound family among *N*-heterocycles [1,2,3,4] (Figure 1). This moiety can be found in a number of biologically active natural compounds and synthetic derivatives as well [5,6,7]. As a special subgroup, isoindolin-1-ones bearing a carboxylic ester or carboxylic amide function (**2**) may show anticancer- [5], antiarrhythmic- [8] or sodium channel modulator activities [9]. It is known that phosphorus analogues of biologically active carboxylic acid derivatives may often show important effects, e.g., α-aminophosphonates and α-amino acids [10]. Isoindolin-1-one phosphonates (**3**)—the P-analogues of compound **2**—have been tried out as plant growth regulators [11].

**Scheme 1:**
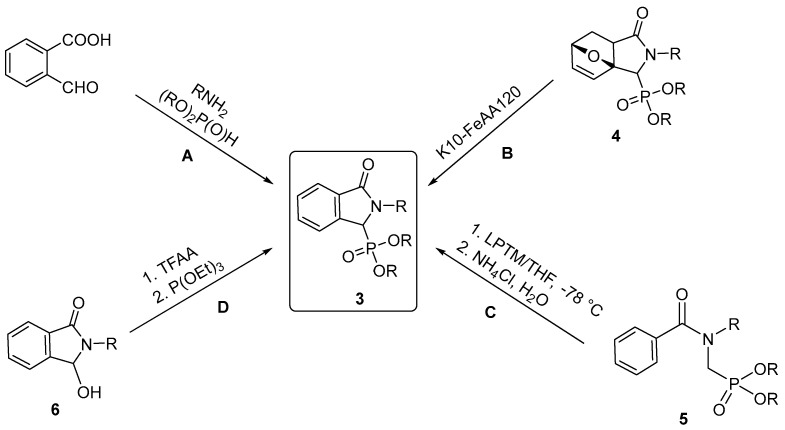
Synthetic routes of isoindolin-1-one phosphonates (**3**).

Only a few examples have been reported on the synthesis of isoindolin-1-one phosphonates by the three-component condensation of 2-formylbenzoic acid, amines and dialkyl phosphites (Scheme 1/**A**) [7,12,13,14,15,16,17]. This process is somewhat analogous to the ‘regular’ Kabachnik–Fields reaction [18]; however, the carboxyl group of the 2-formylbenzoic acid allows a special ring closure step, as the speciality of this transformation. Ordóñez and co-workers carried out the condensation of 2-formylbenzoic acid, dimethyl phosphite and aromatic amines or aminoacetaldehyde dimethyl acetal under solvent-free conditions or in methanol in a microwave (MW) reactor [12,13]. The special Kabachnik–Fields reaction starting from amino alcohols [14] or chiral amines was also investigated using dimethyl phosphite as the phosphorus reagent [15]. In a few cases, the transformations were investigated in the presence of NaH [16], T_3_P^®^ [17] or OSU-6 [7] as catalysts or additives. To the best of our knowledge, the three-component reaction of 2-formylbenzoic acid, aliphatic amines (butyl-, cyclohexyl- or benzylamine) and dialkyl phosphites, such as diisopropyl-, dibutyl- or dibenzyl phosphite, has not been studied comprehensively before.

Beside the three-component condensation, a few other synthetic routes are also known for the preparation of isoindolin-1-one phosphonates (**3**), such as the ring opening of tricyclic oxanorbornenes (**4**) (Scheme 1/**B**) [19], the ring closure of α-amidophosphonates (**5**) (Scheme 1/**C**) [20] or the reaction of trialkyl phosphites with 3-hydroxy-isoindolin-1-ones (**6**) (Scheme 1/**D**) [21,22]. In these examples, the target compounds (**3**) were prepared starting from complex molecules (**4**–**6**), which were usually obtained after many reaction steps.

The application of the MW technique may be advantageous in several organic reactions and organophosphorus transformations as well [23]. The reactions may proceed faster and the use of a catalyst and/or a solvent is often evitable [24]. In case of multicomponent reactions, the MW irradiation may also be useful, as the corresponding products can usually be obtained more selectively. Therefore, the development of MW-assisted variations of multicomponent reactions is of growing interest [25]. The scale-up of MW-assisted transformations is a real challenge due to the limited geometry of the MW unit [26]. This problem can be solved by the use of continuous flow MW reactors [27]. In the last decade, various applications of the continuous flow MW technique were reported; however, in most cases non-professional MW reactors were applied, in which the parameters cannot be measured accurately [28]. In our previous work, we introduced a MW-assisted continuous flow synthesis of simple α-aryl-α-aminophosphonates by the three-component Kabachnik–Fields reaction [29], which may mean a good starting point towards the development of special condensation-ring closure reactions in continuous flow.

In this paper, at first, the catalyst- and solvent-free condensation of 2-formylbenzoic acid, aliphatic primary amines and various >P(O)H reagents was studied in a batch MW reactor. Our aim was to optimize the process and to synthesize and characterize new derivatives. The effect of the MW irradiation was also investigated; thus, comparative thermal experiments were also carried out. We also aimed at the monitoring of the special Kabachnik–Fields reaction by in situ FT-IR spectroscopy. Furthermore, we wished to develop a continuous flow MW-assisted method for the three-component reaction of 2-formylbenzoic acid, aliphatic amines and diethyl phosphite to obtain the corresponding isoindolin-1-one-3-phosphonates at a higher productivity.

## 2. Results and Discussion

### 2.1. Synthesis of Isoindolin-1-one-3-phosphonates

As the initial step of our work, the special Kabachnik–Fields reaction of 2-formylbenzoic acid, aliphatic primary amines, such as butyl-, cyclohexyl- or benzylamine, and diethyl phosphite was studied under catalyst- and solvent-free conditions (Table 1). The condensations were optimized in respect of the molar ratio of the starting materials, the temperature and the reaction time.

At first, the 2-formylbenzoic acid was reacted with butylamine and diethyl phosphite in a molar ratio of 1:1:1 at room temperature for 3 h, and the conversion was only 77% (Table 1, Entry 1). Carrying out the reaction at a higher temperature of 40 °C under MW irradiation, 61% of diethyl (2-butyl-3-oxo-2,3-dihydro-1*H*-isoindol-1-yl)phosphonate (**7a**) was obtained after 10 min (Table 1, Entry 2). Repeating the condensation at 60 °C for 10 min, the conversion increased to 85% (Table 1, Entry 3). Applying a higher temperature of 80 °C for the same reaction time, the conversion did not change significantly (Table 1, Entry 4). Performing the condensation at 60 °C using longer reaction times (20 or 30 min), the proportion of the isoindolin-1-one-3-phosphonate (**7a**) was somewhat higher (90% and 91%, respectively) as compared to the reaction carried out for 10 min (Table 1, Entries 5 and 6 vs. Entry 3). In the next experiments, the effect of the molar ratio of the starting materials was studied at 60 °C applying a reaction time of 10 min (Table 1, Entries 7–10). Increasing the amount of diethyl phosphite to 1.1 or 1.2 equivalents had no effect on the conversion (Table 1, Entry 3 vs. Entries 7 and 8). However, by using a small excess of butylamine (1.1 equivalents), an increase in the conversion was observed, and applying 1.2 equivalents of the amine component, a complete conversion could be achieved (Table 1, Entries 9 and 10). The diethyl (2-butyl-3-oxo-2,3-dihydro-1*H*-isoindol-1-yl)phosphonate (**7a**) was isolated in a yield of 94% (Table 1, Entry 10). To evaluate the potential of the MW irradiation, the same reaction was repeated using conventional heating, resulting in a conversion of only 90% (Table 1, Entry 11).

Carrying out the condensation of the 2-formylbenzoic with 1.2 equivalents of cyclohexylamine and 1 equivalent of diethyl phosphite at 60 °C for 10 min, 82% of diethyl (2-cyclohexyl-3-oxo-2,3-dihydro-1*H*-isoindol-1-yl)phosphonate (**8a**) was formed (Table 1, Entry 12). After an irradiation of 20 min, 93% of **8a** was present in the reaction mixture (Table 1, Entry 13), while after 30 min, a complete conversion was achieved (Table 1, Entry 14). The corresponding isoindolin-1-one-3-phosphonate (**8a**) could be obtained in a yield of 84% after column chromatography. The comparative thermal experiment reached a conversion of only 84% (Table 1, Entry 15).

Applying benzylamine, a reaction time of 10 min was also not enough for a complete conversion; however, after 20 min the proportion of the diethyl (2-benzyl-3-oxo-2,3-dihydro-1*H*-isoindol-1-yl)phosphonate (**9a**) was 100% (Table 1, Entries 16 and 17). Product **9a** was isolated in a yield of 90%. The thermal reaction was somewhat slower, the conversion was only 88% after 20 min (Table 1, Entry 18).

Based on the results obtained, all the three aliphatic amines allowed fast transformation towards the desired isoindolin-1-one phosphonates (**7a**–**9a**). The butylamine was the most reactive amine, as the special Kabachnik–Fields reaction was already complete after 10 min, and the cyclohexylamine was the less reactive, possibly due to the steric hindrance of the bulky cyclohexyl group. The conversions in the comparative thermal experiments were by 10–16% lower, which clearly indicates the efficiency of the MW technique.

**Scheme 2:**
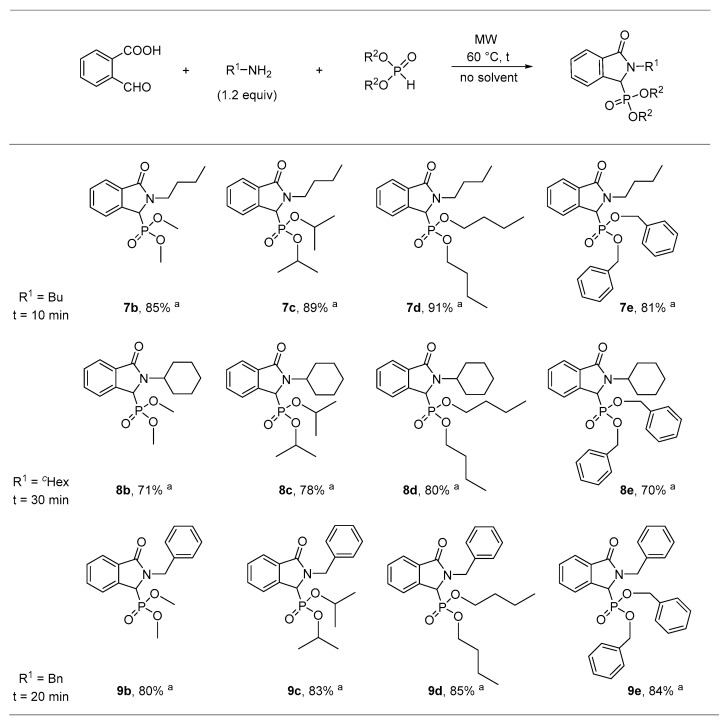
Microwave (MW)-assisted condensation of 2-formylbenzoic acid, primary amines and dialkyl phosphites. ^a^ Isolated yield.

In the next series of experiments, the three-component condensation of 2-formylbenzoic acid, primary amines and various dialkyl phosphites was studied using the optimized conditions (60 °C, 10–30 min) (Scheme 2). The reactions were complete in all cases. Carrying out the condensation of 2-formylbenzoic acid and butylamine with dimethyl phosphite, the dimethyl (2-butyl-3-oxo-2,3-dihydro-1*H*-isoindol-1-yl)phosphonate (**7b**) was obtained in a yield of 85% after column chromatography. Changing for diisopropyl- or dibutyl phosphite, the corresponding isoindolin-1-one-3-phosphonates (**7c** and **7d**) were synthesized in yields of 89% and 91%, respectively. Dibenzyl phosphite was also tried out as the P-reagent. In this case, the corresponding isoindolin-1-one phosphonate (**7d**) could be prepared in a slightly lower yield (81%). Performing the experiments starting from cyclohexylamine, the condensations were slower (30 min), and the desired isoindolin-1-one-3-phosphonates (**8b**–**e**) were isolated in yields of 71–80%. The condensation was also extended for benzylamine, obtaining isoindolin-1-one phosphonates **9b**–**e** in high yields (80–85%).

**Scheme 3:**
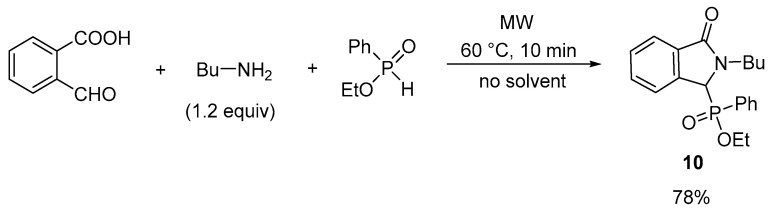
Microwave-assisted condensation of 2-formylbenzoic acid, butylamine and ethyl phenyl-*H*-phosphinate.

Finally, the reaction of 2-formylbenzoic acid and butylamine was also carried out using ethyl phenyl-*H*-phosphinate as the P-reagent at 60 °C, for an irradiation of 10 min (Scheme 3). After column chromatography, product **10** was isolated in a yield of 78%. Due to the chiral center on the phosphorus atom, the isoindolin-1-one phosphinate (**10**) was obtained as a mixture of diastereomers in a ratio of 47:53 based on ^31^P NMR spectroscopy.

Altogether 16 isoindolin-1-one-3-phosphonate derivatives were synthesized in high yields at low temperature for a short reaction time, and characterized by ^31^P, ^1^H and ^13^C NMR spectroscopy, as well as by HRMS. Except products **9a** and **9b**, all derivatives are new compounds. (Copies of ^31^P, ^1^H, and ^13^C NMR spectra for all compounds synthesized are presented in the Supplementary Materials.)

### 2.2. Study on the Condensation of 2-Formylbenzoic Acid, Butylamine and Diethyl Phosphite by In Situ FT-IR Spectroscopy

**Scheme 4:**
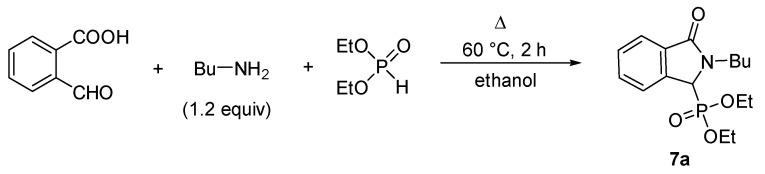
Condensation of 2-formylbenzoic acid, butylamine and diethyl phosphite in ethanol.

The condensation of 2-formylbenzoic acid, butylamine and diethyl phosphite was followed by in situ Fourier transform (FT)-IR spectroscopy (Scheme 4). The reaction was carried out under conventional heating at 60 °C in ethanol as the solvent.

**Figure 2:**
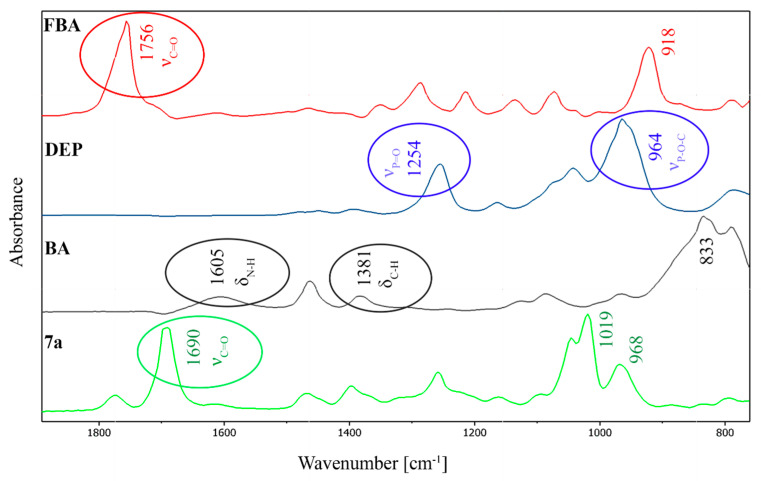
IR spectra of the reaction components in ethanol (absorptions of the solvent were subtracted for clarity).

The first step was to register the IR spectra of the reaction components in ethanol solution (Figure 2). 2-Formylbenzoic acid (**FBA**) has a strong absorption band at 1756 cm^−1^ corresponding to the ν_C=O_. The single ν_C=O_ absorption suggests that **FBA** is present in its lactone tautomer form in ethanol [30,31]. At the same time, diethyl phosphite (**DEP**) has strong signals at 964 cm^−1^ and 1254 cm^−1^ assigned to the ν_P-O-C_ and the ν_P=O_ vibrations, respectively. Butylamine (**BA**) may be identified based on the δ_C-H_ (1381 cm^−1^) and δ_N-H_ (1605 cm^−1^) absorptions. In the spectrum of diethyl (2-butyl-3-oxo-2,3-dihydro-1*H*-isoindol-1-yl)phosphonate (**7a**), a strong characteristic ν_C=O_ signal can be observed at 1690 cm^−1^. 

**Figure 3:**
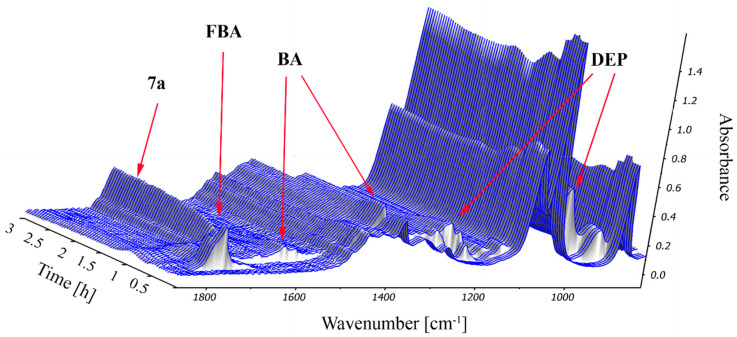
A segment of the time-dependent IR spectrum of the condensation of 2-formylbenzoic acid (**FBA**), butylamine (**BA**) and diethyl phosphite (**DEP**) in ethanol.

In the in situ FT-IR investigation, the starting materials were mixed in ten-minute intervals in the following order: 1. **FBA**, 2. **DEP,** 3. **BA**. Then, the mixture was heated to 60 °C in an oil bath and was reacted until completion. A segment of the time-dependent IR spectrum (3D diagram) can be seen in Figure 3. The reaction components (**FBA**, **DEP**, **BA** and **7a**) could be easily identified by their characteristic absorptions (Figure 2).

**Figure 4:**
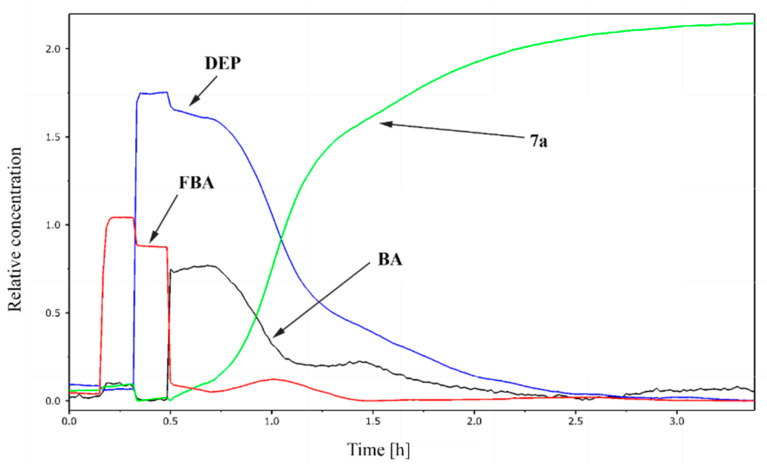
Concentration profiles of the reaction components in the condensation of 2-formylbenzoic acid **(FBA**), butylamine (**BA**) and diethyl phosphite (**DEP**) in ethanol.

The result of our study is the diagram showing the concentration profile of the starting components (**FBA**, **DEP** and **BA**) and the product (**7a**) (Figure 4). This was constructed by deconvolution on the basis of the decrease/increase of the different absorptions on the time scale. This calculation (MCR-ALS, multivariate curve resolution-alternating least squares) gives the concentration profiles of the components and the spectra of pure components. It can be seen—also in the 3D diagram (Figure 3)—that the signal of **FBA** immediately decreased after adding **BA**; however, no new significant absorptions could be observed, which would indicate the formation of an intermediate, such as an imine [32]. Decreasing of the signals is possibly due to the change of IR properties of **FBA** in the presence of **BA**. During the reaction, the relative concentrations of the starting materials were constantly decreasing, while the signal of product **7a** was constantly increasing. The condensation was complete at ca. 2 h. 

**Figure 5:**
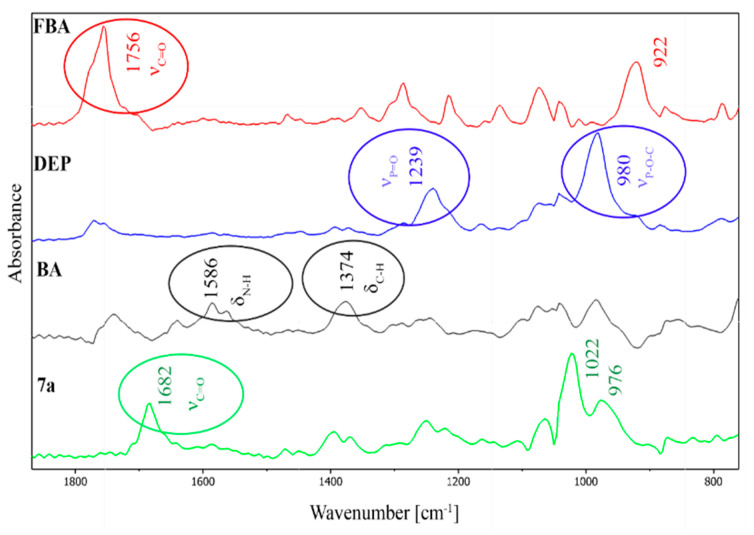
IR spectra of the reaction components obtained after deconvolution.

Finally, the IR spectra of the reaction components (**FBA**, **DEP**, **BA** and **7a**) were extracted from the calculation (Figure 5). As compared to the spectra obtained in ethanol solution (Figure 2), the agreement is rather good.

### 2.3. Condensation of 2-Formylbenzoic Acid, Aliphatic Primary Amines and Diethyl Phosphite in a Continuous Flow Microwave Reactor

**Scheme 5:**
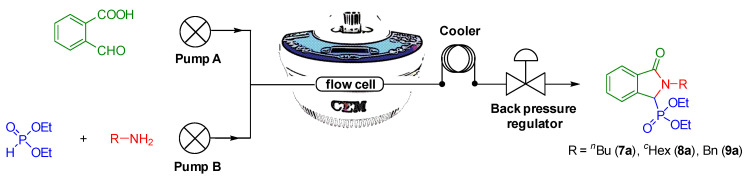
Condensation of 2-formylbenzoic acid, aliphatic primary amines and diethyl phosphite in a continuous flow microwave reactor.

In order to increase the productivity of the MW-assisted synthesis developed, the reaction of 2-formylbenzoic acid, aliphatic primary amines and diethyl phosphite was studied using a dual pump continuous flow system containing a CEM^®^ (Matthews, NC, USA) MW reactor equipped with a commercially available CEM^®^ continuous flow cell (Figures S1 and S2) (Scheme 5, Table 2).

The 2-formylbenzoic acid in ethanol (solution A) and the mixture of the corresponding amine and diethyl phosphite in ethanol (solution B) were fed separately into the mixer. The mixture reached the reactor at a summa flow rate of 0.15–0.70 mL/min (corresponding to residence times of 45–10 min, respectively). The temperature was monitored and controlled by the IR sensor of the MW device. The mixture leaving the reactor was cooled down to 25 °C using a spiral-like cooler and was passed through a back pressure regulator operating at 250 psi (17 bar). Consecutive fractions of the leaving mixture were analyzed by GC measurements to determine the composition and to identify the stationary operation.

Performing the condensation using 1.2 equivalents of the butylamine at 60 °C at a flow rate of 0.7 mL/min (corresponding to a residence time of 10 min), the conversion was 43% (Table 2, Entry 1). Increasing the residence time to 20 min (by decreasing the flow rate to 0.35 mL/min), the proportion of the isoindolin-1-one-3-phosphonate (**7a**) was somewhat higher (52%) (Table 2, Entry 2). The condensation was also carried out at a residence time of 30 min (at a flow rate of 0.25 mL/min); however, the conversion increased by only 4% (Table 2, Entry 3). In the next series of experiments, the effect of the molar ratio of the starting materials was investigated. Applying 1.5 equivalents of the butylamine at a residence time of 20 min, the conversion already increased to 70% (Table 2, Entry 4). At a higher excess of butylamine (2 equivalents), the outcome did not change significantly (Table 2, Entry 5). Carrying out the condensation with 1.2 or 1.5 equivalents of diethyl phosphite, the ratio of the isoindolin-1-one-3-phosphonate (**7a**) was 81% and 95%, respectively (Table 2, Entries 6 and 7). Using 1.5 equivalents of both of the butylamine and the diethyl phosphite, the reaction was complete under a residence time of 30 min, and the diethyl (2-butyl-3-oxo-2,3-dihydro-1*H*-isoindol-1-yl)phosphonate (**7a**) was isolated in a yield of 95% (Table 2, Entry 8).

Performing the continuous flow three-component reaction with cyclohexylamine at the same temperature (60 °C), the condensation was somewhat slower, as it was complete applying a residence time of 45 min (at a flow rate of 0.15 mL/min) (Table 2, Entry 9). The corresponding isoindolin-1-one-3-phosphonate (**8a**) was obtained in a yield of 86% after column chromatography.

In case of benzylamine, a flow rate of 0.18 mL/min (a residence time of 40 min) at 60 °C were the optimal parameters (Table 2, Entry 10). The diethyl (2-benzyl-3-oxo-2,3-dihydro-1*H*-isoindol-1-yl)phosphonate (**9a**) could be isolated in a yield of 91%. 

In the flow approaches, the productivity was 2.3 g/h for product **7a**, 1.4 g/h for product **8a**, and 1.8 g/h in case of compound **9a**, which are higher than the batch results (1.8 g/h, 0.6 g/h and 1.0 g/h, respectively). However, it should be noted that the batch productivity was calculated based on the net reaction time of several (six, two and three, respectively) consecutive reactions - apart from the necessary preparations of the experiments.

## 3. Materials and Methods 

### 3.1. General

The reactions were carried out in a 300 W CEM Discover focused microwave reactor (CEM Microwave Technology Ltd., Buckingham, UK) equipped with a pressure controller using 5–20 W irradiation under isothermal conditions.

The continuous flow reactions were performed in a self-developed continuous flow system comprising a 300 W CEM Discover (CEM Microwave Technology Ltd., Buckingham, UK) focused microwave reactor equipped with a CEM 10 mL Flow Cell Accessory continuous flow unit (irradiated volume 7 mL) [29,33,34], a Gilson 332 dual HPLC pump (Gilson U.K., Bedfordshire, UK), an HPLC backpressure regulator with a 250 Psi (17.2 bar) cartridge (CEM Microwave Technology Ltd., Buckingham, UK) and a cooler. Teflon^®^ PFA tubes (DuPont, Wilmington, DE, USA) with outside diameter 0.125” (3.175 mm) and inside diameter 0.064” (1.575 mm) were used. The exact lengths and volumes of each tube parts are shown in Figure S1. All of the tubes, nuts and ferrules applied were fully compatible with a regular HPLC system.

GC measurements were performed on an Agilent^®^ 6890 GC-FID chromatograph (Agilent, Santa Clara, CA, USA), using a 15 m × 0.18 mm Restek, Rtx-5 column with a film layer of 0.20 μm. The temperature of the column was initially held at 40 °C for 1 min, followed by programming at 25 °C/min up to 300 °C and a final period at 300 °C (isothermal) for 10 min. The temperature of the injector was 290 °C and of the FID detector 300 °C. The carrier gas was N_2_.

The ^31^P, ^1^H, ^13^C, NMR spectra were taken in CDCl_3_ solution on a Bruker AV-300 spectrometer (Bruker Scientific LLC, Billerica, MA, USA) operating at 121.5, 300 and 75.5 MHz, respectively. Chemical shifts are downfield relative to 85% H_3_PO_4_ and TMS. Couplings are given in Hz. Non-equivalence effects were observed in ^1^H and ^13^C{^1^H} NMR spectra. Corresponding pairs of resonances were marked with (I) and (II), respectively. 

High resolution mass spectrometric measurements were performed using a Sciex 5600+ Q-TOF mass spectrometer (AB Sciex UK Limited, Warrington, UK) in positive electrospray mode.

In situ FT IR measurements were conducted using ReactIR 1000 equipment (Mettler-Toledo Inc., Columbus, OH, USA). An attenuated total reflectance (ATR) diamond probe was placed in a 100 mL four-necked flask equipped with a dropping funnel, a condenser, a thermometer and a magnetic stirrer. The temperature was maintained by using an appropriately adjusted oil bath.

### 3.2. General Procedure for the Synthesis of Isoindolin-1-One-3-Phosphonates (***7**–**9**)* and Isoindolin-1-One-3-Phosphinate *(**10**)*

A mixture of 1.0 mmol (0.15 g) 2-formylbenzoic acid, 1.0 mmol (0.10 mL), 1.1 mmol (0.11 mL) or 1.2 mmol (0,12 mL) butylamine, 1.2 mmol (0.14 mL) cyclohexylamine or 1.2 mmol (0.13 mL) benzylamine and >P(O)H derivative (1.0 mmol (diethyl phosphite (0.13 mL), dimethyl phosphite (0.09 mL), diisopropyl phosphite (0.17 mL), dibutyl phosphite (0.20 mL), dibenzyl phosphite (0.22 mL), ethyl phenyl-*H*-phosphinate (0.15 mL) or 1.1 mmol (0.14 mL) or 1.2 mmol (0.16 mL) diethyl phosphite was irradiated in a sealed tube at 40–60 °C for 10–30 min in a CEM Microwave reactor equipped with a pressure controller. The volatile components were removed in vacuum, and the residue was analysed by gas chromatography. The isoindolin-1-one-3-phosphonates and isoindolin-1-one-3-phosphinate were obtained by column chromatography using silica gel as the absorbent and dichloromethane:methanol (97:3) as the eluent. The following products were thus prepared:

#### 3.2.1. Diethyl (2-Butyl-3-oxo-2,3-dihydro-1*H*-isoindol-1-yl)phosphonate (**7a**)

Yield: 94% (0.31 g), colourless oil; ^31^P (CDCl_3_) δ 18.3; ^1^H NMR (CDCl_3_) δ 0.94 (t, *J*_HH_ = 7.4, 3H, C*H*_3_(CH_2_)_3_N), 1.12 (t, *J*_HH_ = 7.1, 3H, C*H*_3_CH_2_O^I^), 1.27 (t, *J*_HH_ = 7.1, 3H, C*H*_3_CH_2_O^II^), 1.29–1.38 (m, 2H, C*H*_2_(CH_2_)_2_N), 1.56–1.65 (m, 1H, CH_A_, C*H*_2_CH_2_N), 1.65–1.75 (m, 1H, CH_B_, C*H*_2_CH_2_N), 3.53–3.60 (m, 1H, CH_A_, CH_2_N), 3.76–3.85 (m, 1H, CH_A_, CH_2_O^I^), 3.91–4.00 (m, 1H, CH_B_, CH_2_O^I^), 4.08–4.18 (m, 3H, CH_B_, CH_2_N, CH_2_O^II^), 4.89 (d, *J*_HP_ = 13.6, 1H, C_1_H), 7.51 (t, *J*_HH_ = 7.5, 1H, C_5_H), 7.58 (t, *J*_HH_ = 8.0, 1H, C_6_H), 7.78 (d, *J*_HH_ = 7.6, 1H, C_7_H), 7.87 (d, *J*_HH_ = 7.5, 1H, C_4_H); ^13^C NMR (CDCl_3_) δ 13.8 (*C*H_3_(CH_2_)_3_N), 16.2 (d, ^3^*J*_CP_ = 5.6, *C*H_3_CH_2_O^I^), 16.4 (d, ^3^*J*_CP_ = 5.6,*C*H_3_CH_2_O^II^), 20.0 (*C*H_2_(CH_2_)_2_N), 30.0 (*C*H_2_CH_2_N), 41.5 (CH_2_N), 57.2 (d, ^1^*J*_CP_ = 155.0, C_1_), 63.2 (d, ^2^*J*_CP_ = 7.3, CH_2_O^I^), 63.5 (d, ^2^*J*_CP_ = 7.1, CH_2_O^II^), 123.8 (d, *J*_CP_ = 1.6, C_4_), 124.5 (d, ^3^*J*_CP_ = 2.6, C_7_), 128.9 (d, *J*_CP_ = 2.2, C_5_), 131.5 (d, *J*_CP_ = 2.5, C_6_), 132.5 (d, ^3^*J*_CP_ = 4.3, C_3a_), 138.5 (d, ^2^*J*_CP_ = 5.8, C_7a_), 168.8 (d, ^3^*J*_CP_ = 3.5, C_3_); [M + H]^+^_found_ = 326.1509, C_16_H_25_NO_4_P requires 326.1521.

#### 3.2.2. Dimethyl (2-Butyl-3-oxo-2,3-dihydro-1*H*-isoindol-1-yl)phosphonate (**7b**)

Yield: 85% (0.25 g), white crystals; Mp: 77–78 °C; ^31^P (CDCl_3_) δ 18.5; ^1^H NMR (CDCl_3_) δ 0.94 (t, *J*_HH_ = 7.3, 3H, C*H*_3_(CH_2_)_3_N), 1.22–1.45 (m, 2H, C*H*_2_(CH_2_)_2_N), 1.49–1.81 (m, 2H, C*H*_2_CH_2_N), 3.42–3.64 [3.56 (d, *J*_HH_ = 10.9, CH_3_O^I^) overlapped by the multiplet of CH_A_, CH_2_N total int. 4H], 3.75 (d, *J*_HH_ = 10.9, 3H, CH_3_O^II^), 4.03–4.24 (m, 1H, CH_B_, CH_2_N), 4.91 (d, *J*_HP_ = 13.4, 1H, C_1_H), 7.52 (t, *J*_HH_ = 6.9, 1H, C_5_H), 7.59 (t, *J*_HH_ = 6.4, 1H, C_6_H), 7.77 (d, *J*_HH_ = 7.5, 1H, C_7_H), 7.88 (d, *J*_HH_ = 7.4, 1H, C_4_H); ^13^C NMR (CDCl_3_) δ 13.7 (*C*H_3_(CH_2_)_3_N), 19.9 (*C*H_2_(CH_2_)_2_N), 29.9 (*C*H_2_CH_2_N), 41.5 (CH_2_N), 53.6 (d, ^2^*J*_CP_ = 7.3, CH_3_O^I^), 53.7 (d, ^2^*J*_CP_ = 7.1, CH_3_O^II^), 56.7 (d, ^1^*J*_CP_ = 155.4, C_1_), 123.8 (d, *J*_CP_ = 1.6, C_4_), 124.3 (d, ^3^*J*_CP_ = 2.7, C_7_), 128.9 (d, *J*_CP_ = 2.2, C_5_), 131.5 (d, *J*_CP_ = 2.5, C_6_), 132.3 (d, ^3^*J*_CP_ = 4.1, C_3a_), 138.2 (d, ^2^*J*_CP_ = 5.9, C_7a_), 168.6 (d, ^3^*J*_CP_ = 3.4, C_3_); [M + H]^+^_found_ = 298.1196, C_14_H_21_NO_4_P requires 298.1208.

#### 3.2.3. Diisopropyl (2-Butyl-3-oxo-2,3-dihydro-1*H*-isoindol-1-yl)phosphonate (**7c**)

Yield: 89% (0.32 g), colourless oil; ^31^P (CDCl_3_) δ 16.6; ^1^H NMR (CDCl_3_) δ 0.92 (t, *J*_HH_ = 7.3, 3H, C*H*_3_(CH_2_)_3_N), 0.95 (d, *J*_HH_ = 7.3, 3H, C*H*_3_CH^I^), 1.19–1.41 [1.24 (d, *J*_HH_ = 6.2, C*H*_3_CH^II^), 1.25 (d, *J*_HH_ = 6.2, C*H*_3_CH^III^), 1.28 (d, *J*_HH_ = 6.1, C*H*_3_CH^IV^) overlapped by the multiplet of C*H*_2_(CH_2_)_2_N total int. 11H], 1.52–1.79 (m, 2H, C*H*_2_CH_2_N), 3.51–3.65 (m, 1H, CH_A_, CH_2_N), 4.06–4.20 (m, 1H, CH_B_, CH_2_N), 4.32–4.49 (m, 1H, CHO^I^), 4.66–4.80 (m, 1H, CHO^II^), 4.84 (d, *J*_HP_ = 13.8, 1H, C_1_H), 7.50 (t, *J*_HH_ = 7.3, 1H, C_5_H), 7.56 (t, *J*_HH_ = 6.8, 1H, C_6_H), 7.79 (d, *J*_HH_ = 7.5, 1H, C_7_H), 7.86 (d, *J*_HH_ = 7.2, 1H, C_4_H); ^13^C NMR (CDCl_3_) δ 13.8 (*C*H_3_(CH_2_)_3_N), 20.0 (*C*H_2_(CH_2_)_2_N), 23.3 (d, ^3^*J*_CP_ = 5.3, *C*H_3_CH^I^), 23.9 (d, ^3^*J*_CP_ = 5.0, *C*H_3_CH^II^), 24.06 (d, ^3^*J*_CP_ = 6.6, *C*H_3_CH^III^), 24.10 (d, ^3^*J*_CP_ = 6.1, *C*H_3_CH^IV^), 30.0 (*C*H_2_CH_2_N), 41.5 (CH_2_N), 57.1 (d, ^1^*J*_CP_ = 155.0, C_1_), 63.1 (d, ^2^*J*_CP_ = 7.3, CHO^I^), 63.4 (d, ^2^*J*_CP_ = 7.1, CHO^II^), 123.7 (d, *J*_CP_ = 1.6, C_4_), 124.4 (d, ^3^*J*_CP_ = 2.6, C_7_), 128.8 (d, *J*_CP_ = 2.2, C_5_), 131.4 (d, *J*_CP_ = 2.5, C_6_), 132.4 (d, ^3^*J*_CP_ = 4.3, C_3a_), 138.4 (d, ^2^*J*_CP_ = 5.8, C_7a_), 168.7 (d, ^3^*J*_CP_ = 3.5, C_3_); [M + H]^+^_found_ = 354.1826, C_18_H_29_NO_4_P requires 354.1828.

#### 3.2.4. Dibutyl (2-Butyl-3-oxo-2,3-dihydro-1*H*-isoindol-1-yl)phosphonate (**7d**)

Yield: 91% (0.35 g), colourless oil; ^31^P (CDCl_3_) δ 18.3; ^1^H NMR (CDCl_3_) δ 0.82 (t, *J*_HH_ = 7.3, 3H, C*H*_3_(CH_2_)_3_O^I^), 0.89 (t, *J*_HH_ = 7.2, 3H, C*H*_3_(CH_2_)_3_O^II^), 0.94 (t, *J*_HH_ = 7.3, 3H, C*H*_3_(CH_2_)_3_N), 1.13–1.49 (m, 8H, C*H*_2_(CH_2_)_2_N, C*H*_2_(CH_2_)_2_O, C*H*_2_CH_2_O^I^), 1.49–1.79 (m, 4H, C*H*_2_CH_2_N, C*H*_2_CH_2_O^II^), 3.46–3.62 (m, 1H, CH_A_, CH_2_N), 3.63–3.78 (m, 1H, CH_A_, CH_2_O^I^), 3.81–3.95 (m, 1H, CH_B_, CH_2_O^I^), 3.96–4.24 (m, 3H, CH_B_, CH_2_N, CH_2_O^II^), 4.89 (d, *J*_HP_ = 13.6, 1H, C_1_H), 7.51 (t, *J*_HH_ = 7.4, 1H, C_5_H), 7.57 (t, *J*_HH_ = 7.4, 1H, C_6_H), 7.78 (d, *J*_HH_ = 7.5, 1H, C_7_H), 7.86 (d, *J*_HH_ = 7.3, 1H, C_4_H); ^13^C NMR (CDCl_3_) δ 13.41 (*C*H_3_(CH_2_)_3_O^I^), 13.43 (*C*H_3_(CH_2_)_3_O^II^), 13.7 (*C*H_3_(CH_2_)_3_N), 18.5 (*C*H_2_(CH_2_)_2_O^I^), 18.6 (*C*H_2_(CH_2_)_2_O^II^), 20.0 (*C*H_2_(CH_2_)_2_N), 30.0 (*C*H_2_CH_2_N), 32.3 (d, ^3^*J*_CP_ = 5.6, *C*H_2_CH_2_O^I^), 32.4 (d, ^3^*J*_CP_ = 5.9, *C*H_2_CH_2_O^II^), 41.5 (CH_2_N), 57.1 (d, ^1^*J*_CP_ = 155.0, C_1_), 66.7 (d, ^2^*J*_CP_ = 7.5, CH_2_O^I^), 67.1 (d, ^2^*J*_CP_ = 7.2, CH_2_O^II^), 123.7 (d, *J*_CP_ = 1.4, C_4_), 124.4 (d, ^3^*J*_CP_ = 2.7, C_7_), 128.8 (d, *J*_CP_ = 2.2, C_5_), 131.4 (d, *J*_CP_ = 2.4, C_6_), 132.5 (d, ^3^*J*_CP_ = 4.3, C_3a_), 138.5 (d, ^2^*J*_CP_ = 5.8, C_7a_), 168.6 (d, ^3^*J*_CP_ = 3.3, C_3_); [M + H]^+^_found_ = 382.2131, C_20_H_33_NO_4_P requires 382.2147.

#### 3.2.5. Dibenzyl (2-Butyl-3-oxo-2,3-dihydro-1*H*-isoindol-1-yl)phosphonate (**7e**)

Yield: 81% (0.36 g), white crystals; Mp: 103–104 °C; ^31^P (CDCl_3_) δ 19.2; ^1^H NMR (CDCl_3_) δ 0.85 (t, *J*_HH_ = 7.3, 3H, C*H*_3_(CH_2_)_3_N), 1.13–1.30 (m, 2H, C*H*_2_(CH_2_)_2_N), 1.41–1.65 (m, 2H, C*H*_2_CH_2_N), 3.37–3.56 (m, 1H, CH_A_, CH_2_N), 3.96–4.14 (m, 1H, CH_B_, CH_2_N), 4.61–4.76 (m, 1H, C_1_H), 4.76–4.90 (m, 2H, CH_2_O^I^), 4.90–5.06 (m, 2H, CH_2_O^II^), 7.10–7.24 (m, 4H, C_2′_H), 7.24–7.57 (m, 6H, C_3,4′_H), 7.43–7.57 (m, 2H, C_5_H, C_6_H), 7.73 (d, *J*_HH_ = 7.2, 1H, C_7_H), 7.81 (d, *J*_HH_ = 7.0, 1H, C_4_H); ^13^C NMR (CDCl_3_) δ 13.7 (*C*H_3_(CH_2_)_3_N), 19.9 (*C*H_2_(CH_2_)_2_N), 29.9 (*C*H_2_CH_2_N), 41.5 (CH_2_N), 57.4 (d, ^1^*J*_CP_ = 154.9, C_1_), 68.6 (d, ^2^*J*_CP_ = 7.3, CH_2_O^I^), 68.8 (d, ^2^*J*_CP_ = 7.0, CH_2_O^II^), 123.8 (d, *J*_CP_ = 1.6, C_4_), 124.4 (d, ^3^*J*_CP_ = 2.7, C_7_), 128.1 (C_3′_^I^), 128.2 (C_3′_^II^), 128.66 (C_2′_^I^), 128.67 (C_2′_^II^), 128.87 (C_4′_^I^), 128.90 (C_4′_^II^), 128.9 (d, *J*_CP_ = 2.4, C_5_), 131.4 (d, *J*_CP_ = 2.5, C_6_), 132.5 (d, ^3^*J*_CP_ = 4.2, C_3a_), 135.43 (d, ^3^*J*_CP_ = 5.5, C_1′_^I^), 135.44 (d, ^3^*J*_CP_ = 5.7, C_1′_^II^), 138.2 (d, ^2^*J*_CP_ = 6.0, C_7a_), 168.6 (d, ^3^*J*_CP_ = 3.5, C_3_); [M + H]^+^_found_ = 450.1822, C_26_H_29_NO_4_P requires 450.1828.

#### 3.2.6. Diethyl (2-Cyclohexyl-3-oxo-2,3-dihydro-1*H-*isoindol-1-yl)phosphonate (**8a**)

Yield: 84% (0.30 g), colourless oil; ^31^P (CDCl_3_) δ 18.7; ^1^H NMR (CDCl_3_) δ 1.14 (t, *J*_HP_ = 7.1, 3H, (C*H*_3_CH_2_O^I^), 1.28–1.45 [1.25 (t, *J*_HH_ = 7.1, C*H*_3_CH_2_O^II^) overlapped by the multiplet of *^c^*HexH total int. 6H], 1.57–2.03 (m, 5H, *^c^*HexH), 2.13–2.31 (m, 1H, *^c^*HexH), 2.36–2.55 (m, 1H, *^c^*HexH), 3.69–3.88 (m, 2H, CH_2_O^I^), 3.88–4.02 (m, 1H, C_1′_H), 4.02–4.19 (m, 2H, CH_2_O^II^), 4.85 (d, 1H, *J*_HP_ = 13.2, C_1_H), 7.49 (t, 1H, *J*_HH_ = 7.6, C_5_H), 7.55 (t, 1H, *J*_HH_ = 7.2, C_6_H), 7.74 (d, *J*_HH_ = 7.6, 1H, C_7_H), 7.82 (d, *J*_HH_ = 7.3, 1H, C_4_H); ^13^C NMR (CDCl_3_) δ 16.2 (d, ^3^*J*_CP_ = 4.6, *C*H_3_CH_2_O^I^), 16.3 (d, ^3^*J*_CP_ = 5.5, *C*H_3_CH_2_O^II^), 25.3 (C_4′_), 26.1 (C_3′_^I^), 26.3 (C_3′_^II^), 29.3 (C_2′_^I^), 29.7 (C_2′_^II^), 56.7 (C_1′_), 58.6 (d, ^1^*J*_CP_ = 155.4, C_1_), 63.2 (d, ^2^*J*_CP_ = 7.5, CH_2_O^I^), 63.4 (d, ^2^*J*_CP_ = 7.0, CH_2_O^II^), 123.4 (d, *J*_CP_ = 1.3, C_4_), 124.4 (d, ^3^*J*_CP_ = 2.7, C_7_), 128.7 (d, *J*_CP_ = 2.3, C_5_), 131.2 (d, *J*_CP_ = 2.6, C_6_), 133.6 (d, ^3^*J*_CP_ = 4.0, C_3a_), 138.8 (d, ^2^*J*_CP_ = 5.7, C_7a_), 169.0 (d, ^3^*J*_CP_ = 3.4, C_3_); [M+H]^+^_found_ = 352.1672, C_18_H_27_NO_4_P requires 352.1672.

#### 3.2.7. Dimethyl (2-Cyclohexyl-3-oxo-2,3-dihydro-1*H*-isoindol-1-yl)phosphonate (**8b**)

Yield: 71% (0.23 g), colourless oil; ^31^P (CDCl_3_) δ 20.9; ^1^H NMR (CDCl_3_) 1.18–1.45 (m, 3H, *^c^*HexH), 1.60–1.72 (m, 1H, *^c^*HexH), 1.72–2.02 (m, 4H, *^c^*HexH), 2.10–2.30 (m, 1H, *^c^*HexH), 2.31–2.50 (m, 1H, *^c^*HexH), 3.59 (d, *J*_HP_ = 10.7, 3H, CH_3_O^I^), 3.76–3.86 [3.72 (d, *J*_HP_ = 10.9, CH_3_O^II^) overlapped by the multiplet of C_1′_H total int. 4H], 4.88 (d, *J*_HP_ = 13.1, 1H, C_1_H), 7.44–7.61 (m, 2H, C_5_H, C_6_H), 7.71 (d, *J*_HH_ = 7.5, 1H, C_7_H), 7.83 (d, *J*_HH_ = 7.2, 1H, C_4_H); ^13^C NMR (CDCl_3_) δ 25.2 (C_4′_), 26.0 (C_3′_^I^), 26.3 (C_3′_^II^), 29.4 (C_2′_^I^), 29.7 (C_2′_^II^), 53.7 (d, ^2^*J*_CP_ = 7.2, CH_3_O^I^), 53.8 (d, ^2^*J*_CP_ = 7.2, CH_3_O^II^), 56.6 (C_1′_), 59.2 (d, ^1^*J*_CP_ = 156.4, C_1_), 123.5 (d, *J*_CP_ = 1.6, C_4_), 124.3 (d, ^3^*J*_CP_ = 2.6, C_7_), 128.8 (d, *J*_CP_ = 2.2, C_5_), 131.3 (d, *J*_CP_ = 2.5, C_6_), 133.4 (d, ^3^*J*_CP_ = 4.1, C_3a_), 138.5 (d, ^2^*J*_CP_ = 6.0, C_7a_), 169.0 (d, ^3^*J*_CP_ = 3.5, C_3_); [M + H]^+^_found_ = 324.1364, C_16_H_23_NO_4_P requires 324.1359.

#### 3.2.8. Diisopropyl (2-Cyclohexyl-3-oxo-2,3-dihydro-1*H*-isoindol-1-yl)phosphonate (**8c**)

Yield: 78% (0.30 g), white crystals; Mp: 112–113 °C; ^31^P (CDCl_3_) δ 17.1; ^1^H NMR (CDCl_3_) δ 0.96 (d, *J*_HH_ = 6.2, 3H, C*H*_3_CH^I^), 1.15–1.44 (1.23 [d, *J*_HH_ = 6.2, C*H*_3_CH^II^), 1.26 (d, *J*_HH_ = 6.1, C*H*_3_CH^III^, C*H*_3_CH^IV^) overlapped by the multiplet of *^c^*HexH total int. 12H], 1.57–2.03 (m, 5H, *^c^*HexH), 2.16–2.36 (m, 1H, *^c^*HexH), 2.38–2.59 (m, 1H, *^c^*HexH), 3.68–3.88 (m, 1H, C_1′_H), 4.30–4.49(m, 1H, CHO^I^), 4.64–4.85 [4.79 (d, *J*_HP_ = 13.5, C_1_H) overlapped by the multiplet of CHO^II^ total int. 2H], 7.42–7.58 [7.47 (t, *J*_HH_ = 7.4, C_5_H) overlapped by the multiplet of C_6_H total int. 2H], 7.75 (d, *J*_HH_ = 7.6, 1H, C_7_H), 7.81 (d, *J*_HH_ = 7.2, 1H, C_4_H); ^13^C NMR (CDCl_3_) δ 23.4 (d, ^3^*J*_CP_ = 5.3, *C*H_3_CH^I^), 23.9 (d, ^3^*J*_CP_ = 5.3, *C*H_3_CH^II^), 24.0 (d, ^3^*J*_CP_ = 3.5, *C*H_3_CH^III^), 24.1 (d, ^3^*J*_CP_ = 3.4, *C*H_3_CH^IV^), 25.2 (C_4′_), 26.1 (C_3′_^I^), 26.3 (C_3′_^II^), 29.2 (C_2′_^I^), 29.6 (C_2′_^II^), 56.7 (C_1′_), 59.2 (d, ^1^*J*_CP_ = 156.3, C_1_), 72.0 (d, ^2^*J*_CP_ = 7.7, CHO^I^), 72.4 (d, ^2^*J*_CP_ = 7.3, CHO^II^), 123.2 (d, *J*_CP_ = 1.7, C_4_), 124.5 (d, ^3^*J*_CP_ = 2.6, C_7_), 128.6 (d, *J*_CP_ = 2.2, C_5_), 131.1 (d, *J*_CP_ = 2.6, C_6_), 133.8 (d, ^3^*J*_CP_ = 4.1, C_3a_), 139.0 (d, ^2^*J*_CP_ = 5.6, C_7a_), 169.0 (d, ^3^*J*_CP_ = 3.7, C_3_); [M + H]^+^_found_ = 380.1990, C_20_H_31_NO_4_P requires 380.1985.

#### 3.2.9. Dibutyl (2-Cyclohexyl-3-oxo-2,3-dihydro-1*H*-isoindol-1-yl)phosphonate (**8d**)

Yield: 80% (0.33 g), colourless oil; ^31^P (CDCl_3_) δ 20.6; ^1^H NMR (CDCl_3_) δ 0.85 (t, *J*_HH_ = 7.3, 3H, C*H*_3_(CH_2_)_3_O^I^), 0.86 (t, *J*_HH_ = 7.3, 3H, C*H*_3_(CH_2_)_3_O^II^), 1.17–1.36 (m, 6H, C*H*_2_(CH_2_)_2_O, *^c^*HexH), 1.39–1.49 (m, 2H, C*H*_2_CH_2_O^I^), 1.49–1.60 (m, 2H, C*H*_2_CH_2_O^II^), 1.62–1.98 (m, 6H, *^c^*HexH), 2.15–2.33 (m, 1H, *^c^*HexH), 2.35–2.54 (m, 1H, *^c^*HexH), 3.64–3.82 (m, 2H, CH_A_, CH_2_O^I^, C_1′_H), 3.82–3.95 (m, 1H, CH_B_, CH_2_O^I^), 3.95–4.12 (m, 2H, CH_2_O^II^), 4.85 (d, *J*_HP_ = 13.2, 1H, C_1_H), 7.43–7.58 (m, 2H, C_5_H, C_6_H), 7.73 (d, *J*_HH_ = 7.5, 1H, C_7_H), 7.82 (d, *J*_HH_ = 7.2, 1H, C_4_H); ^13^C NMR (CDCl_3_) δ 13.4 (*C*H_3_(CH_2_)_3_O^I^), 13.5 (*C*H_3_(CH_2_)_3_O^II^), 18.5 (*C*H_2_(CH_2_)_2_O^I^), 18.6 (*C*H_2_(CH_2_)_2_O^II^), 25.3 (C_4′_), 26.1 (C_3′_^I^), 26.3 (C_3′_^II^), 29.3 (C_2′_^I^), 29.6 (C_2′_^II^), 32.36 (d, ^3^*J*_CP_ = 5.6, *C*H_2_CH_2_O^I^), 32.40 (d, ^3^*J*_CP_ = 6.0, *C*H_2_CH_2_O^II^), 56.7 (C_1′_), 58.6 (d, ^1^*J*_CP_ = 155.6, C_1_), 66.9 (d, ^2^*J*_CP_ = 7.6, CH_2_O^I^), 67.1 (d, ^2^*J*_CP_ = 7.3, CH_2_O^II^), 123.3 (d, *J*_CP_ = 1.4, C_4_), 124.4 (d, ^3^*J*_CP_ = 2.6, C_7_), 128.7 (d, *J*_CP_ = 2.2, C_5_), 131.1 (d, *J*_CP_ = 2.6, C_6_), 133.6 (d, ^3^*J*_CP_ = 4.0, C_3a_), 138.9 (d, ^2^*J*_CP_ = 5.8, C_7a_), 169.0 (d, ^3^*J*_CP_ = 3.6, C_3_); [M + H]^+^_found_ = 408.2298, C_22_H_35_NO_4_P requires 408.2298.

#### 3.2.10. Dibenzyl (2-Cyclohexyl-3-oxo-2,3-dihydro-1*H*-isoindol-1-yl)phosphonate (**8e**)

Yield: 70% (0.33 g), white crystals; Mp: 97–98 °C; ^31^P (CDCl_3_) δ 19.5; ^1^H NMR (CDCl_3_) δ 1.06–1.31 (m, 3H, *^c^*HexH), 1.51–1.62 (m, 1H, *^c^*HexH), 1.66–1.92 (m, 4H, *^c^*HexH), 2.10–2.23 (m, 1H, *^c^*HexH), 2.30–2.42 (m, 1H, *^c^*HexH), 3.67–3.77 (m, 1H, C_1′_H), 4.64–4.73 (m, 1H, CH_A_, CH_2_O^I^), 4.79–4.87 [4.84 (d, *J*_HP_ = 12.7, C_1_H) overlapped by the multiplet of CH_B_, CH_2_O^I^ total int. 2H], 4.87–5.02 (m, 2H, CH_2_O^II^), 7.14–7.21 (m, 4H, C_2′’_H), 7.27–7.33 (m, 6H, C_3,4′’_H), 7.45 (t, *J*_HH_ = 7.4, 1H, C_5_H), 7.50 (t, *J*_HH_ = 7.5, 1H, C_6_H), 7.69 (d, *J*_HH_ = 8.3, 1H, C_7_H), 7.76 (d, *J*_HH_ = 7.5, 1H, C_4_H); ^13^C NMR (CDCl_3_) δ 25.1 (C_4′_), 25.9 (C_3′_^I^), 26.1 (C_3′_^II^), 29.3 (C_2′_^I^), 29.6 (C_2′_^II^), 56.5 (C_1′_), 58.8 (d, ^1^*J*_CP_ = 155.4, C_1_), 68.7 (d, ^2^*J*_CP_ = 7.5, CH_2_O^I^), 68.8 (d, ^2^*J*_CP_ = 6.9, CH_2_O^II^), 123.4 (d, *J*_CP_ = 1.4, C_4_), 124.3 (d, ^3^*J*_CP_ = 2.4, C_7_), 128.0 (C_3′’_^I^), 128.2 (C_3′’_^II^), 128.58 (C_2′’_^I^), 128.61 (C_2′’_^II^), 128.62 (C_4′’_^I^), 128.66 (C_4′’_^II^), 128.74 (d, *J*_CP_ = 2.4, C_5_), 131.2 (d, *J*_CP_ = 2.5, C_6_), 133.5 (d, ^3^*J*_CP_ = 4.3, C_3a_), 135.4 (d, ^3^*J*_CP_ = 6.3, C_1′’_^I^), 135.5 (d, ^3^*J*_CP_ = 5.6, C_1′’_^II^), 138.5 (d, ^2^*J*_CP_ = 6.0, C_7a_), 168.9 (d, ^3^*J*_CP_ = 3.9, C_3_); [M + H]^+^_found_ = 476.1997, C_28_H_31_NO_4_P requires 476.1985.

#### 3.2.11. Diethyl (2-Benzyl-3-oxo-2,3-dihydro-1*H*-isoindol-1-yl)phosphonate (**9a**)

Yield: 90% (0.32 g), colourless oil; ^31^P (CDCl_3_) δ 18.3; ^31^P (CDCl_3_), δ[17] 17.7; [M + H]^+^_found_ = 360.1360, C_19_H_23_NO_4_P requires 360.1359.

#### 3.2.12. Dimethyl (2-Benzyl-3-oxo-2,3-dihydro-1*H*-isoindol-1-yl)phosphonate (**9b**)

Yield: 80% (0.27 g), light yellow crystals; Mp: 130–131 °C; Mp [13]: 130–132 °C; ^31^P (CDCl_3_) δ 21.8; δ[13] 21.6; [M + H]^+^_found_ = 332.1050, C_17_H_19_NO_4_P requires 332.1046.

#### 3.2.13. Diisopropyl (2-Benzyl-3-oxo-2,3-dihydro-1*H*-isoindol-1-yl)phosphonate (**9c**)

Yield: 83% (0.32 g), colourless oil; ^31^P (CDCl_3_) δ 16.6; ^1^H NMR (CDCl_3_) δ 0.85 (d, *J*_HH_ = 6.2, 3H, C*H*_3_CH^I^), 1.16 (d, *J*_HH_ = 6.2, 3H, C*H*_3_CH^II^), 1.19 (d, *J*_HH_ = 6.1, 3H, C*H*_3_CH^III^), 1.23 (d, *J*_HH_ = 6.2, 3H, C*H*_3_CH^IV^), 4.28–4.44 (m, 1H, CHO^I^), 4.50–4.74 [4.56 (d, *J*_HP_ = 15.0, CH_A_, CH_2_N), 4.57 (d, *J*_HP_ = 12.5, C_1_H) overlapped by the multiplet of CHO^II^ total int. 3H], 5.48 (d, *J*_HP_ = 14.9, 1H, CH_B_, CH_2_N), 7.12–7.29 (m, 5H, C_2_-_4′_H), 7.39–7.55 (m, 2H, C_5_H, C_6_H), 7.64 (d, *J*_HH_ = 7.1, 1H, C_7_H), 7.84 (d, *J*_HH_ = 6.8, 1H, C_4_H); ^13^C NMR (CDCl_3_) δ 23.3 (d, ^3^*J*_CP_ = 5.4, *C*H_3_CH^I^), 23.9 (d, ^3^*J*_CP_ = 5.1, *C*H_3_CH^II^), 24.06 (d, ^3^*J*_CP_ = 5.8, *C*H_3_CH^III^), 24.11 (d, ^3^*J*_CP_ = 5.4, *C*H_3_CH^IV^), 44.9 (CH_2_N), 56.9 (d, ^1^*J*_CP_ = 156.5, C_1_), 72.3 (d, ^2^*J*_CP_ = 7.4, CHO^I^), 72.6 (d, ^2^*J*_CP_ = 7.2, CHO^II^), 123.9 (d, *J*_CP_ = 1.6, C_4_), 124.5 (d, ^3^*J*_CP_ = 2.5, C_7_), 127.6 (C_4′_), 128.4 (C_3′_), 128.68 (C_2′_), 128.70 (d, *J*_CP_ = 2.6, C_5_), 131.6 (d, *J*_CP_ = 2.6, C_6_), 132.2 (d, ^3^*J*_CP_ = 4.3, C_3a_), 136.9 (C_1′_), 139.0 (d, ^2^*J*_CP_ = 5.8, C_7a_), 168.9 (d, ^3^*J*_CP_ = 3.7, C_3_); [M + H]^+^_found_ = 388.1667, C_21_H_27_NO_4_P requires 388.1672.

#### 3.2.14. Dibutyl (2-Benzyl-3-oxo-2,3-dihydro-1*H*-isoindol-1-yl)phosphonate (**9d**)

Yield: 85% (0.35 g), colourless oil; ^31^P (CDCl_3_) δ 18.3; ^1^H NMR (CDCl_3_) δ 0.83 (t, *J*_HH_ = 7.4, 3H, (C*H*_3_CH_2_)_3_O^I^), 0.89 (t, *J*_HH_ = 7.4, 3H, C*H*_3_(CH_2_)_3_O^II^), 1.15–1.36 (m, 4H, C*H*_2_(CH_2_)_2_O), 1.36–1.50 (m, 2H, C*H*_2_CH_2_O^I^), 1.52–1.64 (m, 2H, C*H*_2_CH_2_O^II^), 3.67–3.79 (m, 1H, CH_A_, CH_2_O^I^), 3.84–3.79 (m, 1H, CH_B_, CH_2_O^I^), 3.98–4.10 (m, 2H, CH_2_O^II^), 4.6 (d, *J*_HP_ = 14.9, 1H, CH_A_, CH_2_N), 4.68 (d, *J*_HP_ = 13.3, 1H, C_1_H), 5.57 (d, *J*_HP_ = 14.8, 1H, CH_B_, CH_2_N), 7.21–7.34 (m, 5H, C_2_-_4′_H), 7.47–7.60 (m, 2H, C_5_H, C_6_H), 7.70 (d, *J*_HH_ = 7.0, 1H, C_7_H), 7.92 (d, *J*_HH_ = 6.7, 1H, C_4_H); ^13^C NMR (CDCl_3_) 13.4 (*C*H_3_(CH_2_)_3_O^I^), 13.5 (*C*H_3_(CH_2_)_3_O^II^), 18.5 (*C*H_2_(CH_2_)_2_O^I^), 18.6 (*C*H_2_(CH_2_)_2_O^II^), 32.3 (d, ^3^*J*_CP_ = 5.6, *C*H_2_CH_2_O^I^), 32.5 (d, ^3^*J*_CP_ = 5.6, *C*H_2_CH_2_O^II^), 45.0 (CH_2_N), 56.4 (d, ^1^*J*_CP_ = 155.5, C_1_), 66.9 (d, ^2^*J*_CP_ = 7.4, CH_2_O^I^), 67.1 (d, ^2^*J*_CP_ = 7.3, CH_2_O^II^), 124.0 (d, *J*_CP_ = 1.7, C_4_), 124.5 (d, ^3^*J*_CP_ = 2.6, C_7_), 127.6 (C_4′_), 128.3 (C_3′_), 128.7 (C_2′_), 128.8 (d, *J*_CP_ = 2.4, C_5_), 131.6 (d, *J*_CP_ = 2.5, C_6_), 132.0 (d, ^3^*J*_CP_ = 4.3, C_3a_), 136.8 (C_1′_), 138.8 (d, ^2^*J*_CP_ = 5.7, C_7a_), 168.8 (d, ^3^*J*_CP_ = 3.6, C_3_); [M + H]^+^_found_ = 416.1979, C_23_H_31_NO_4_P requires 416.1985.

#### 3.2.15. Dibenzyl (2-Benzyl-3-oxo-2,3-dihydro-1*H*-isoindol-1-yl)phosphonate (**9e**)

Yield: 84% (0.33 g), colourless oil; ^31^P (CDCl_3_) δ 19.2; ^1^H NMR (CDCl_3_) δ 4.49 (d, *J*_HP_ = 14.9, 1H, CH_A_, CH_2_N), 4.61–4.74 [4.65 (d, *J*_HP_ = 13.0, C_1_H) overlapped by the multiplet of CH_A_, CH_2_O^I^ total int. 2H], 4.79–4.86 (m, 1H, CH_B_, CH_2_O^I^), 4.92–5.02 (m, 2H, CH_2_O^II^), 5.48 (d, *J*_HP_ = 14.9, 1H, CH_B_, CH_2_N), 7.10–7.18 (m, 4H, ArH), 7.18–7.25 (m, 5H, ArH), 7.27–7.31 (m, 3H, ArH), 7.31–7.39 (m, 3H, ArH), 7.45–7.55 (m, 2H, C_5_H, C_6_H), 7.64 (d, *J*_HH_ = 6.9, 1H, C_7_H), 7.87 (d, *J*_HH_ = 5.8, 1H, C_4_H); ^13^C NMR (CDCl_3_) 45.0 (CH_2_N), 56.6 (d, ^1^*J*_CP_ = 155.5, C_1_), 68.6 (d, ^2^*J*_CP_ = 7.3, CH_2_O^I^), 68.9 (d, ^2^*J*_CP_ = 6.9, CH_2_O^II^), 124.1 (d, *J*_CP_ = 1.7, C_4_), 124.4 (d, ^3^*J*_CP_ = 2.6, C_7_), 127.6 (C_4′_), 128.1 (C_3′’_^I^), 128.3 (C_3′’_^II^), 128.4 (C_3′_), 128.65 (C_2′’_^I^), 128.66 (C_2′_), 128.67 (C_2′’_^II^), 128.71 (C_4′’_^I^), 128.8 (C_4′’_^II^), 128.9 (d, *J*_CP_ = 2.3, C_5_), 131.7 (d, *J*_CP_ = 2.6, C_6_), 132.0 (d, ^3^*J*_CP_ = 4.1, C_3a_), 135.36 (d, ^3^*J*_CP_ = 5.1, C_1′’_^I^), 135.40 (d, ^3^*J*_CP_ = 5.5, C_1′’_^II^), 136.7 (C_1′_), 138.4 (d, ^2^*J*_CP_ = 5.8, C_7a_), 168.7 (d, ^3^*J*_CP_ = 3.7, C_3_); [M + H]^+^_found_ = 484.1679, C_29_H_27_NO_4_P requires 484.1672.

#### 3.2.16. Ethyl (2-Butyl-3-oxo-2,3-dihydro-1*H*-isoindol-1-yl)(phenyl)phosphinate (**10**)

Yield: 78% (0.28 g), colourless oil; [M + H]^+^_found_ = 358.1558, C_20_H_25_NO_3_P requires 358.1566; Product **10** formed as a mixture of two isomers (**A** and **B**) in a proportion of 47:53 based on ^31^P NMR. The two series of signals could be separated in the NMR spectra: **Isomer A**: ^31^P (CDCl_3_) δ 35.4; ^1^H NMR (CDCl_3_) δ 0.94 (t, *J*_HH_ = 7.4, 3H, C*H*_3_(CH_2_)_3_N), 1.23–1.38 (m, 2H, C*H*_2_(CH_2_)_2_N), 1.43 (t, *J*_HH_ = 7.0, 3H, C*H*_3_CH_2_O), 1.54–1.80 (m, 2H, C*H*_2_CH_2_N), 3.66–3.78 (m, 1H, CH_A_, CH_2_N), 4.19–4.39 (m, 3H, CH_B_, CH_2_N, CH_2_O), 5.05 (d, *J*_HP_ = 16.7, 1H, C_1_H), 7.12–7.67 (m, 8H, ArH, C_5–7_H), 7.75 (d, *J*_HH_ = 7.8, 1H, C_4_H); ^13^C NMR (CDCl_3_) δ 13.7 (*C*H_3_(CH_2_)_3_N), 16.50 (d, ^3^*J*_CP_ = 6.1, *C*H_3_CH_2_O), 20.0 (*C*H_2_(CH_2_)_2_N), 30.02 (*C*H_2_CH_2_N), 41.5 (CH_2_N), 60.1 (d, ^1^*J*_CP_ = 107.9, C_1_), 61.9 (d, ^2^*J*_CP_ = 6.7, CH_2_O), 123.4 (d, *J*_CP_ = 1.6, C_4_), 123.7 (d, ^3^*J*_CP_ = 1.7, C_7_), 124.45 (d, ^3^*J*_CP_ = 23.1, C_3′_), 125.5 (d, ^1^*J*_CP_ = 127.3, C_1′_), 128.21 (d, ^2^*J*_CP_ = 12.7, C_2′_), 128.67 (d, *J*_CP_ = 2.0, C_5_), 131.1 (d, *J*_CP_ = 2.3, C_6_), 132.1 (d, *J*_CP_ = 9.4, C_4′_), 133.04 (d, ^3^*J*_CP_ = 3.0, C_3a_), 138.7 (d, ^2^*J*_CP_ = 4.1, C_7a_), 168.3 (d, ^3^*J*_CP_ = 2.6, C_3_); **Isomer B**: ^31^P (CDCl_3_) δ 39.2; ^1^H NMR (CDCl_3_) δ 0.95 (t, *J*_HH_ = 7.4, 3H, C*H*_3_(CH_2_)_3_N), 1.23–1.38 (m, 2H, C*H*_2_(CH_2_)_2_N), 1.45 (t, *J*_HH_ = 7.0, 3H, C*H*_3_CH_2_O), 1.54–1.80 (m, 2H, C*H*_2_CH_2_N), 3.51–3.66 (m, 1H, CH_A_, CH_2_N), 3.98–4.19 (m, 3H, CH_B_, CH_2_N, CH_2_O), 5.05 (d, *J*_HP_ = 16.7, 1H, C_1_H), 7.12–7.67 (m, 8H, ArH, C_5–7_H), 7.86 (d, *J*_HH_ = 7.7, 1H, C_4_H); ^13^C NMR (CDCl_3_) δ 13.7 (*C*H_3_(CH_2_)_3_N), 16.54 (d, ^3^*J*_CP_ = 6.1, *C*H_3_CH_2_O), 20.0 (*C*H_2_(CH_2_)_2_N), 29.98 (*C*H_2_CH_2_N), 41.4 (CH_2_N), 59.4 (d, ^1^*J*_CP_ = 104.5, C_1_), 62.2 (d, ^2^*J*_CP_ = 7.1, CH_2_O), 123.4 (d, *J*_CP_ = 1.6, C_4_), 123.7 (d, ^3^*J*_CP_ = 1.7, C_7_), 124.42 (d, ^3^*J*_CP_ = 22.8, C_3′_), 125.1 (d, ^1^*J*_CP_ = 128.4, C_1′_), 128.25 (d, ^2^*J*_CP_ = 12.8, C_2′_), 128.60 (d, *J*_CP_ = 2.5, C_5_), 131.3 (d, *J*_CP_ = 2.6, C_6_), 131.9 (d, *J*_CP_ = 9.3, C_4′_), 132.98 (d, ^3^*J*_CP_ = 3.3, C_3a_), 138.1 (d, ^2^*J*_CP_ = 1.8, C_7a_), 168.7 (d, ^3^*J*_CP_ = 2.1, C_3_).

### 3.3. Procedure for the Real Time Monitoring of the Condensation of 2-Formylbenzoic Acid, Butylamine and Diethyl Phosphite by In Situ FT-IR Spectroscopy

To 30 mL of ethanol, the starting materials were mixed at room temperature (25 °C) in ten-minute intervals in the following order: 30 mmol (4.5 g) of 2-formylbenzoic acid, 30 mmol (3.9 mL) of diethyl phosphite and 36 mmol (3.6 mL) of butylamine. After the additions, the mixture was heated gradually from 25 °C to 60 °C in an oil bath. Then the temperature was held for an additional 2.5 h.

### 3.4. Procedure for the Continuous Flow Synthesis of Diethyl (2-butyl-3-oxo-2,3-dihydro-1H-isoindol-1-yl)phosphonate *(**7a**)*

Before each experiment, two solutions (A and B) were prepared. For solution A, 41.8 mmol of 2-formylbenzoic acid was dissolved in ethanol in a 50 cm^3^ volumetric flask, resulting in the aldehyde concentration of 0.84 M. For solution B, amines (50.2 mmol of butylamine (5.0 mL) or 62.7 mmol of butylamine (6.2 mL), 62.7 mmol of cyclohexylamine (7.2 mL) or 62.7 mmol of benzylamine (6.8 mL)) and 41.8 mmol of diethyl phosphite (5.4 mL) or 62.7 mmol of diethyl phosphite (8.1 mL) were dissolved in ethanol in a 50 cm^3^ volumetric flask, resulting in the amine concentration of 1.01 M or 1.26 M and the dialkyl phosphite concentration of 0.84 M or 1.26 M, respectively. The two solutions (A and B) were pumped separately. The system was flushed with 15 mL of solution A and 15 mL of solution B with a summa flow rate of 10 mL/min (A:B = 1:1) at 25 °C and 17 bar. Next, under the same pressure, the flow rate was set to the desired value (can be seen in Table 2) and the vessel was irradiated with a power of 10–15 W for 2–3 min, until the temperature reached the desired value, then the power was controlled automatically by the software of the MW reactor. The operation was regarded steady state on the basis of the results of the GC measurements. The desired diethyl (2-butyl-3-oxo-2,3-dihydro-1*H*-isoindol-1-yl)phosphonate (**7a**), diethyl (2-cyclohexyl-3-oxo-2,3-dihydro-1*H*-isoindol-1-yl)phosphonate (**8a**) or diethyl (2-benzyl-3-oxo-2,3-dihydro-1*H*-isoindol-1-yl)phosphonate (**9a**) were obtained by column chromatography using silica gel as the absorbent and dichloromethane:methanol (97:3) as the eluent. Yields were calculated on the basis of the weights obtained after separation and evaporation taking into consideration the quantity of the limiting starting materials fed in during a given time. After a 3 h flow operation in steady state, 6.9 g of diethyl (2-butyl-3-oxo-2,3-dihydro-1*H*-isoindol-1-yl)phosphonate (**7a**), 4.1 g of diethyl (2-cyclohexyl-3-oxo-2,3-dihydro-1*H*-isoindol-1-yl)phosphonate (**8a**) or 5.3 g of diethyl (2-benzyl-3-oxo-2,3-dihydro-1*H*-isoindol-1-yl)phosphonate (**9a**) were obtained.

## 4. Conclusions

In conclusion, we have developed a facile, efficient, catalyst-free method for the batch and continuous flow preparation of isoindolin-1-one-3-phosphonates containing alkyl substituents on the nitrogen atom. The novel batch approach enabled the solvent-free synthesis of the target compounds in high yields (70–94%) at low temperature (60 °C) under short reaction times (10–30 min). By the in situ FT-IR study on the condensation of 2-formylbenzoic acid, butylamine and diethyl phosphite, the reaction was followed in “real time” and the time-dependent concentration profiles of the reaction components were determined. The continuous flow MW-assisted method developed for the condensation of 2-formylbenzoic acid, aliphatic primary amines and diethyl phosphite are suitable for the synthesis of the corresponding isoindolin-1-one-3-phosphonates in a “few g” scale. Altogether 16 derivatives were isolated and characterized. Except two, all of them are new compounds.

## Figures and Tables

**Table 1:**
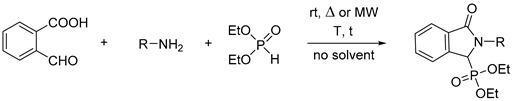
Optimization of the condensation of 2-formylbenzoic acid, aliphatic primary amines and diethyl phosphite in a batch microwave reactor.

Entry	Mode of Heating	R	Amine (equiv)	DEP (equiv)	T (°C)	t (min)	Conversion ^a^ (%)	Yield ^b^ (%)
1	-	Bu	1	1	25	180^c^	77	-
2	MW	Bu	1	1	40	10	61	-
3	MW	Bu	1	1	60	10	85	-
4	MW	Bu	1	1	80	10	87	-
5	MW	Bu	1	1	60	20	90	-
6	MW	Bu	1	1	60	30	91	-
7	MW	Bu	1	1.1	60	10	87	-
8	MW	Bu	1	1.2	60	10	89	-
9	MW	Bu	1.1	1	60	10	92	-
10	MW	Bu	1.2	1	60	10	100	94 (**7a**)
11	-	Bu	1.2	1	60	10	90	-
12	MW	*^c^*Hex	1.2	1	60	10	82	-
13	MW	*^c^*Hex	1.2	1	60	20	93	-
14	MW	*^c^*Hex	1.2	1	60	30	100	84 (**8a**)
15	-	*^c^*Hex	1.2	1	60	30	84	-
16	MW	Bn	1.2	1	60	10	89	-
17	MW	Bn	1.2	1	60	20	100	90 (**9a**)
18	-	Bn	1.2	1	60	20	88	-

^a^ On the basis of GC; ^b^ After column chromatography; ^c^ No change after longer reaction time.

**Table 2:**
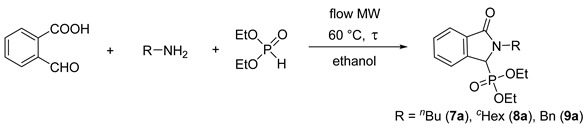
Optimization of the condensation of 2-formylbenzoic acid, aliphatic primary amines and diethyl phosphite in a continuous flow MW reactor.

Entry	R	Amine (equiv)	DEP (equiv)	Flow Rate (mL/min)	τ (min)	Conversion ^a^ (%)	Yield ^b^ (%)
1	Bu	1.2	1	0.70	10	43	-
2	Bu	1.2	1	0.35	20	52	-
3	Bu	1.2	1	0.25	30	56	-
4	Bu	1.5	1	0.35	20	70	-
5	Bu	2.0	1	0.35	20	73	-
6	Bu	1.5	1.2	0.35	20	81	-
7	Bu	1.5	1.5	0.35	20	95	-
8	Bu	1.5	1.5	0.25	30	100	95% (**7a**)
9	*^c^*Hex	1.5	1.5	0.15	45	100	86% (**8a**)
10	Bn	1.5	1.5	0.18	40	100	91% (**9a**)

^a^ On the basis of GC; ^b^ Isolated yield.

## Supplementary Materials

Supplementary materials are available online.
