# Supplementary material for: Study on the Microwave-Assisted Batch and Continuous Flow Synthesis of N-Alkyl-Isoindolin-1-One-3-Phosphonates by a Special Kabachnik–Fields Condensation
Source: Molecules. 2020 Jul 21;25(14):3307. doi: 10.3390/molecules25143307 (PMC7397064; doi:10.3390/molecules25143307)
Supplement: Supplementary file 1 [file molecules-25-03307-s001.pdf]

Supplementary Materials for

Study on the Microwave-Assisted Batch and Continuous Flow Synthesis of *N*-Alkyl-Isoindolin-1-One-3-Phosphonates by a Special Kabachnik-Fields Condensation

Ádám Tajti [1], Nóra Tóth [1], Bettina Rávai [1], István Csontos [1], Pál Tamás Szabó [2] and Erika Bálint [1,*]

[1] Department of Organic Chemistry and Technology, Budapest University of Technology and Economics, 1521 Budapest, Hungary; tajti.adam@mail.bme.hu (Á.T.); toth.nora@mail.bme.hu (N.T.); betti.ravai08@gmail.com (B.R.); icsontos@mail.bme.hu (I.C.)

[2] MS Metabolomics Laboratory, Instrumentation Center, Research Centre for Natural Sciences, Hungarian Academy of Sciences, Magyar tudósok krt. 2., H-1117 Budapest, Hungary; szabo.pal@ttk.mta.hu

* Correspondence: ebalint@mail.bme.hu; Tel.: +36-1-463-3653

Table of contents

Figures of the continuous flow system Figure S2

^{31}P NMR, ^{1}H NMR and ^{13}C NMR spectra

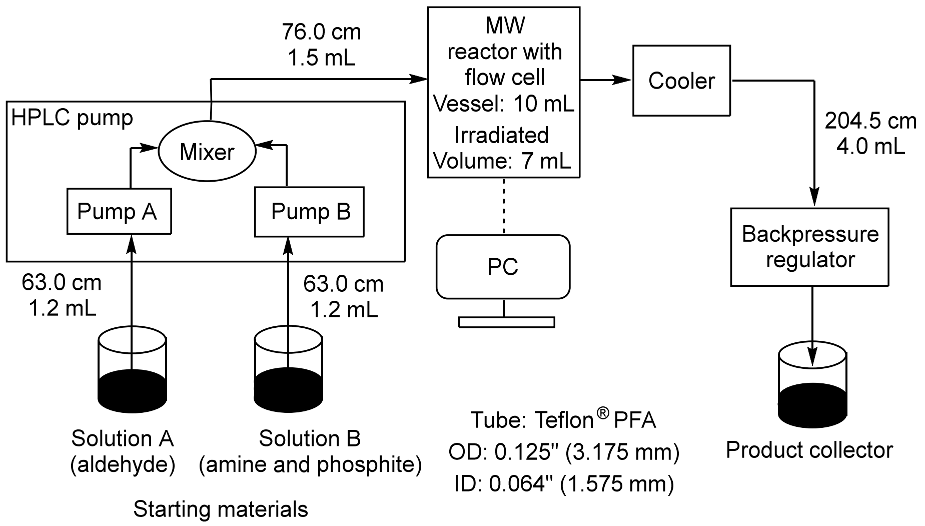

Figure S1. Design parameters of the continuous flow system.

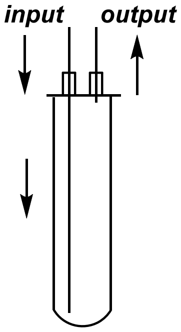

Figure S2. Sketch of the continuous flow cell.

^{31}P NMR, ^{1}H NMR and ^{13}C NMR spectra

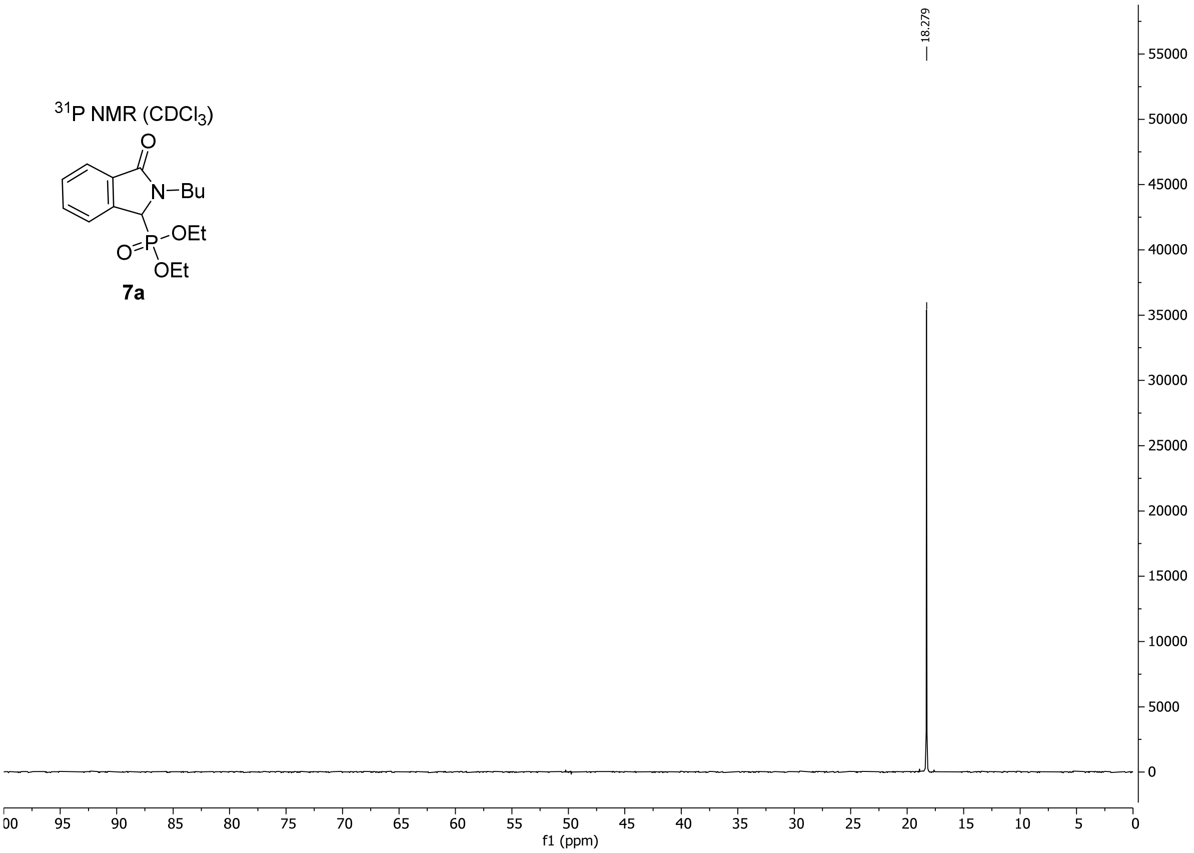

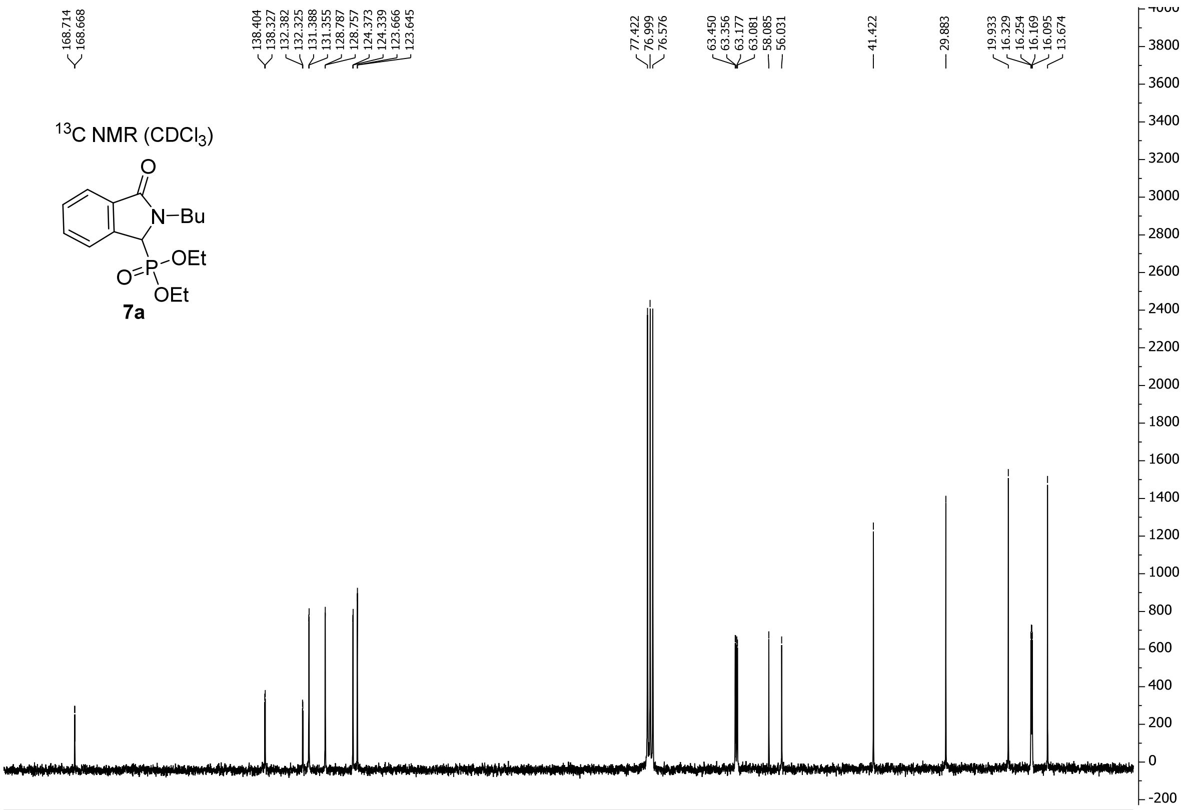

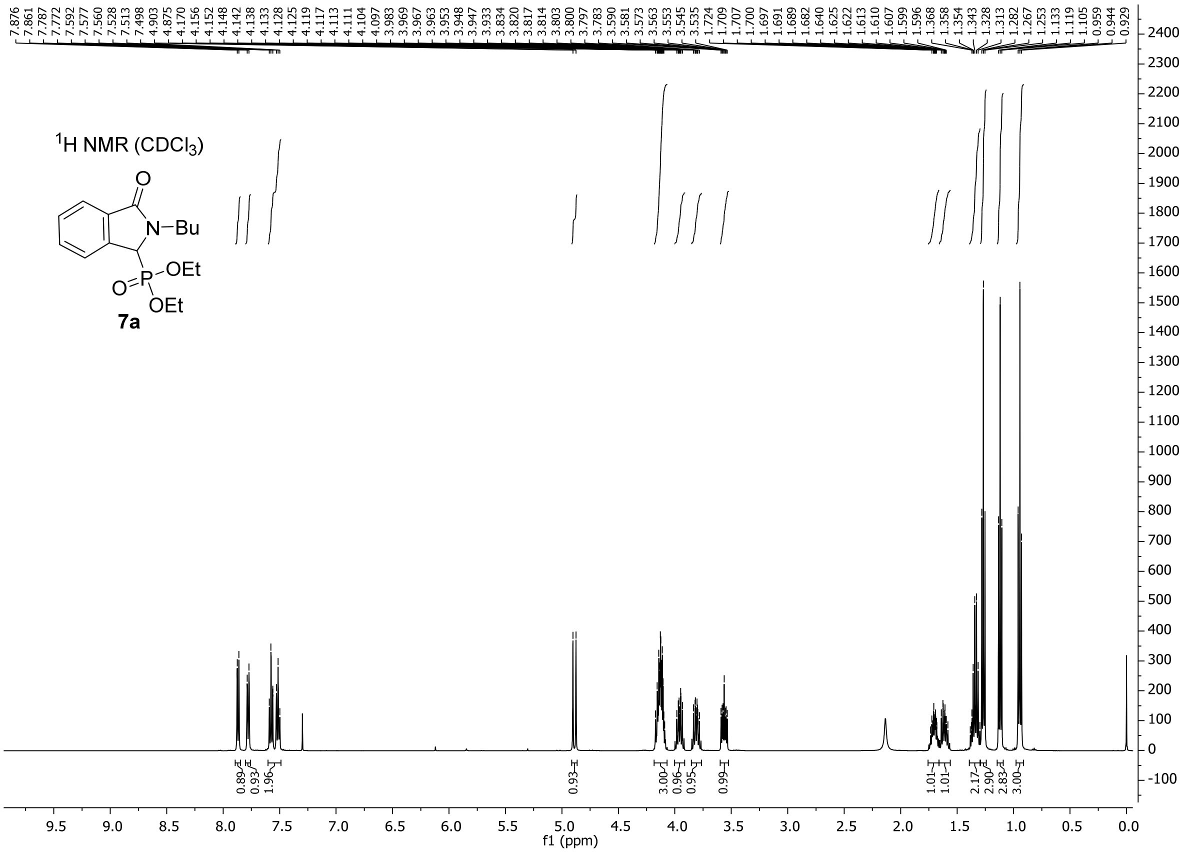
1H NMR (CDCl3)
O
N−Bu
O=P
OEt
OEt
7a
7.876 7.861 7.787 7.772 7.592 7.577 7.560 7.528 7.513 7.498 4.903 4.875 4.170 4.156 4.152 4.148 4.142 4.138 4.133 4.128 4.125 4.119 4.117 4.113 4.111 4.104 4.097 3.983 3.969 3.967 3.963 3.953 3.948 3.947 3.933 3.834 3.820 3.817 3.814 3.803 3.800 3.797 3.783 3.590 3.581 3.573 3.563 3.553 3.545 3.535 1.724 1.709 1.707 1.700 1.697 1.691 1.689 1.682 1.640 1.625 1.622 1.613 1.610 1.607 1.599 1.596 1.368 1.358 1.354 1.343 1.328 1.313 1.282 1.267 1.253 1.133 1.119 1.105 0.959 0.944 0.929
0.89 0.93 1.96 0.93 3.00 0.96 0.95 0.99 1.01 1.01 2.17 2.90 2.83 3.00
f1 (ppm)

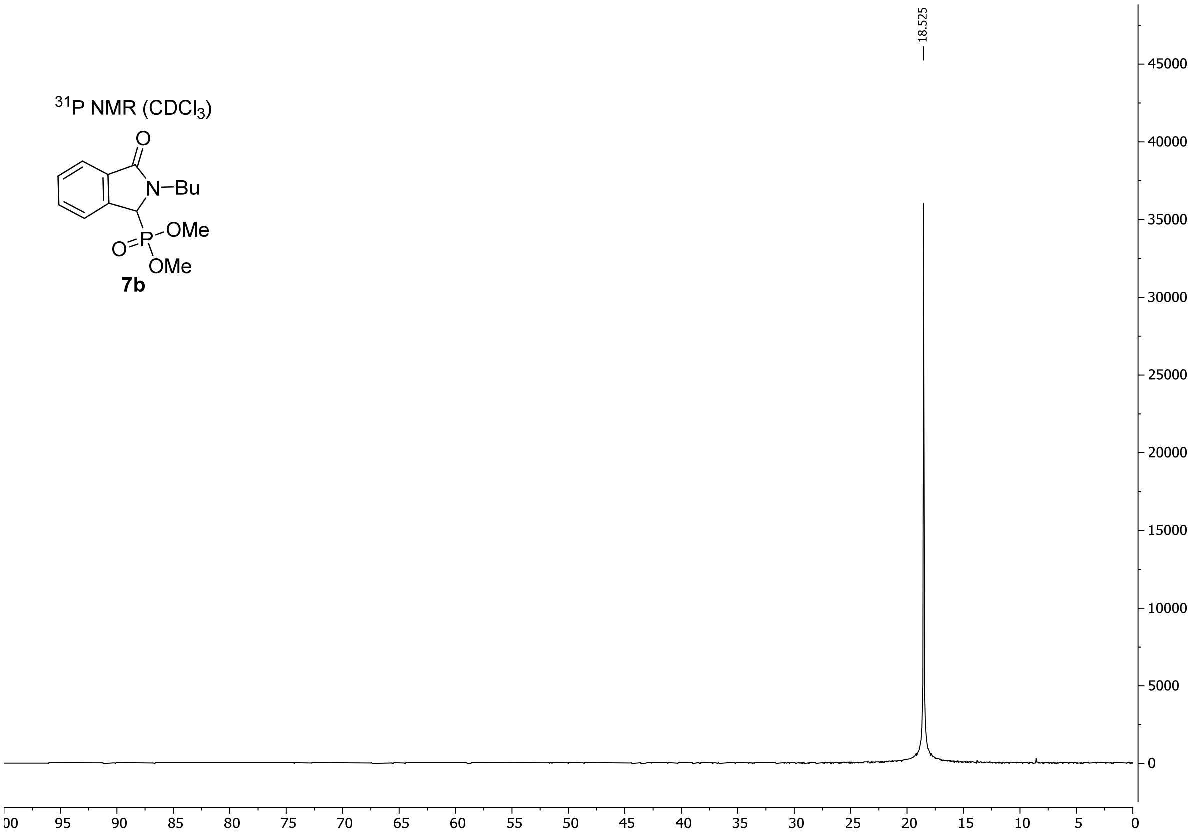
31P NMR (CDCl3)
O
N−Bu
O=P
OMe
OMe
7b
18.525
f1 (ppm)

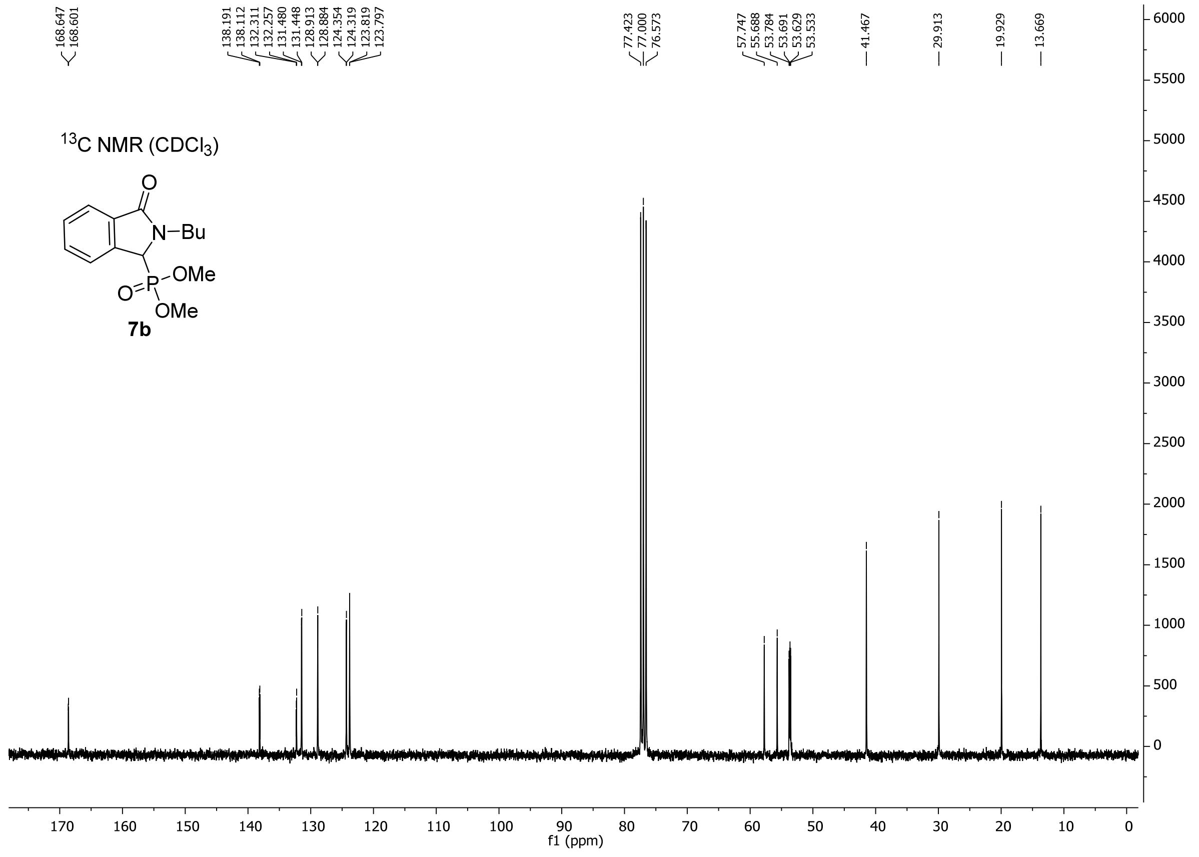
13C NMR (CDCl3)
N−Bu
OMe
OMe
7b
168.647
168.601
138.191
138.112
132.311
132.257
131.480
131.448
128.913
128.884
124.354
124.319
123.819
123.797
77.423
77.000
76.573
57.747
55.688
53.784
53.691
53.629
53.533
41.467
29.913
19.929
13.669
f1 (ppm)

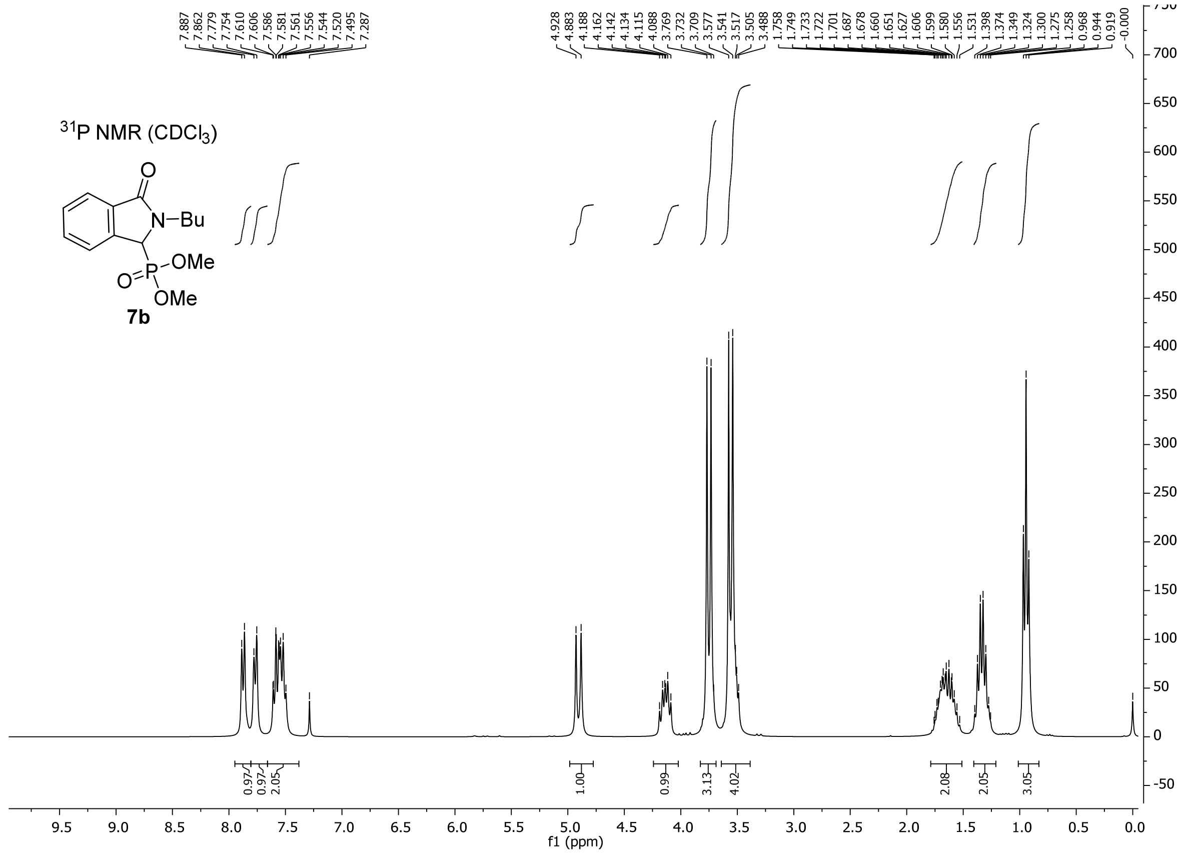
31P NMR (CDCl3)
N−Bu
OMe
OMe
7b
f1 (ppm)

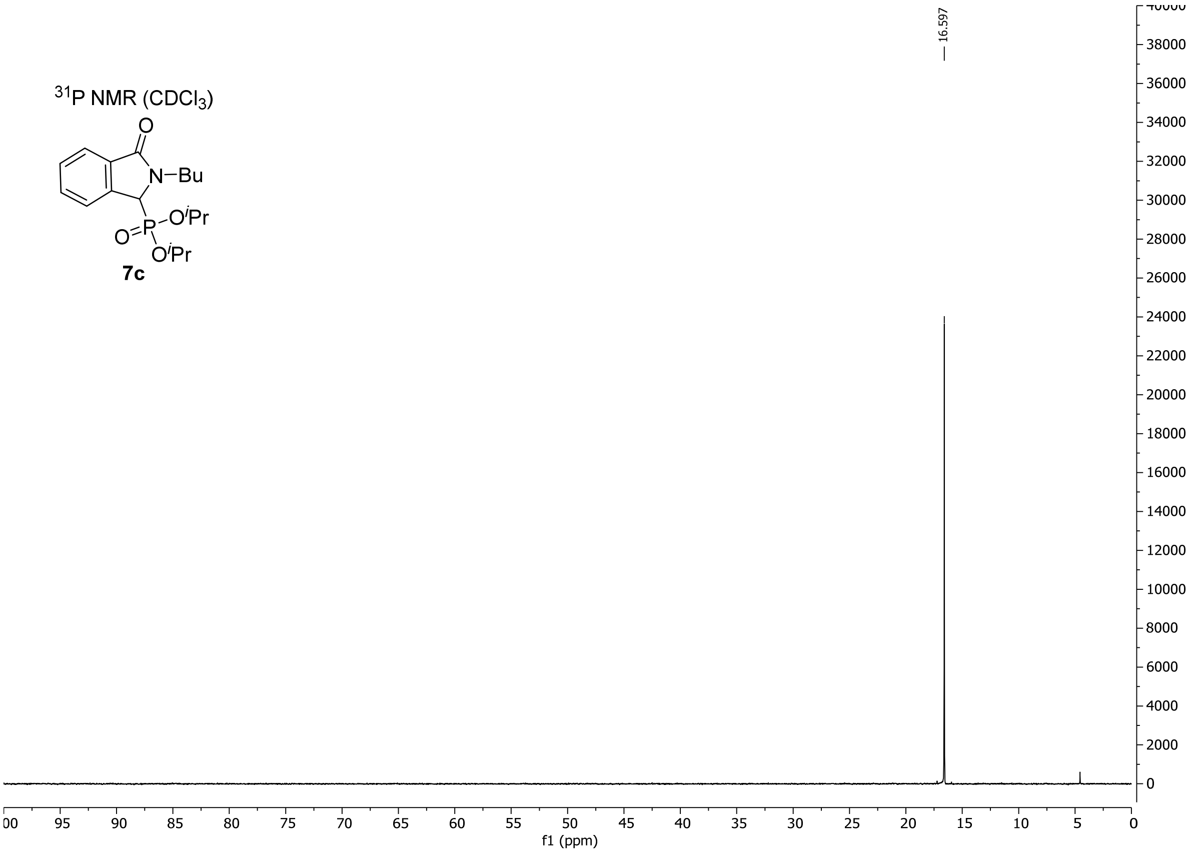

^{31}P NMR (CDCl$_3$)
7c
16.597
f1 (ppm)

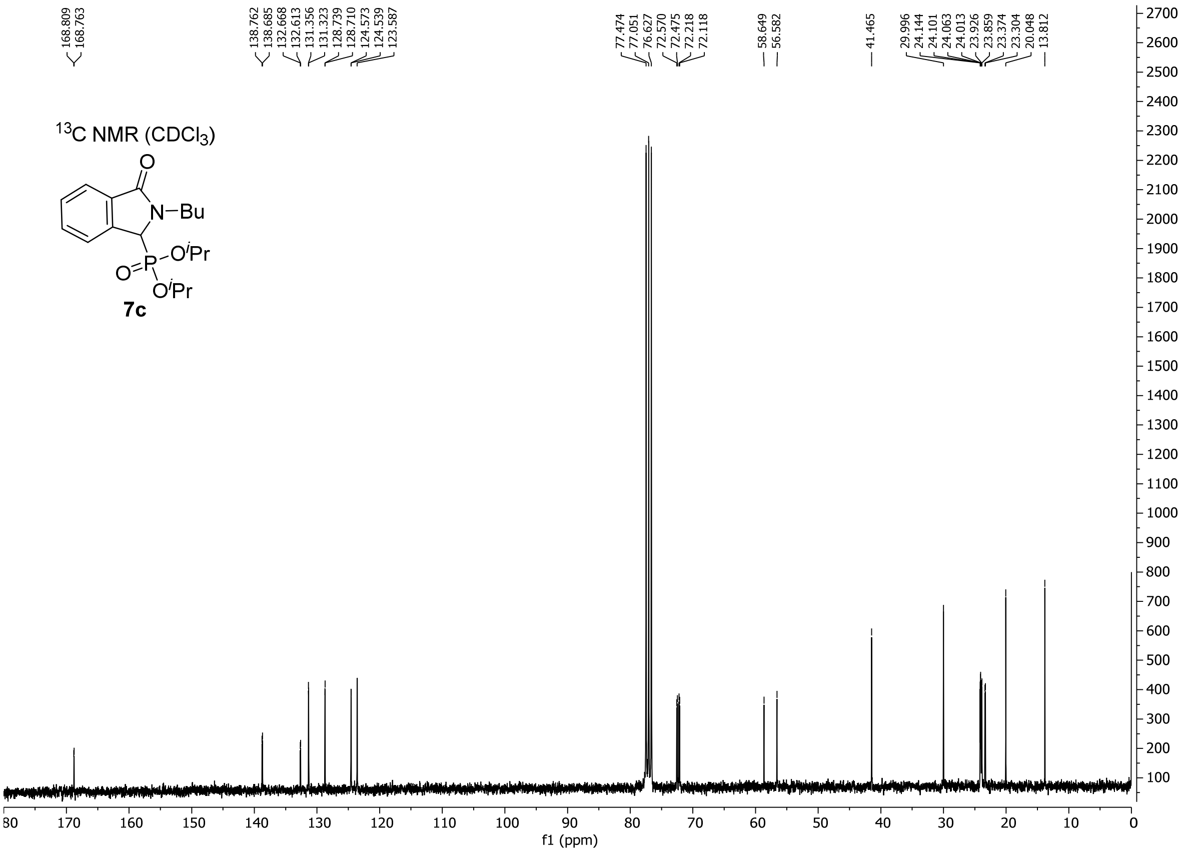

^{13}C NMR (CDCl$_3$)
7c
168.809
168.763
138.762
138.685
132.668
132.613
131.356
131.323
128.739
128.710
124.573
124.539
123.587
77.474
77.051
76.627
72.570
72.475
72.218
72.118
58.649
56.582
41.465
29.996
24.144
24.101
24.063
24.013
23.926
23.859
23.374
23.304
20.048
13.812
f1 (ppm)

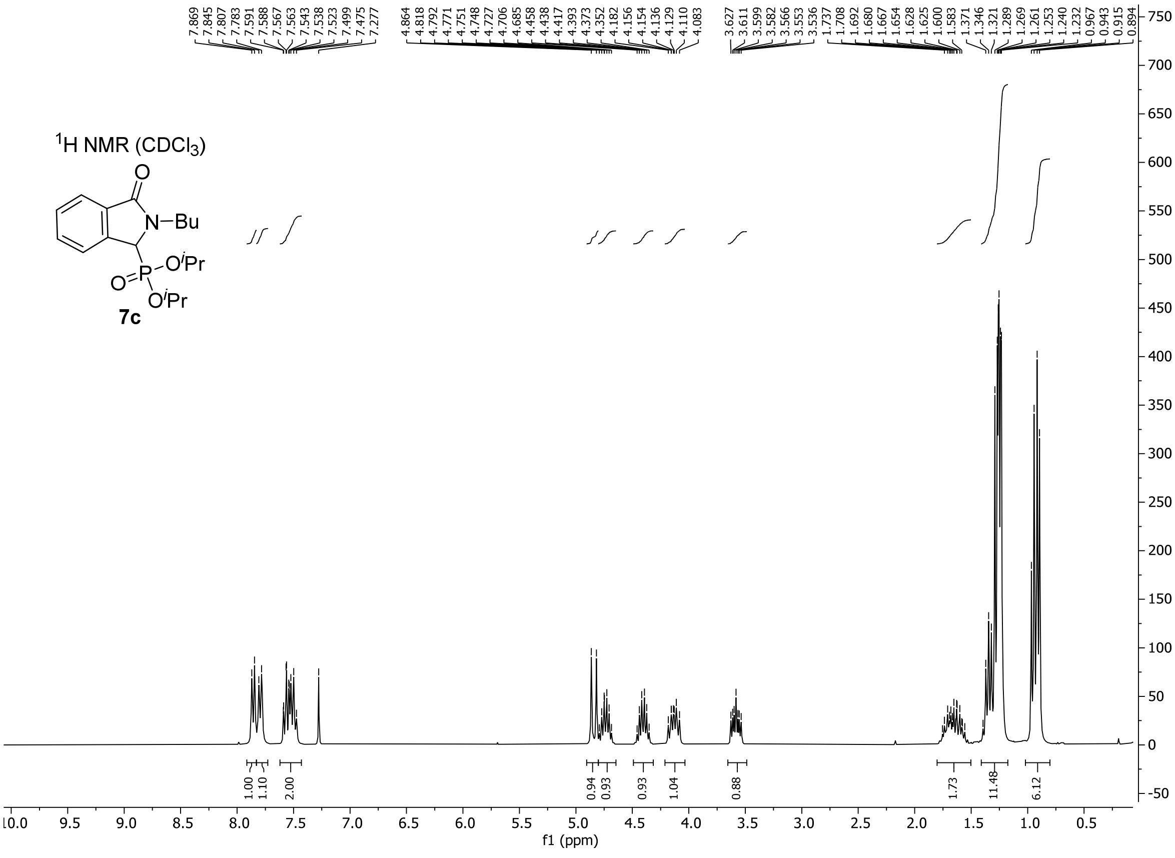
7.869 7.845 7.807 7.783 7.591 7.588 7.567 7.563 7.543 7.538 7.523 7.499 7.475 7.277
4.864 4.818 4.792 4.771 4.751 4.748 4.727 4.706 4.685 4.458 4.438 4.417 4.393 4.373 4.352 4.182 4.156 4.154 4.136 4.129 4.110 4.083
3.627 3.611 3.599 3.582 3.566 3.553 3.536 1.737 1.708 1.692 1.680 1.667 1.654 1.628 1.625 1.600 1.583 1.371 1.346 1.321 1.289 1.269 1.261 1.253 1.240 1.232 0.967 0.943 0.915 0.894
^{1}H NMR (CDCl$_3$)
O
N–Bu
O=P
O^{i}Pr
O^{i}Pr
7c
1.00 1.10 2.00 0.94 0.93 0.93 1.04 0.88 1.73 11.48 6.12
f1 (ppm)

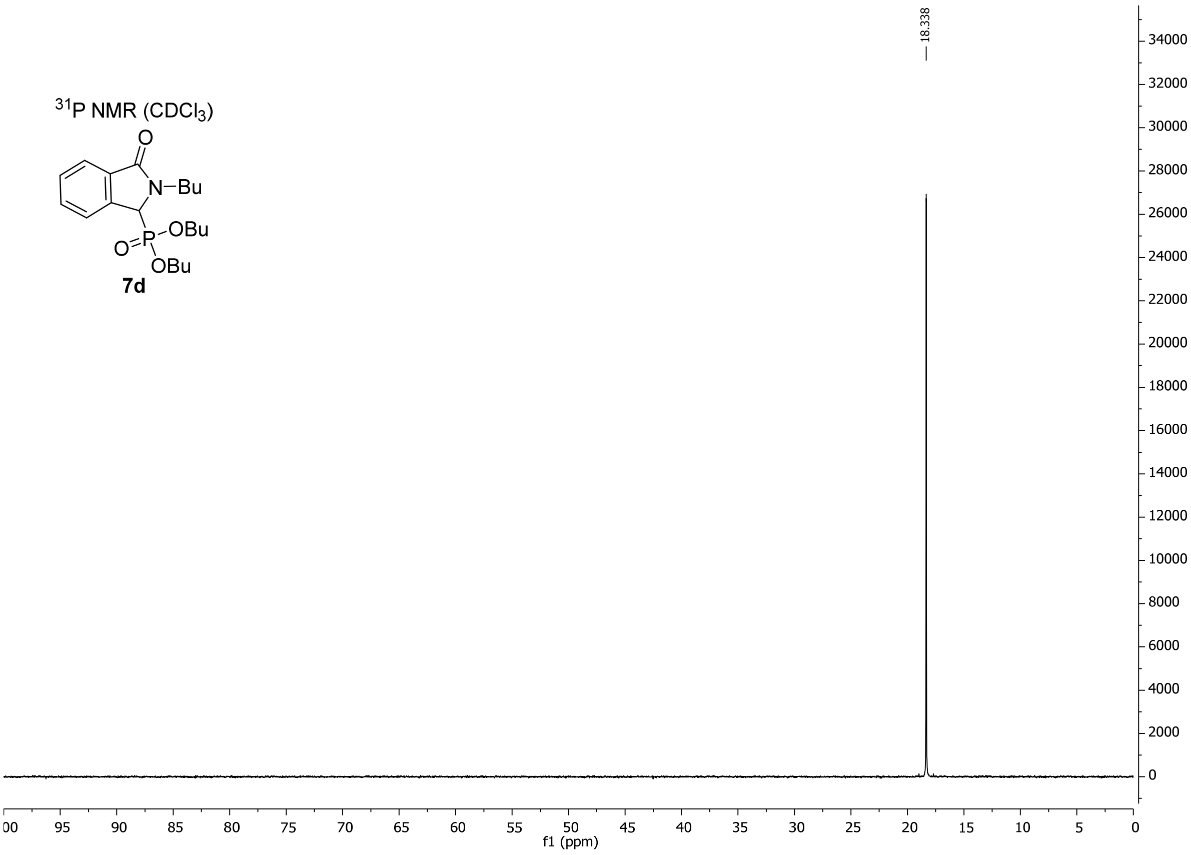
18.338
^{31}P NMR (CDCl$_3$)
O
N–Bu
O=P
OBu
OBu
7d
f1 (ppm)

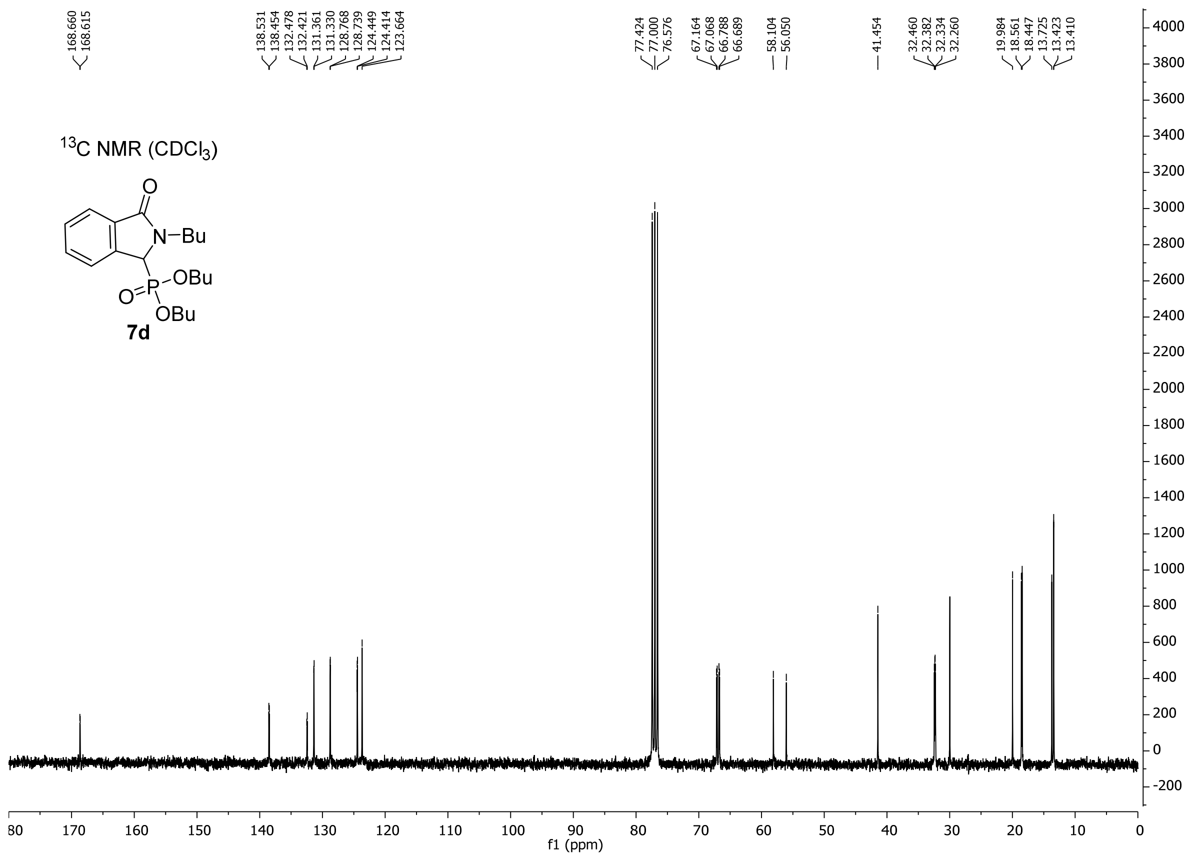

168.660
168.615
138.531
138.454
132.478
132.421
131.361
131.330
128.768
128.739
124.449
124.414
123.664
77.424
77.000
76.576
67.164
67.068
66.788
66.689
58.104
56.050
41.454
32.460
32.382
32.334
32.260
19.984
18.561
18.447
13.725
13.423
13.410
^{13}C NMR (CDCl$_3$)
N–Bu
OBu
OBu
7d
f1 (ppm)

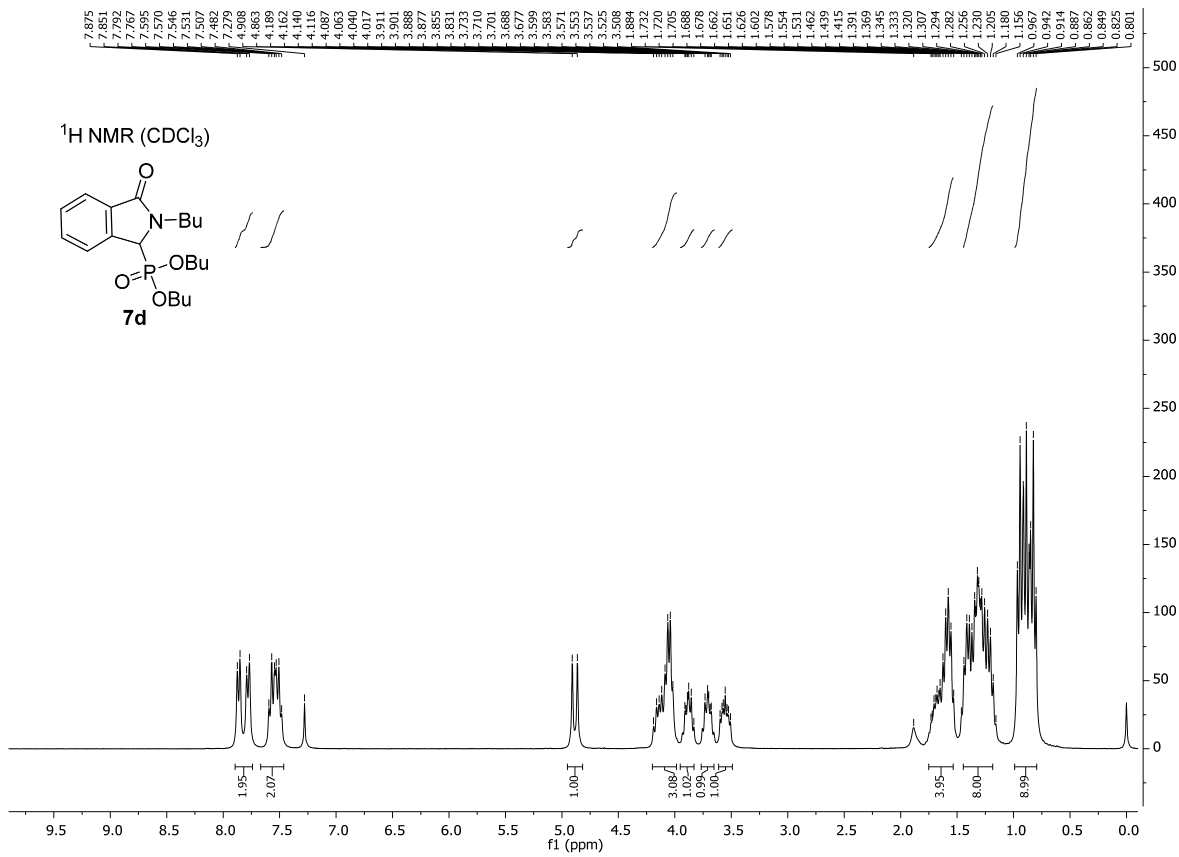

^{1}H NMR (CDCl$_3$)
N–Bu
OBu
OBu
7d
1.95
2.07
1.00
3.08
1.02
0.99
1.00
3.95
8.00
8.99
f1 (ppm)

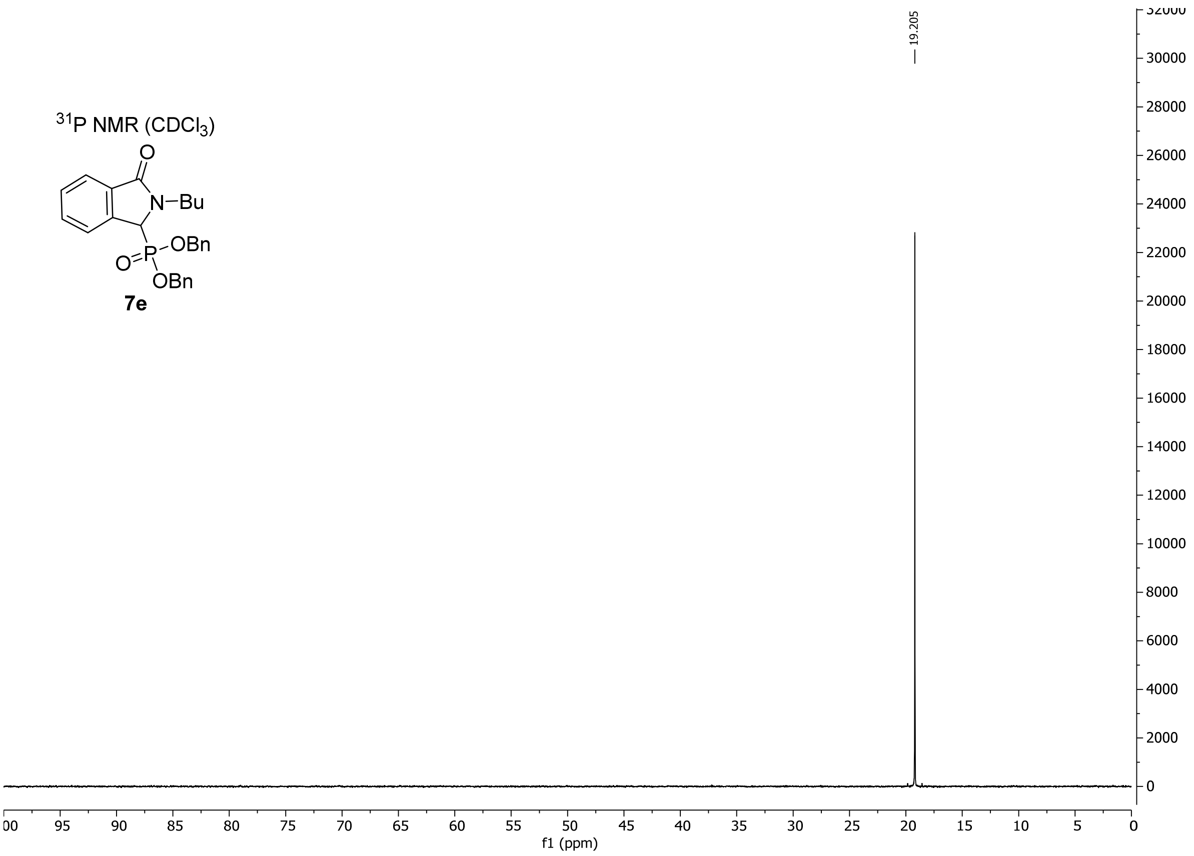

31P NMR (CDCl3)
N–Bu
OBn
OBn
7e
19.205
f1 (ppm)

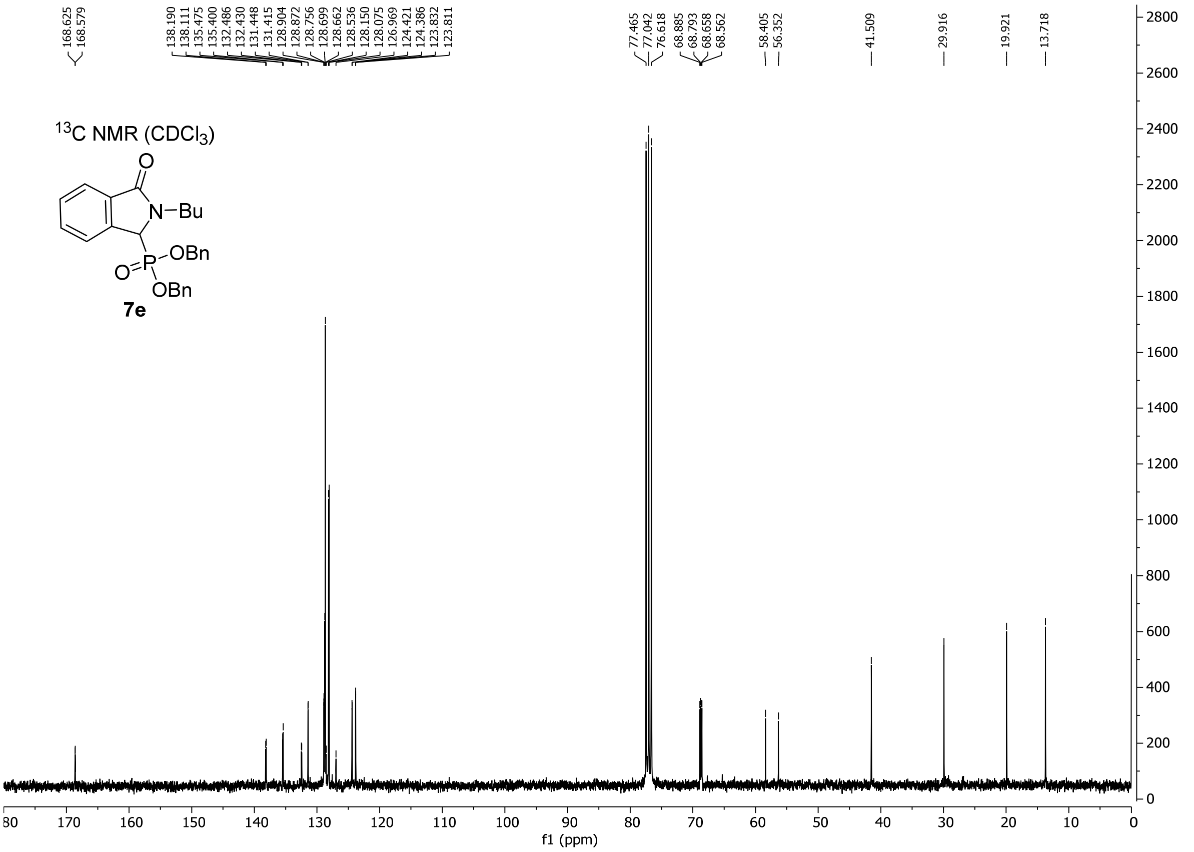

13C NMR (CDCl3)
N–Bu
OBn
OBn
7e
f1 (ppm)

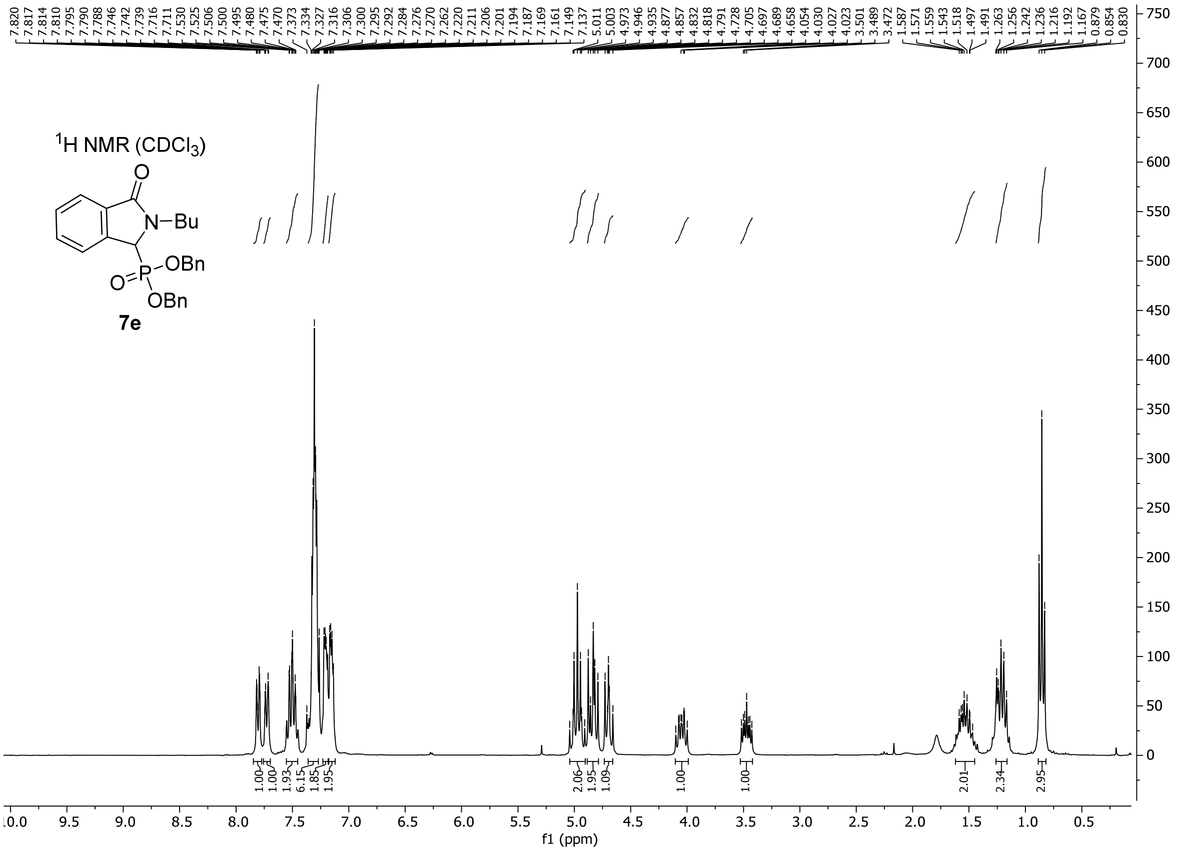

^{1}H NMR (CDCl$_3$)
N–Bu
OBn
OBn
7e
f1 (ppm)

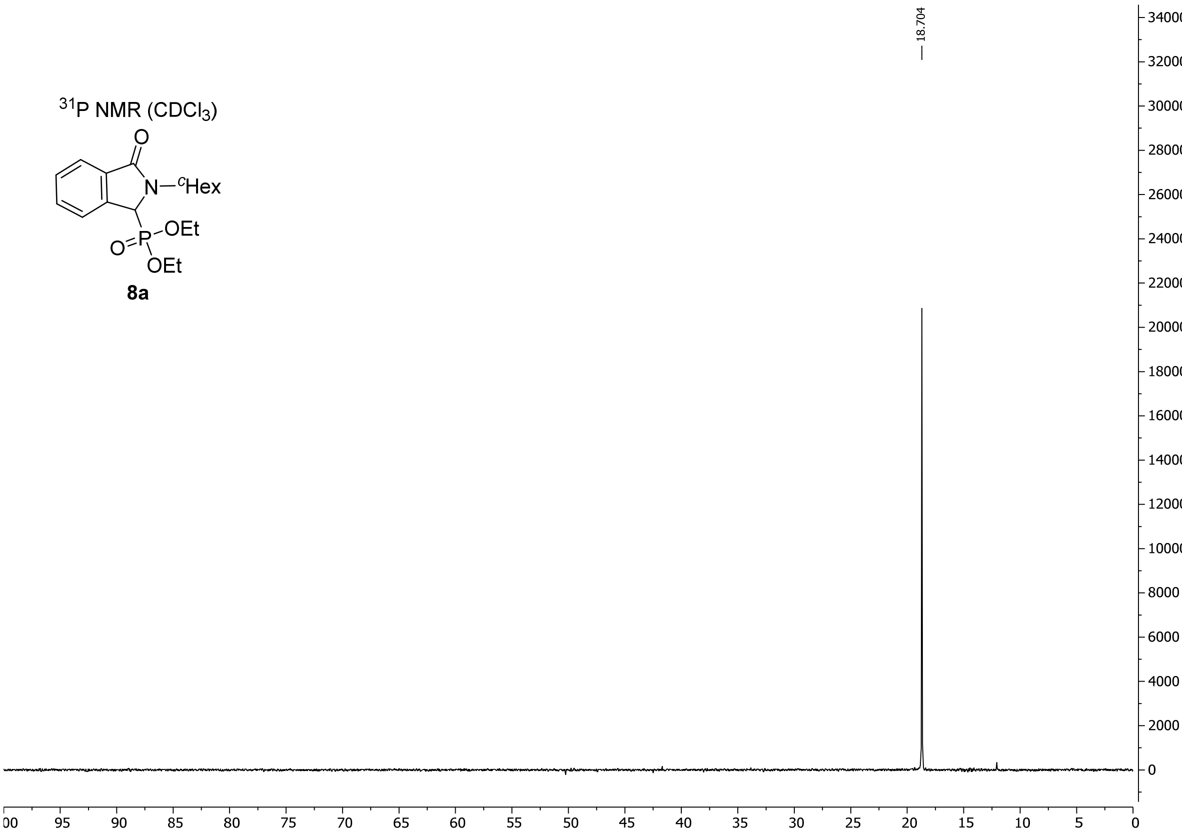

^{31}P NMR (CDCl$_3$)
N–^{c}Hex
OEt
OEt
8a
18.704
f1 (ppm)

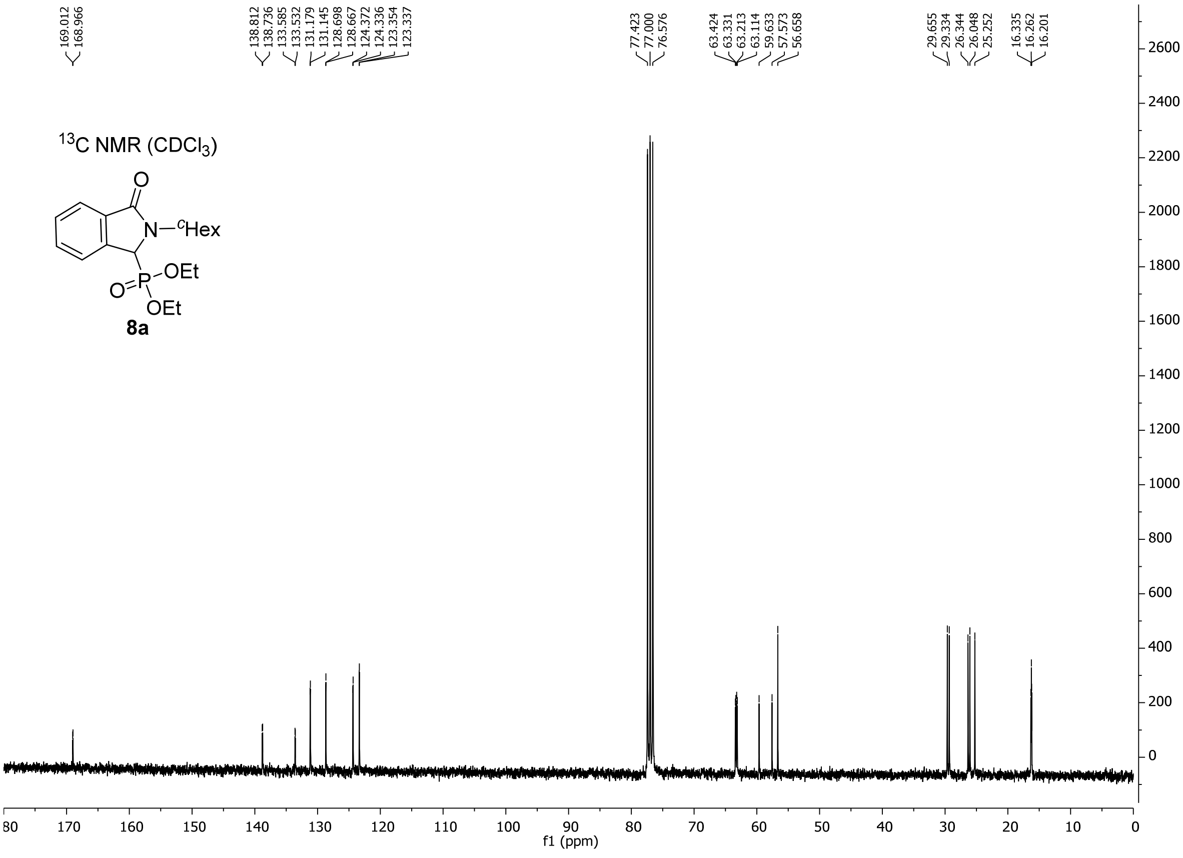

^{13}C NMR (CDCl$_3$)
N—^{c}Hex
OEt
OEt
8a
169.012
168.966
138.812
138.736
133.585
133.532
131.179
131.145
128.698
128.667
124.372
124.336
123.354
123.337
77.423
77.000
76.576
63.424
63.331
63.213
63.114
59.633
57.573
56.658
29.655
29.334
26.344
26.048
25.252
16.335
16.262
16.201
f1 (ppm)

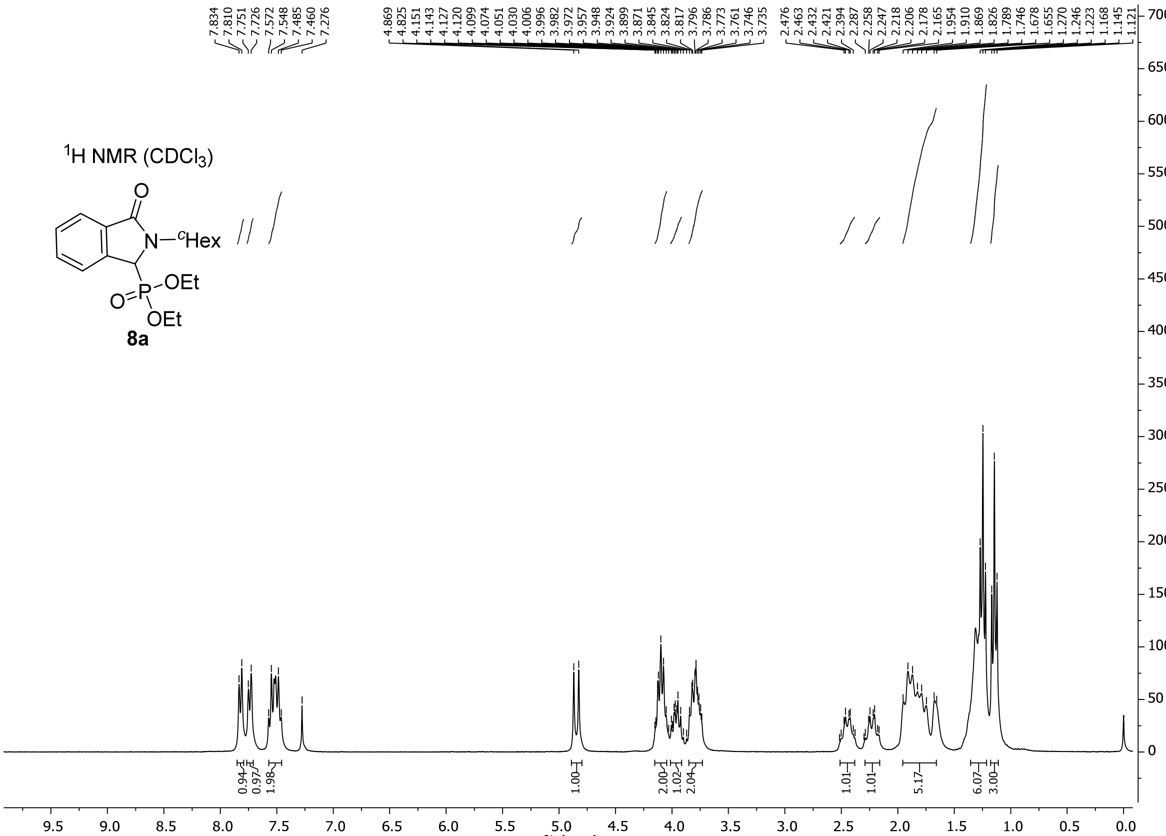

^{1}H NMR (CDCl$_3$)
N—^{c}Hex
OEt
OEt
8a
7.834
7.810
7.751
7.726
7.572
7.548
7.485
7.460
7.276
4.869
4.825
4.151
4.143
4.127
4.120
4.099
4.074
4.051
4.030
4.006
3.996
3.982
3.972
3.957
3.948
3.924
3.899
3.871
3.845
3.824
3.817
3.796
3.786
3.773
3.761
3.746
3.735
2.476
2.463
2.432
2.421
2.394
2.287
2.258
2.247
2.218
2.206
2.178
2.165
1.954
1.910
1.869
1.826
1.789
1.746
1.678
1.655
1.270
1.246
1.223
1.168
1.145
1.121
0.94
0.97
1.98
1.00
2.00
1.02
2.04
1.01
1.01
5.17
6.07
3.00
f1 (ppm)

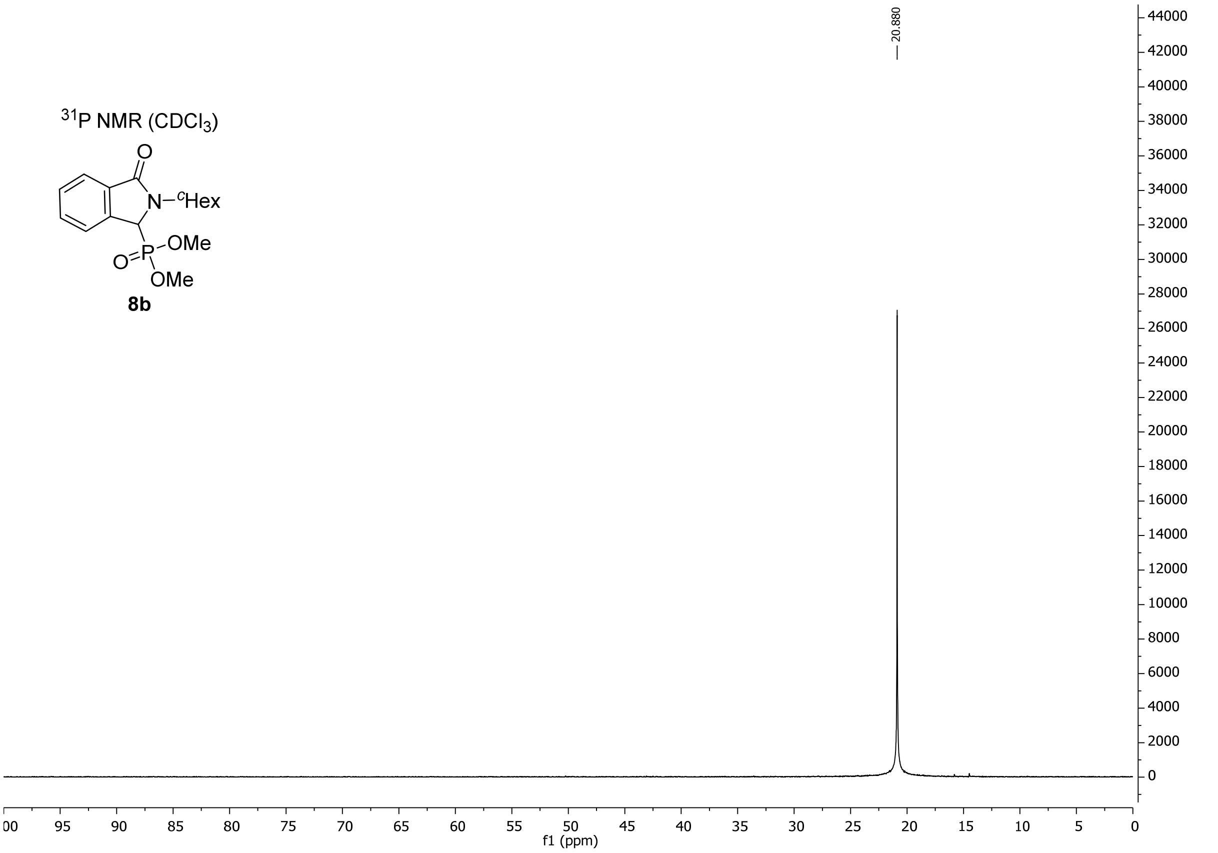

20.880
31P NMR (CDCl3)
N—cHex
OMe
OMe
8b
f1 (ppm)

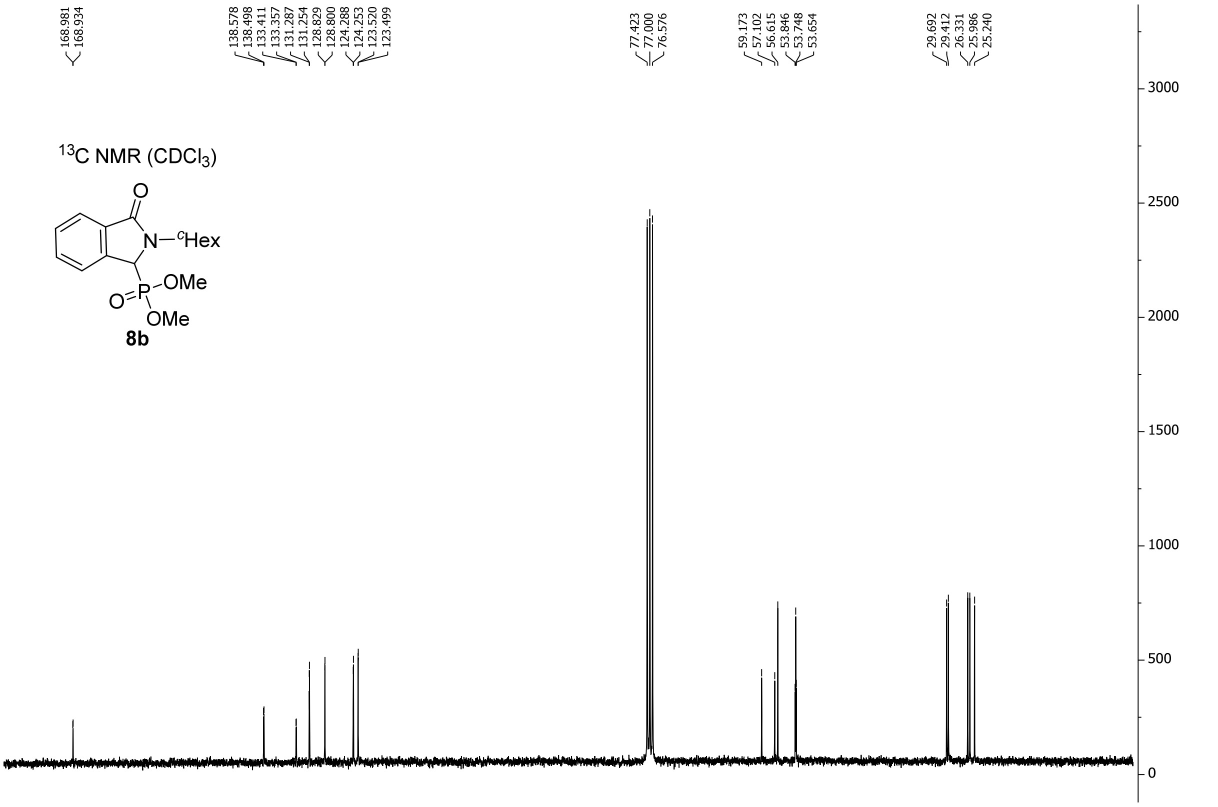

168.981
168.934
138.578
138.498
133.411
133.357
131.287
131.254
128.829
128.800
124.288
124.253
123.520
123.499
77.423
77.000
76.576
59.173
57.102
56.615
53.846
53.748
53.654
29.692
29.412
26.331
25.986
25.240
13C NMR (CDCl3)
N—cHex
OMe
OMe
8b
f1 (ppm)

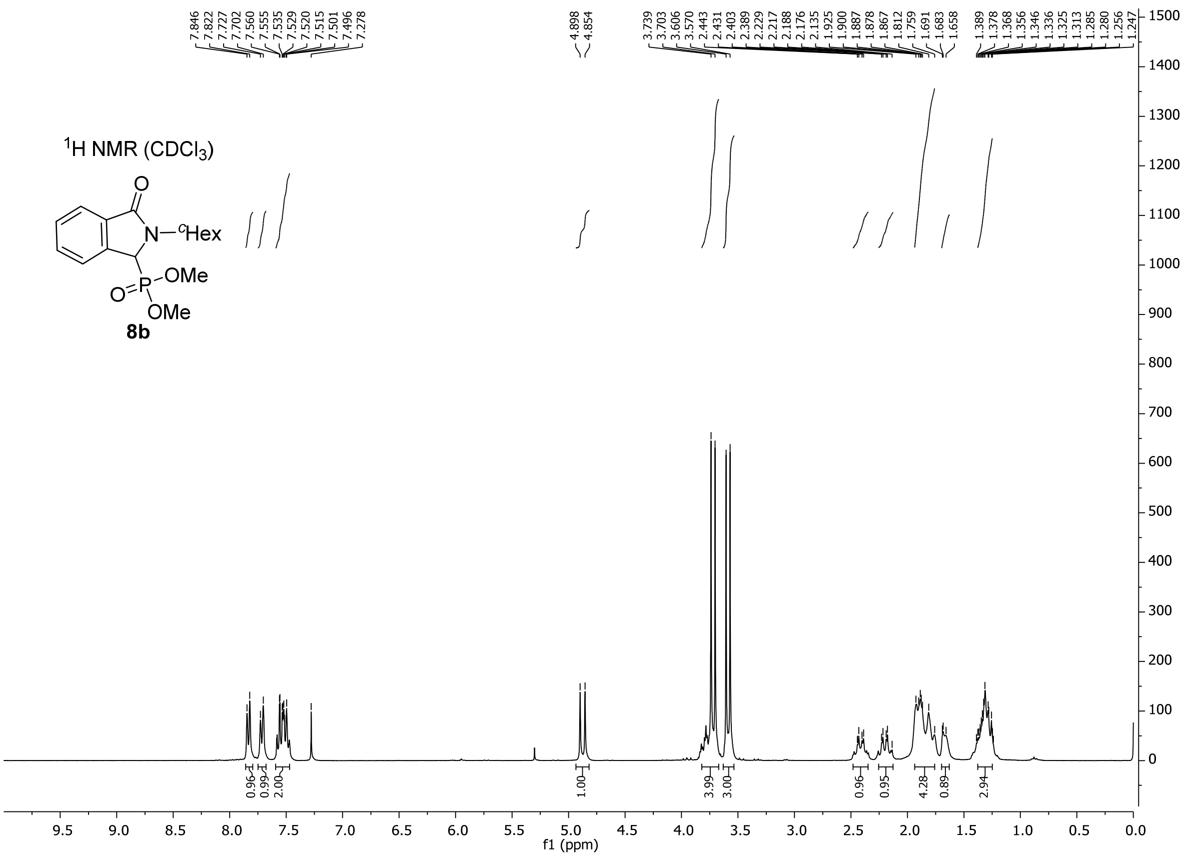
1H NMR (CDCl3)
N—cHex
OMe
OMe
8b
f1 (ppm)

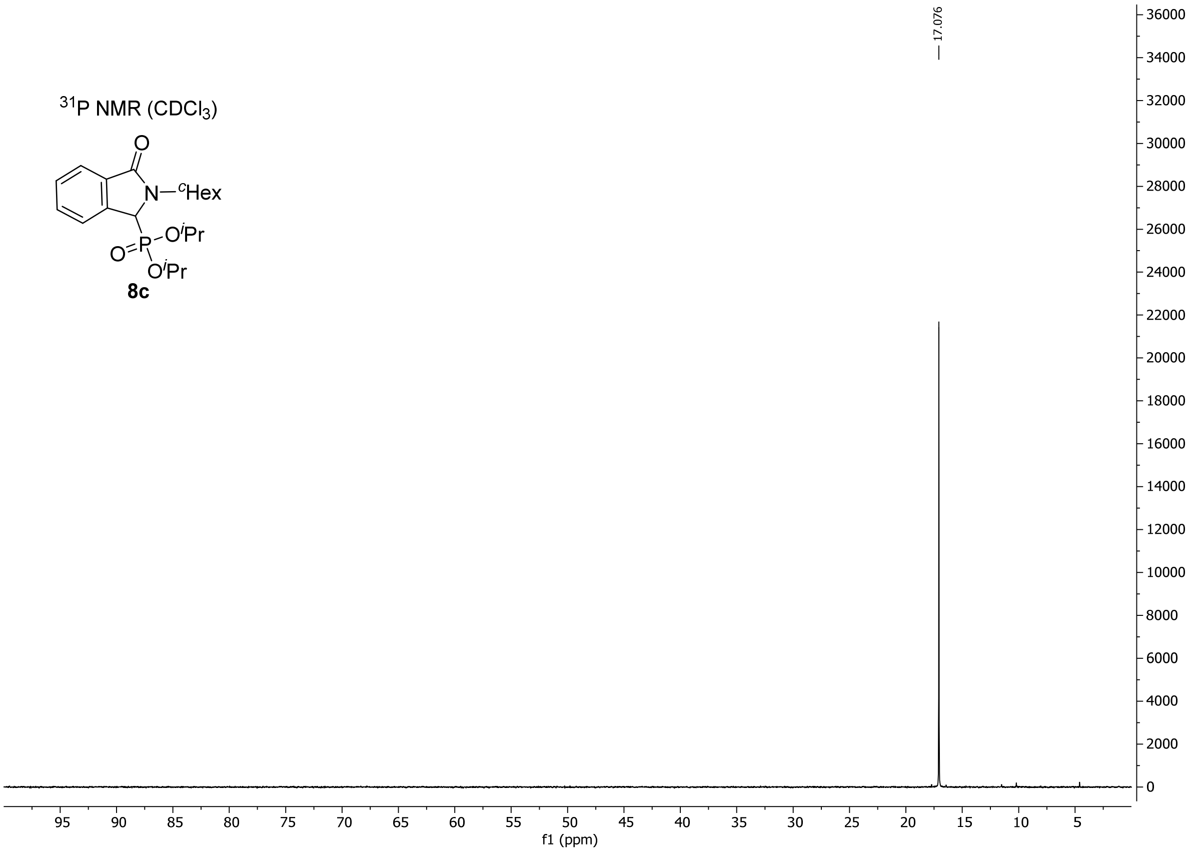
31P NMR (CDCl3)
N—cHex
OiPr
OiPr
8c
17.076
f1 (ppm)

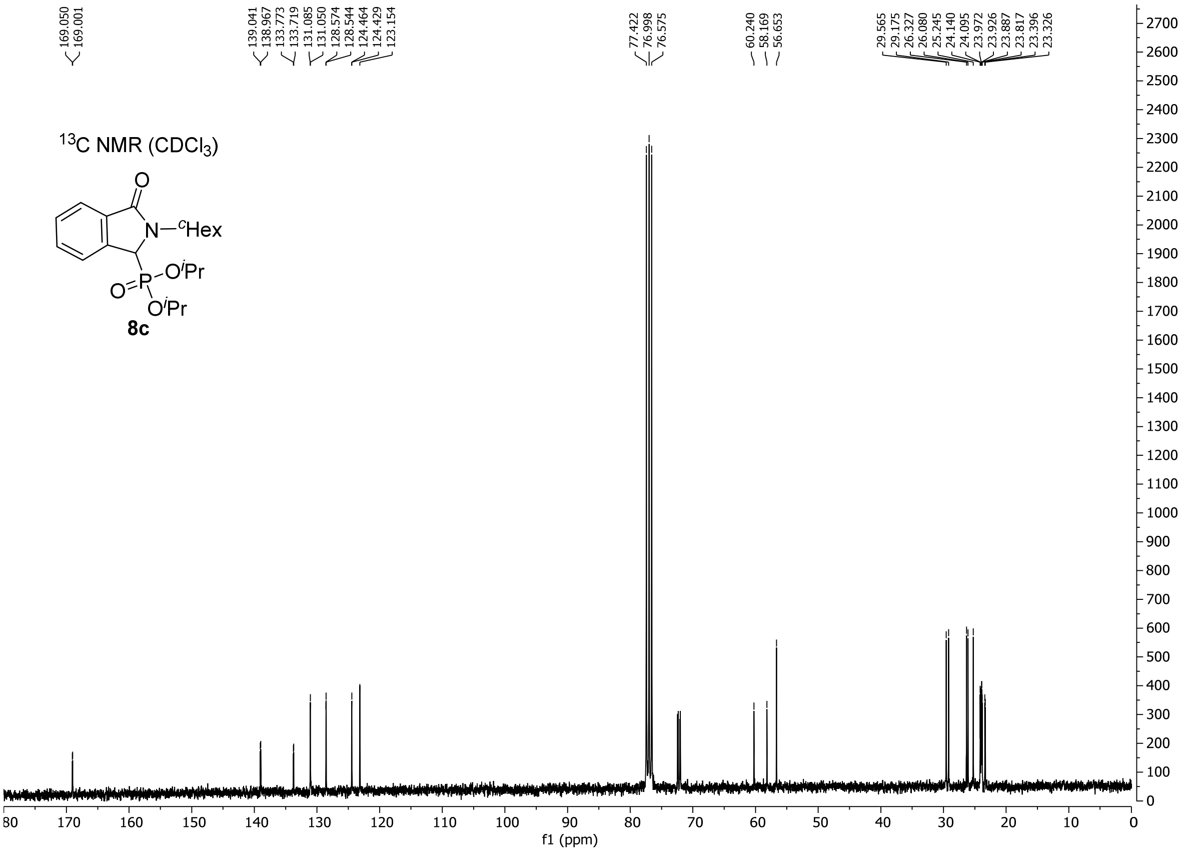

^{13}C NMR (CDCl$_3$)
N—^{c}Hex
O^{i}Pr
O^{i}Pr
8c
f1 (ppm)

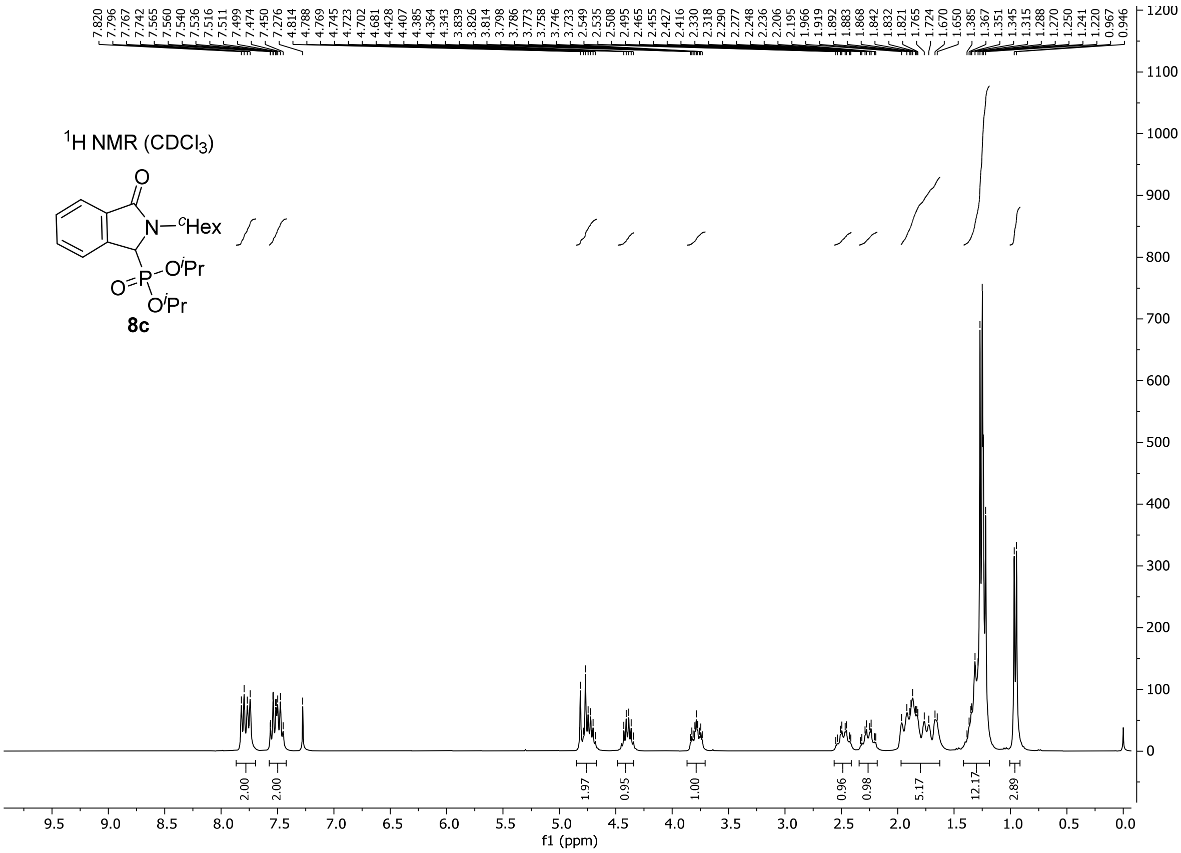

^{1}H NMR (CDCl$_3$)
N—^{c}Hex
O^{i}Pr
O^{i}Pr
8c
f1 (ppm)

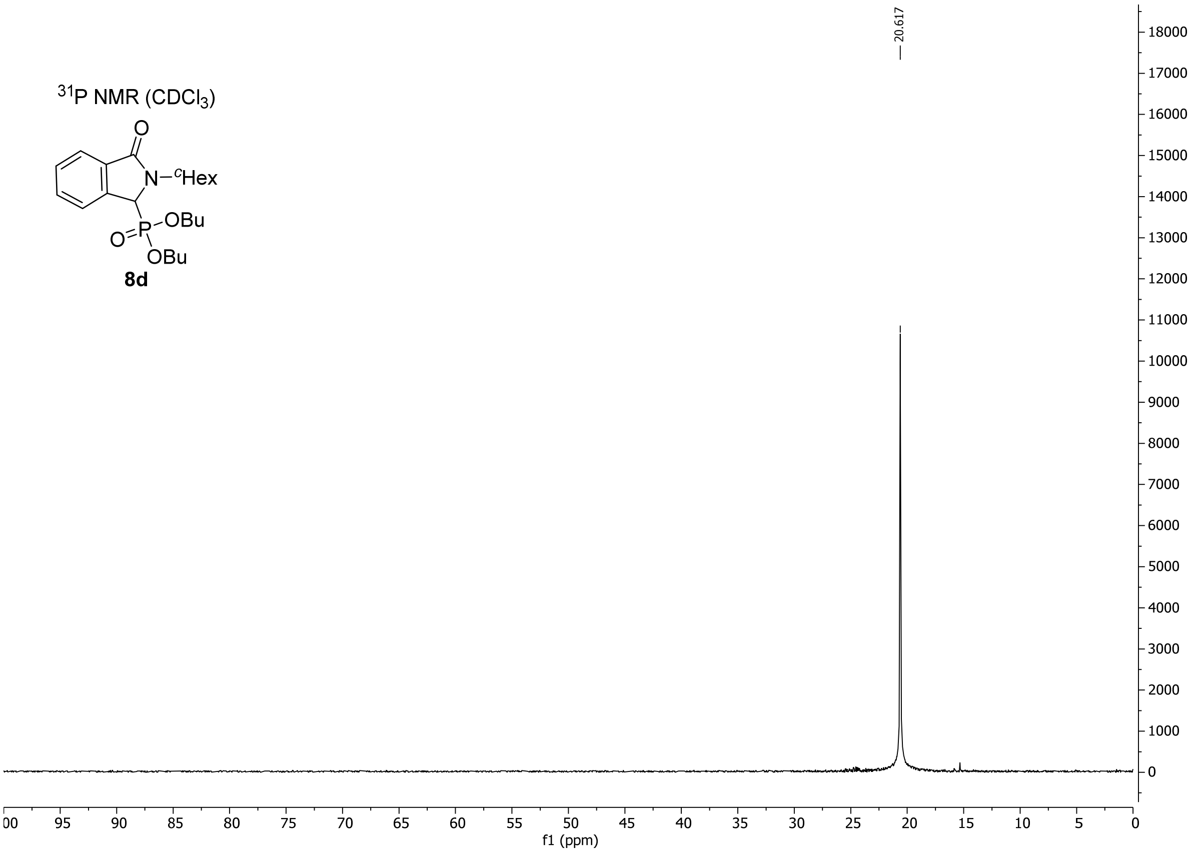
31P NMR (CDCl3)
N–cHex
OBu
OBu
8d
20.617
f1 (ppm)

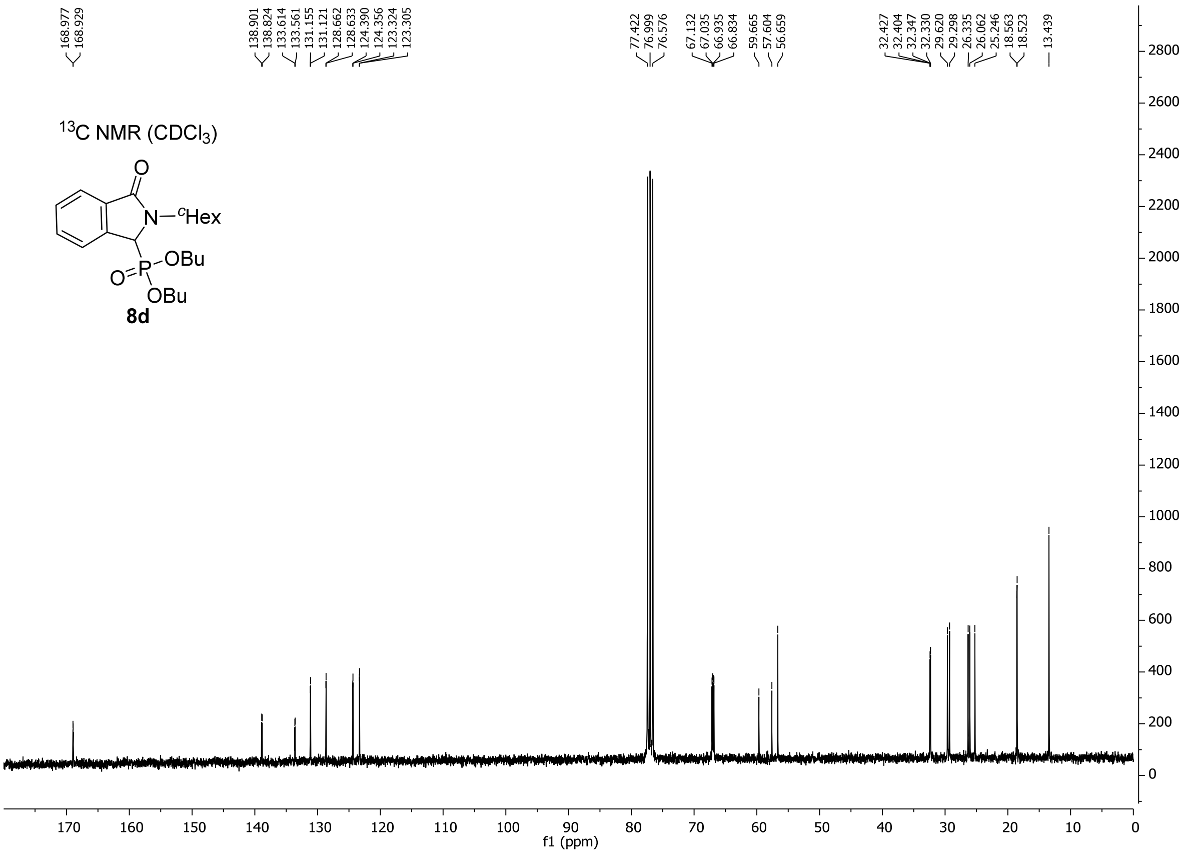
13C NMR (CDCl3)
N–cHex
OBu
OBu
8d
168.977
168.929
138.901
138.824
133.614
133.561
131.155
131.121
128.662
128.633
124.390
124.356
123.324
123.305
77.422
76.999
76.576
67.132
67.035
66.935
66.834
59.665
57.604
56.659
32.427
32.404
32.347
32.330
29.620
29.298
26.335
26.062
25.246
18.563
18.523
13.439
f1 (ppm)

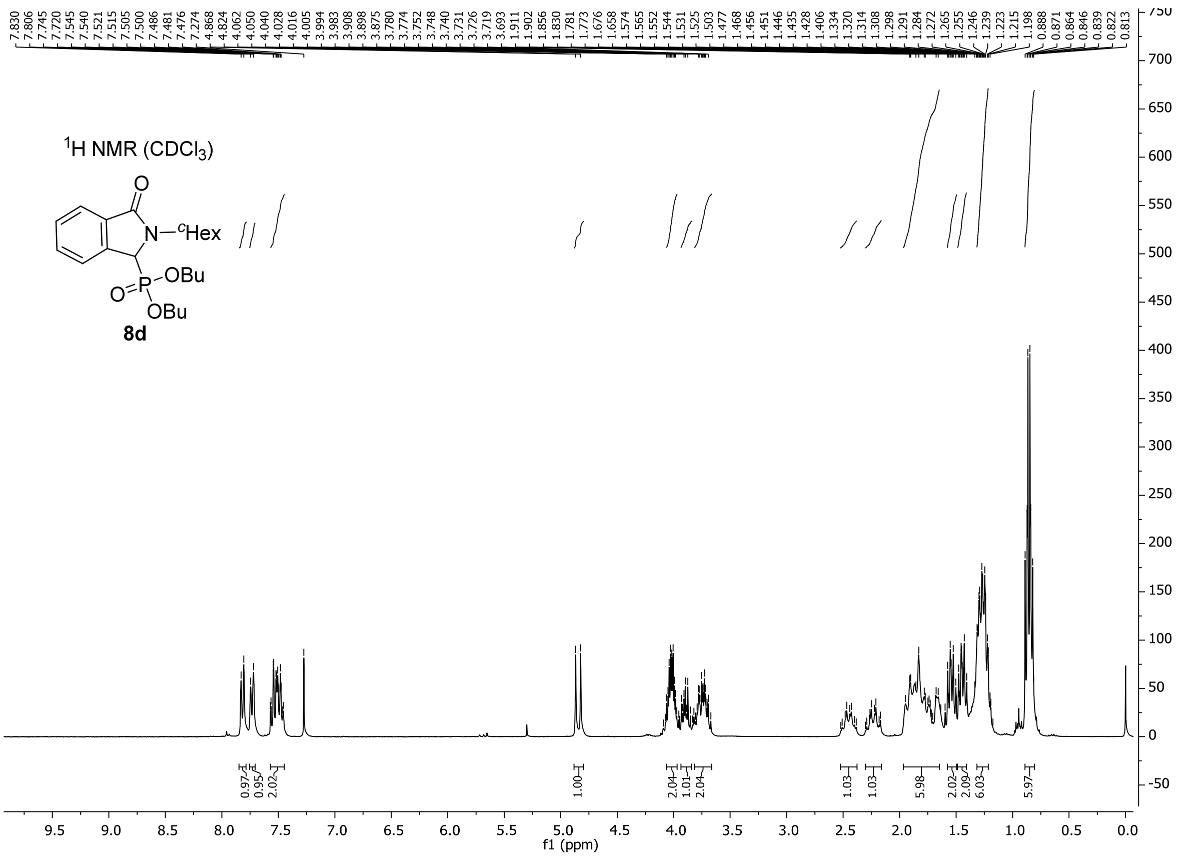

1H NMR (CDCl3)
N—cHex
OBu
OBu
8d
f1 (ppm)

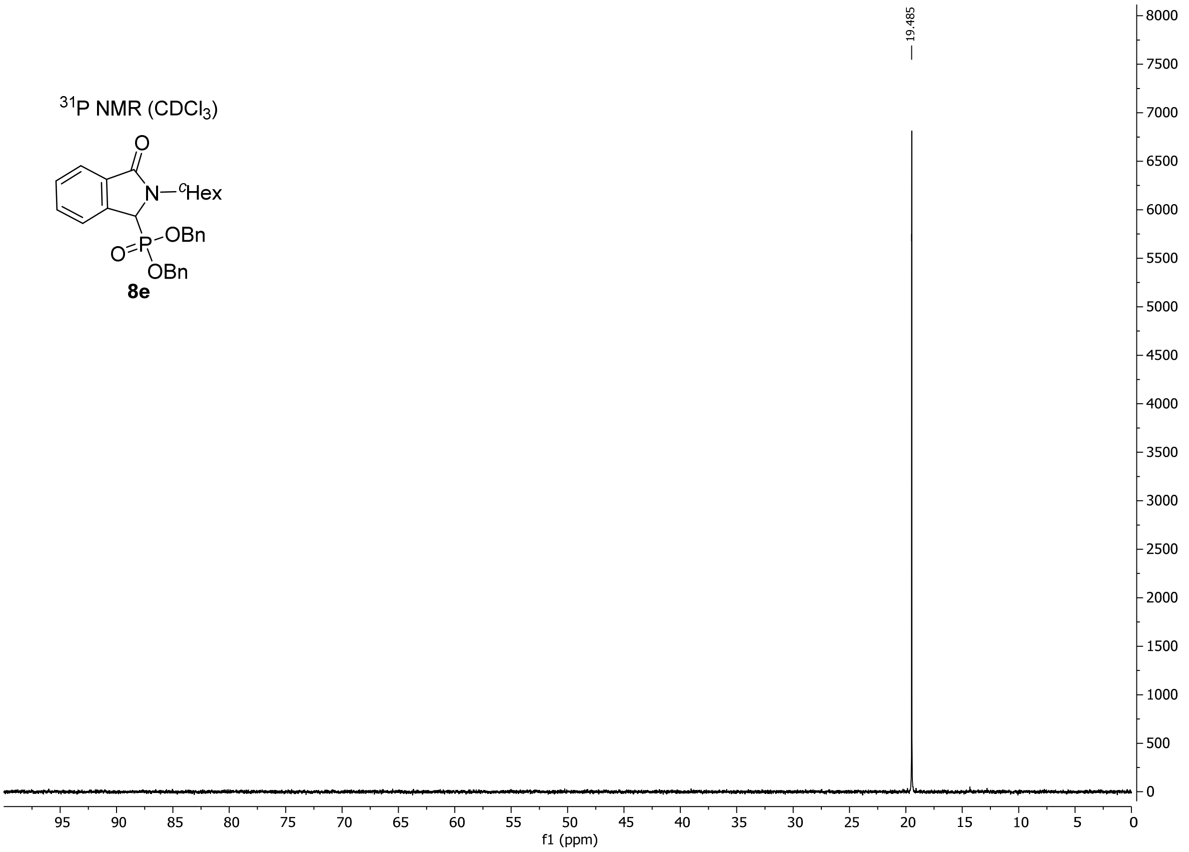

31P NMR (CDCl3)
N—cHex
OBn
OBn
8e
19.485
f1 (ppm)

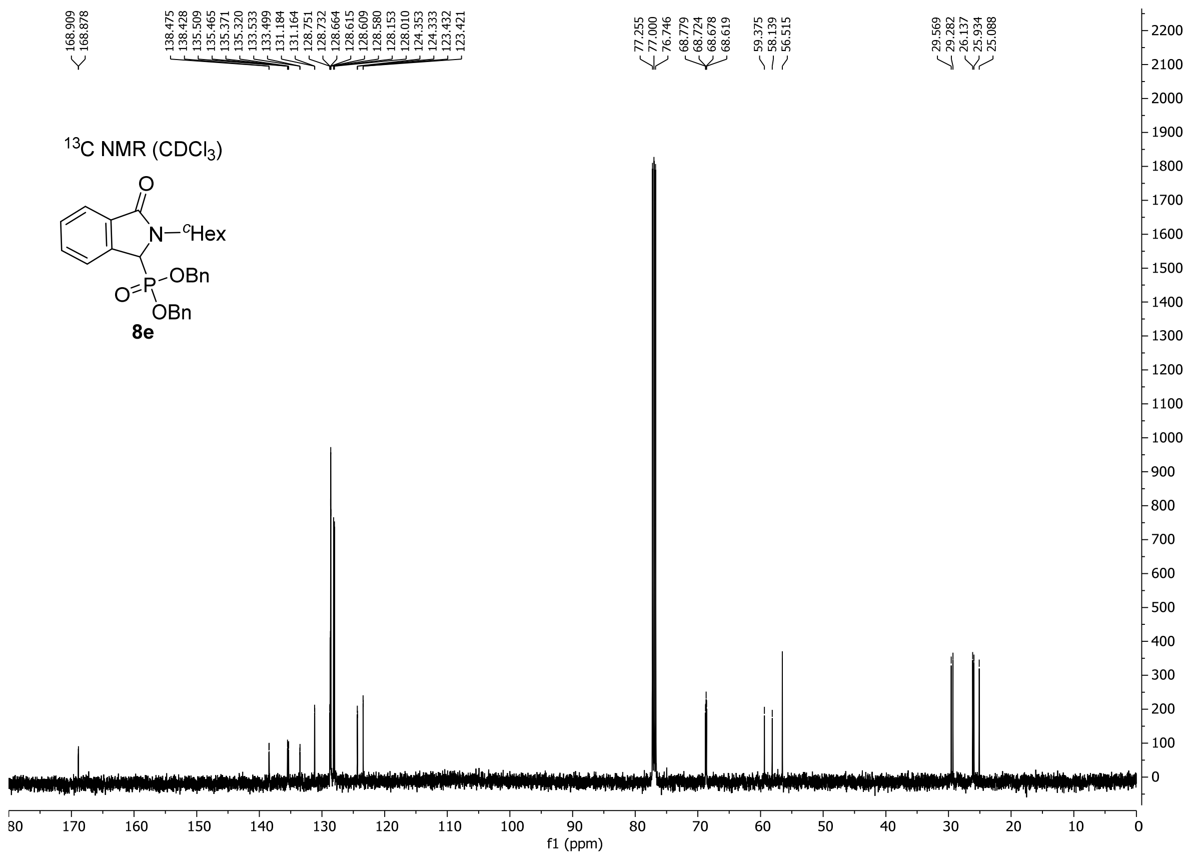
13C NMR (CDCl3)
N—cHex
OBn
OBn
8e
168.909
168.878
138.475
138.428
135.509
135.465
135.371
135.320
133.533
133.499
131.184
131.164
128.751
128.732
128.664
128.615
128.609
128.580
128.153
128.010
124.353
124.333
123.432
123.421
77.255
77.000
76.746
68.779
68.724
68.678
68.619
59.375
58.139
56.515
29.569
29.282
26.137
25.934
25.088
f1 (ppm)

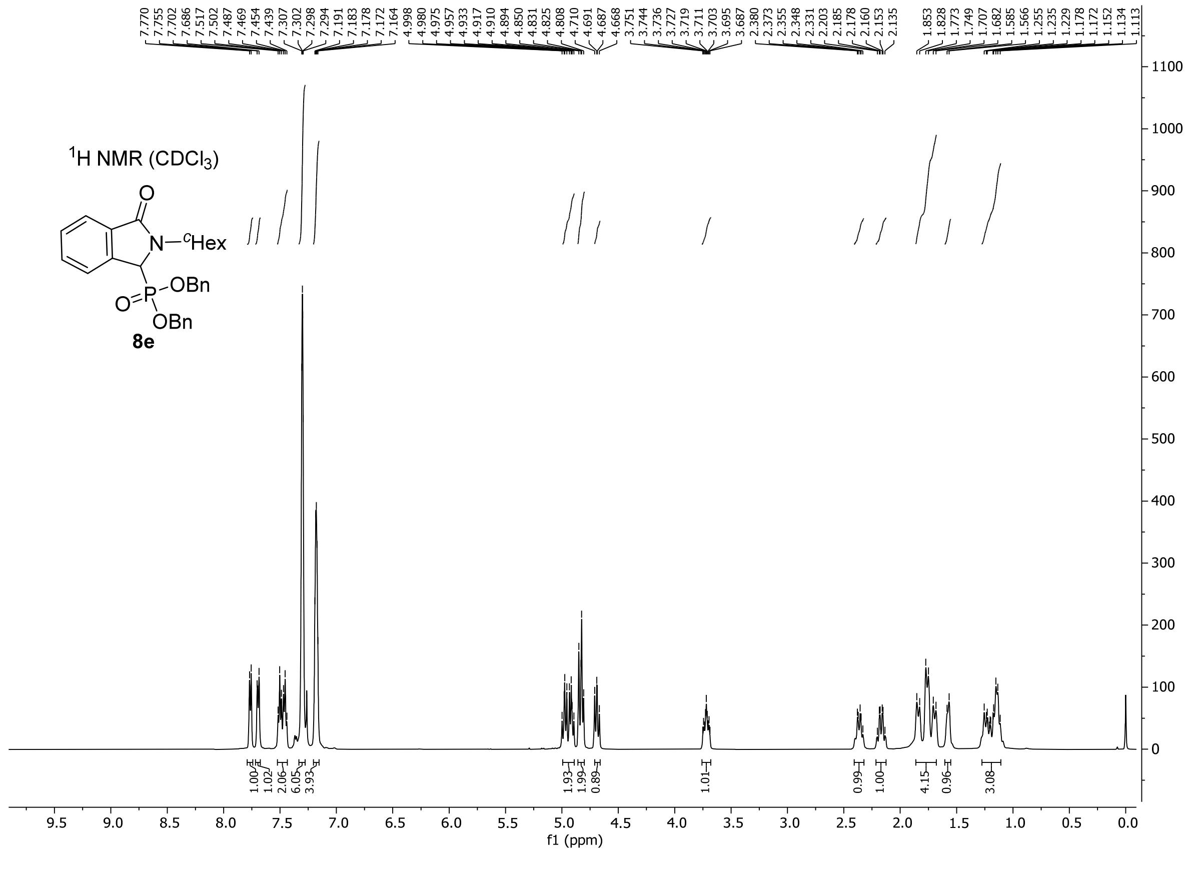
1H NMR (CDCl3)
N—cHex
OBn
OBn
8e
7.770
7.755
7.702
7.686
7.517
7.502
7.487
7.469
7.454
7.439
7.307
7.302
7.298
7.294
7.191
7.183
7.178
7.172
7.164
4.998
4.980
4.975
4.957
4.933
4.917
4.910
4.894
4.850
4.831
4.825
4.808
4.710
4.691
4.687
4.668
3.751
3.744
3.736
3.727
3.719
3.711
3.703
3.695
3.687
2.380
2.373
2.355
2.348
2.331
2.203
2.185
2.178
2.160
2.153
2.135
1.853
1.828
1.773
1.749
1.707
1.682
1.585
1.566
1.255
1.235
1.229
1.178
1.172
1.152
1.134
1.113
1.00
1.02
2.06
6.05
3.93
1.93
1.99
0.89
1.01
0.99
1.00
4.15
0.96
3.08
f1 (ppm)

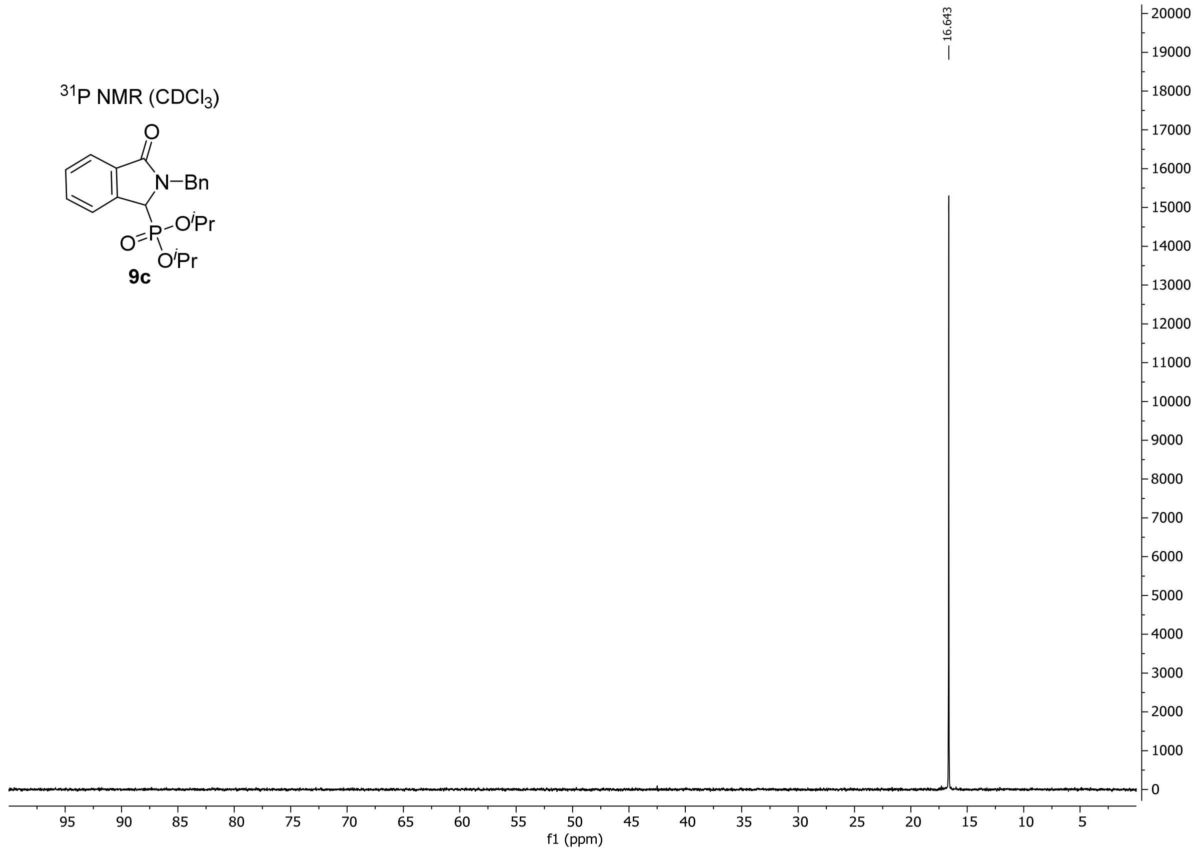

16.643
^{31}P NMR (CDCl$_3$)
O
N−Bn
O=P−O^{i}Pr
O^{i}Pr
9c
f1 (ppm)

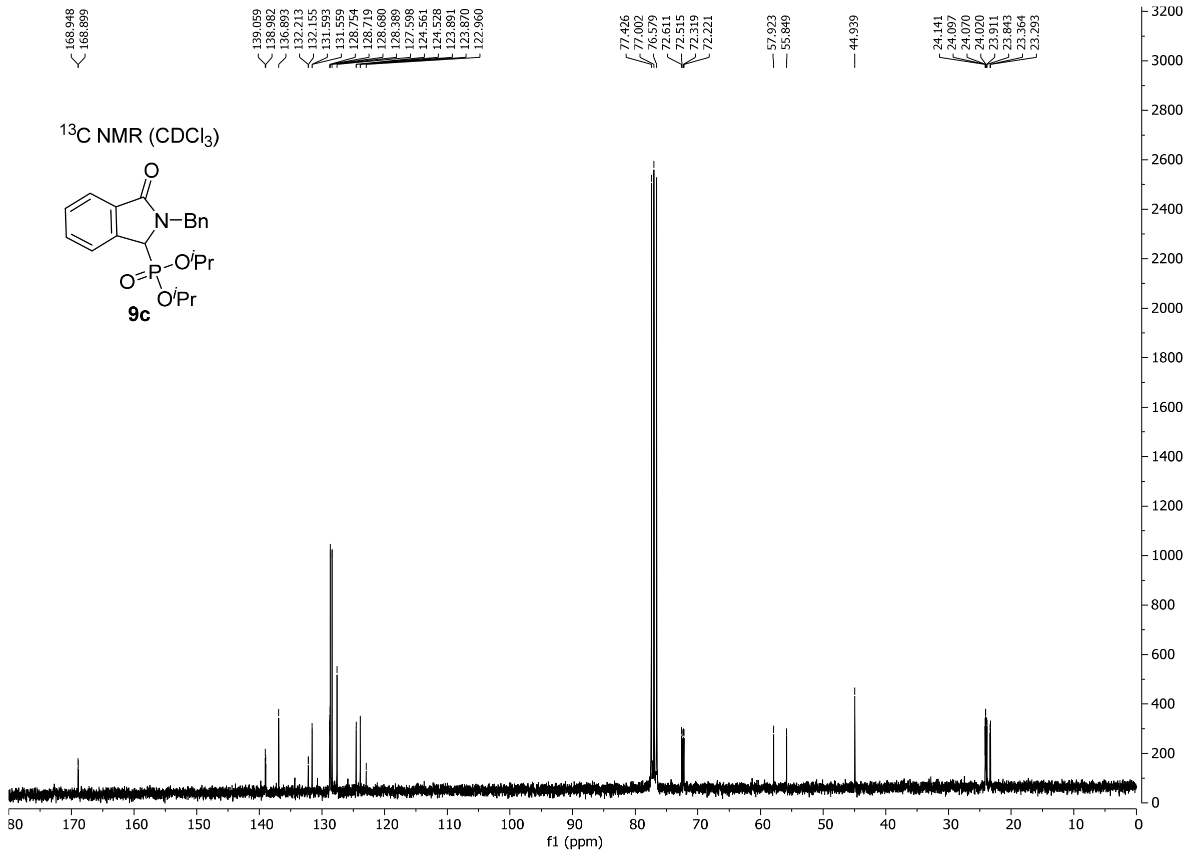

168.948
168.899
139.059
138.982
136.893
132.213
132.155
131.593
131.559
128.754
128.719
128.680
128.389
127.598
124.561
124.528
123.891
123.870
122.960
77.426
77.002
76.579
72.611
72.515
72.319
72.221
57.923
55.849
44.939
24.141
24.097
24.070
24.020
23.911
23.843
23.364
23.293
^{13}C NMR (CDCl$_3$)
O
N−Bn
O=P−O^{i}Pr
O^{i}Pr
9c
f1 (ppm)

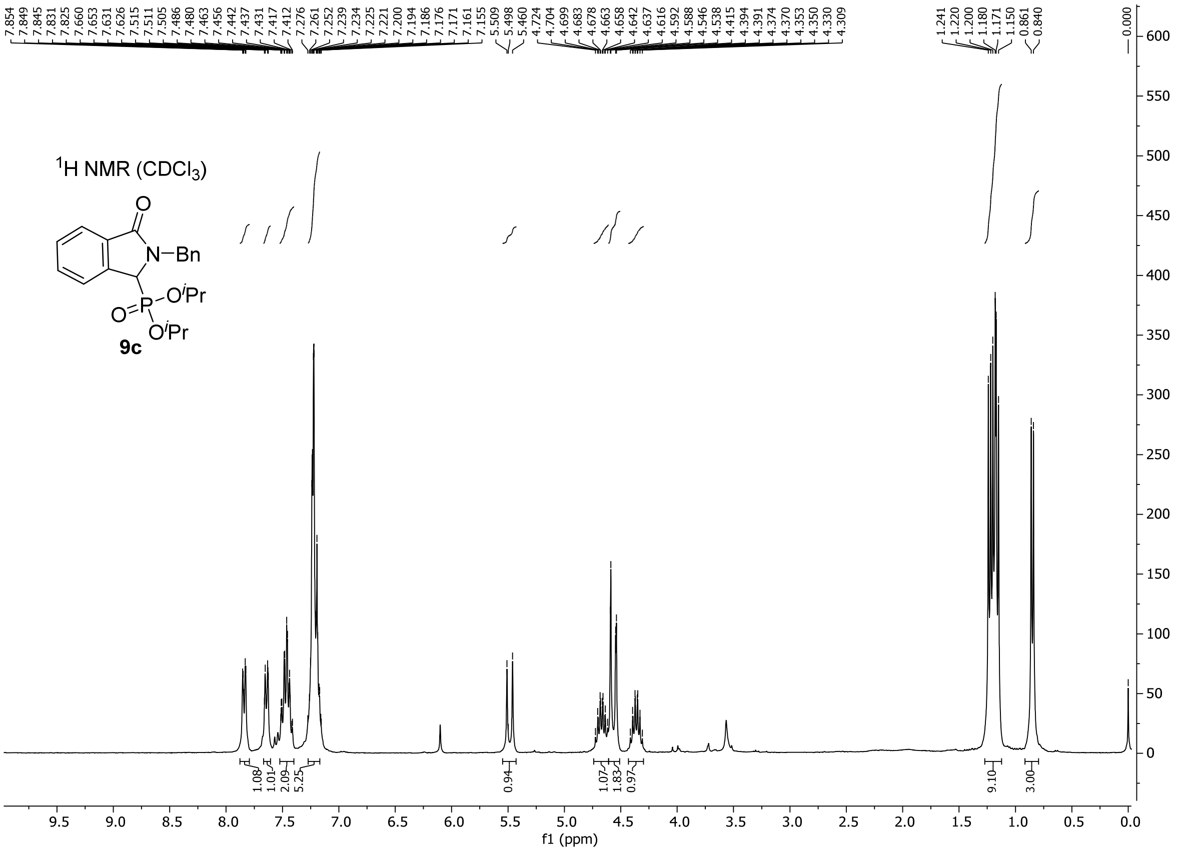

^{1}H NMR ($CDCl_3$)
N−Bn
O^{i}Pr
O^{i}Pr
9c
f1 (ppm)

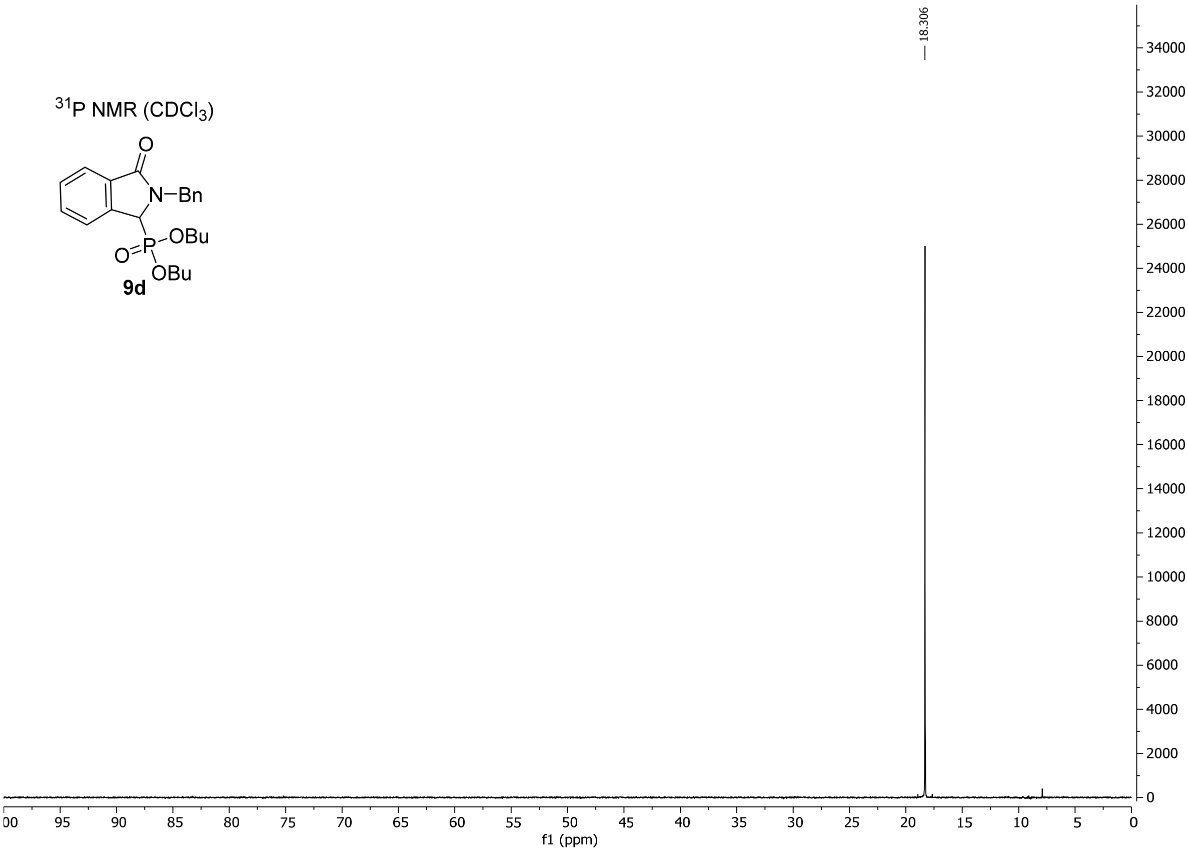

^{31}P NMR ($CDCl_3$)
N−Bn
OBu
OBu
9d
18.306
f1 (ppm)

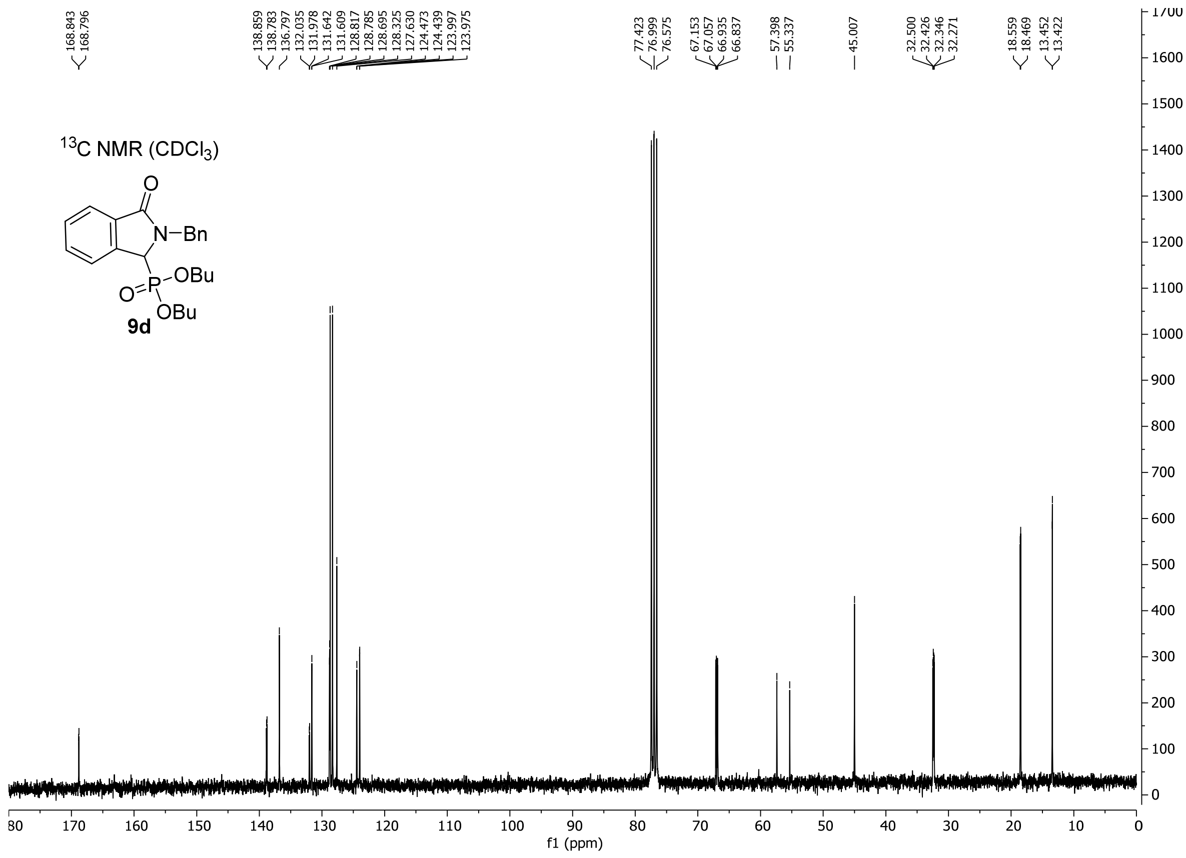

13C NMR (CDCl3)
N−Bn
OBu
OBu
9d
f1 (ppm)

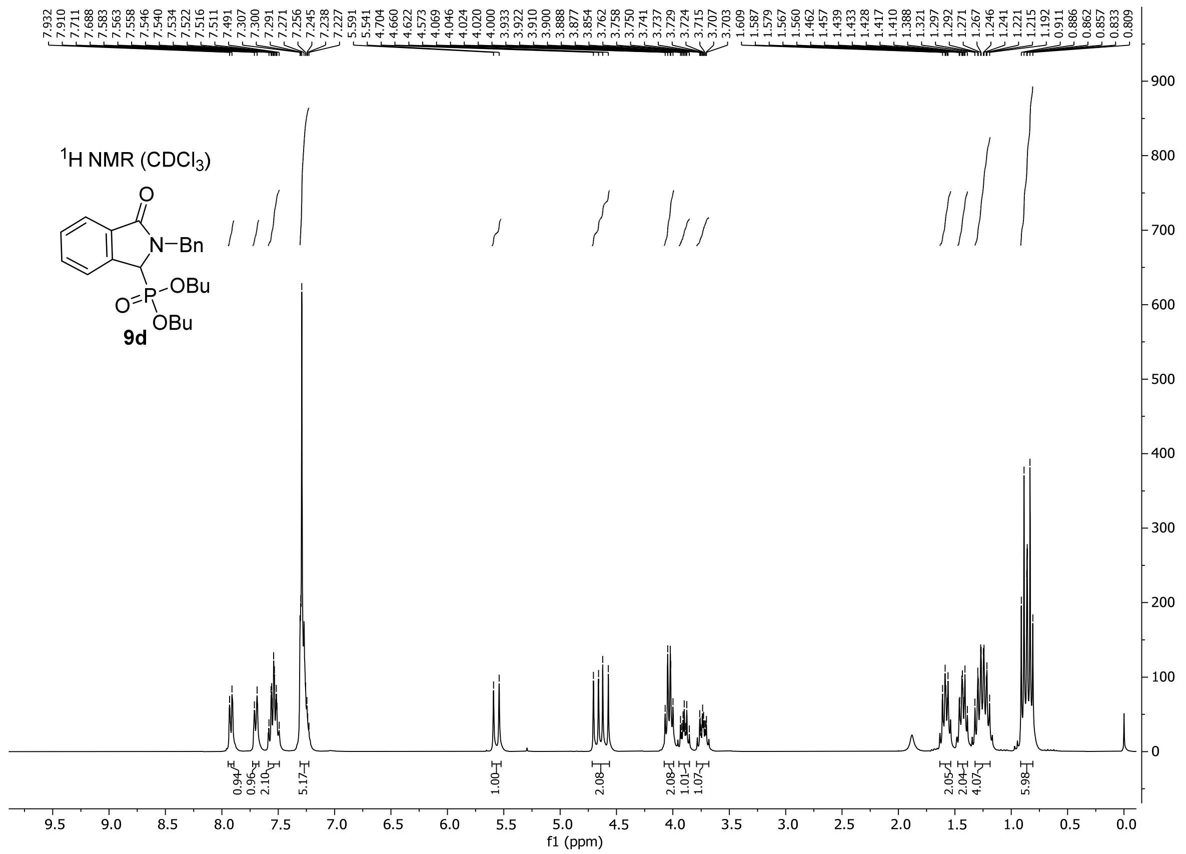

1H NMR (CDCl3)
N−Bn
OBu
OBu
9d
f1 (ppm)

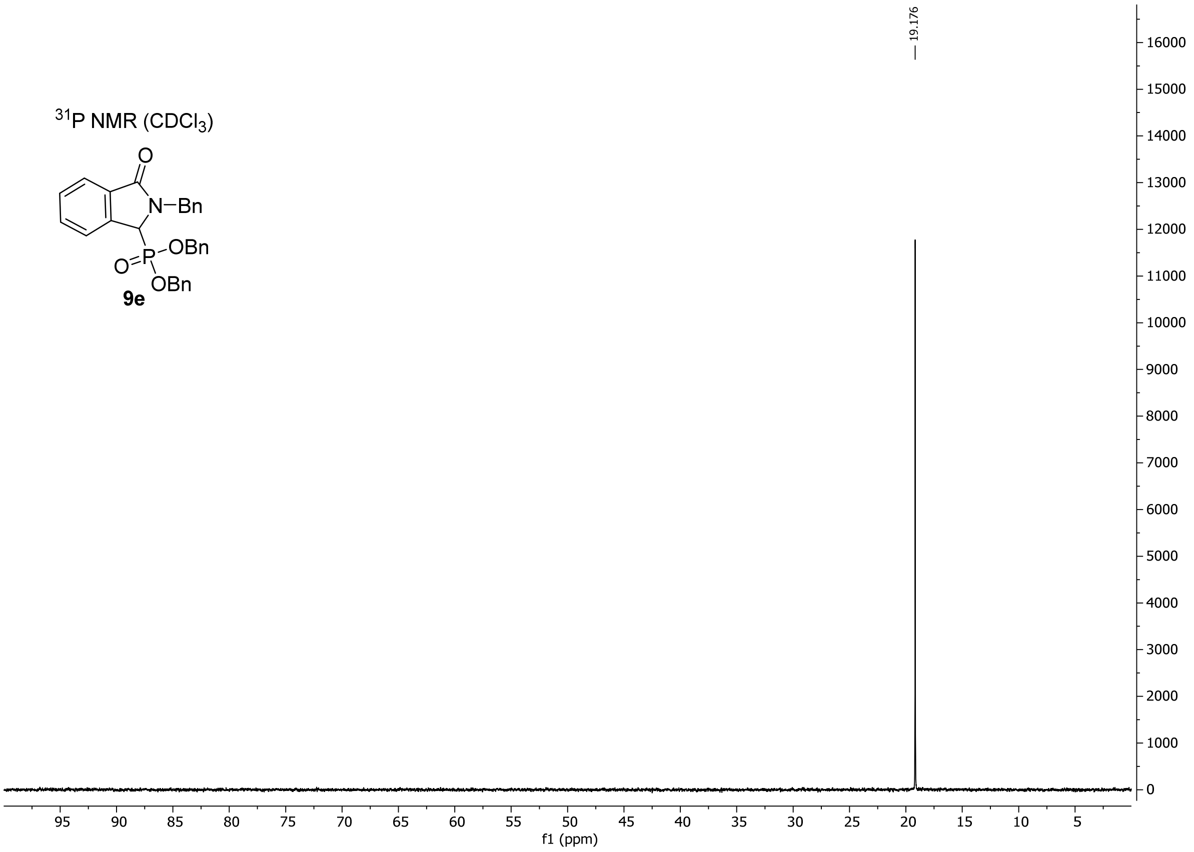

31P NMR (CDCl3)
9e
N−Bn
OBn
OBn
19.176
f1 (ppm)

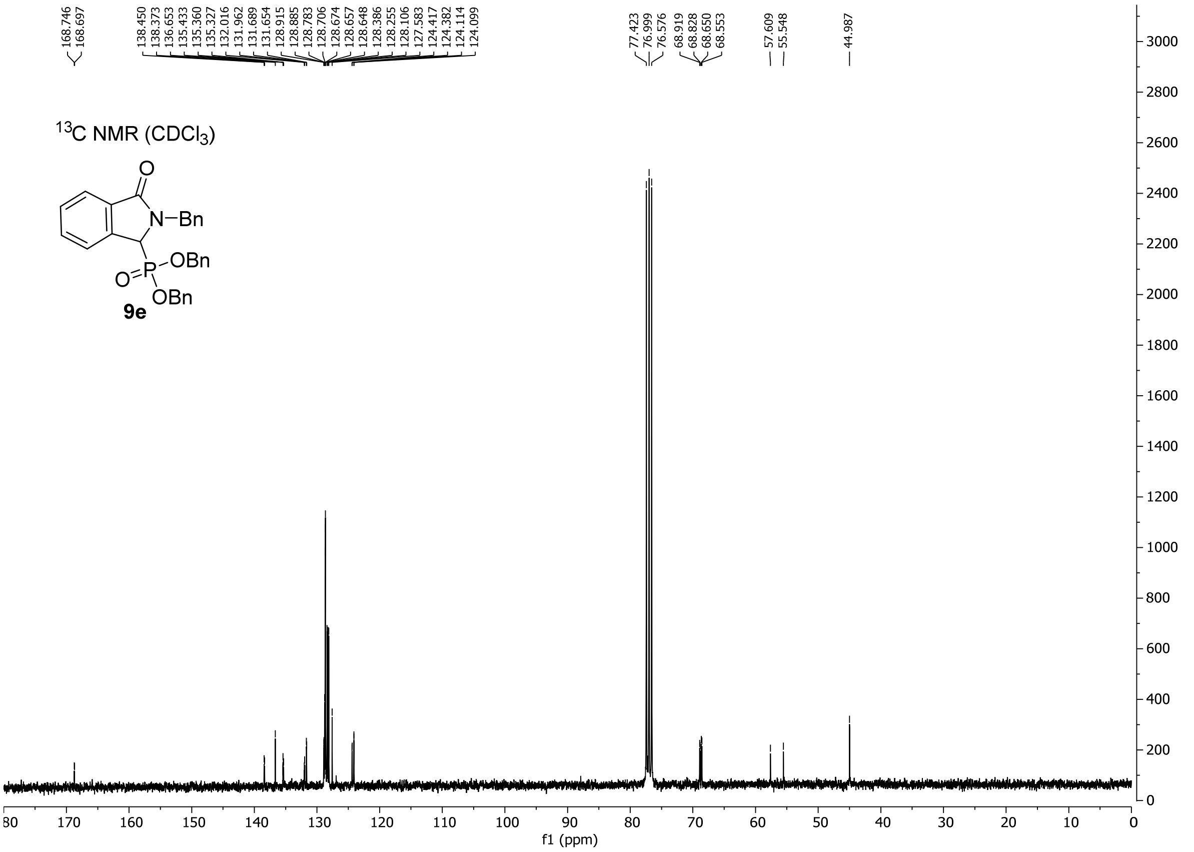

13C NMR (CDCl3)
9e
N−Bn
OBn
OBn
168.746
168.697
138.450
138.373
136.653
135.433
135.360
135.327
132.016
131.962
131.689
131.654
128.915
128.885
128.783
128.706
128.674
128.657
128.648
128.386
128.255
128.106
127.583
124.417
124.382
124.114
124.099
77.423
76.999
76.576
68.919
68.828
68.650
68.553
57.609
55.548
44.987
f1 (ppm)

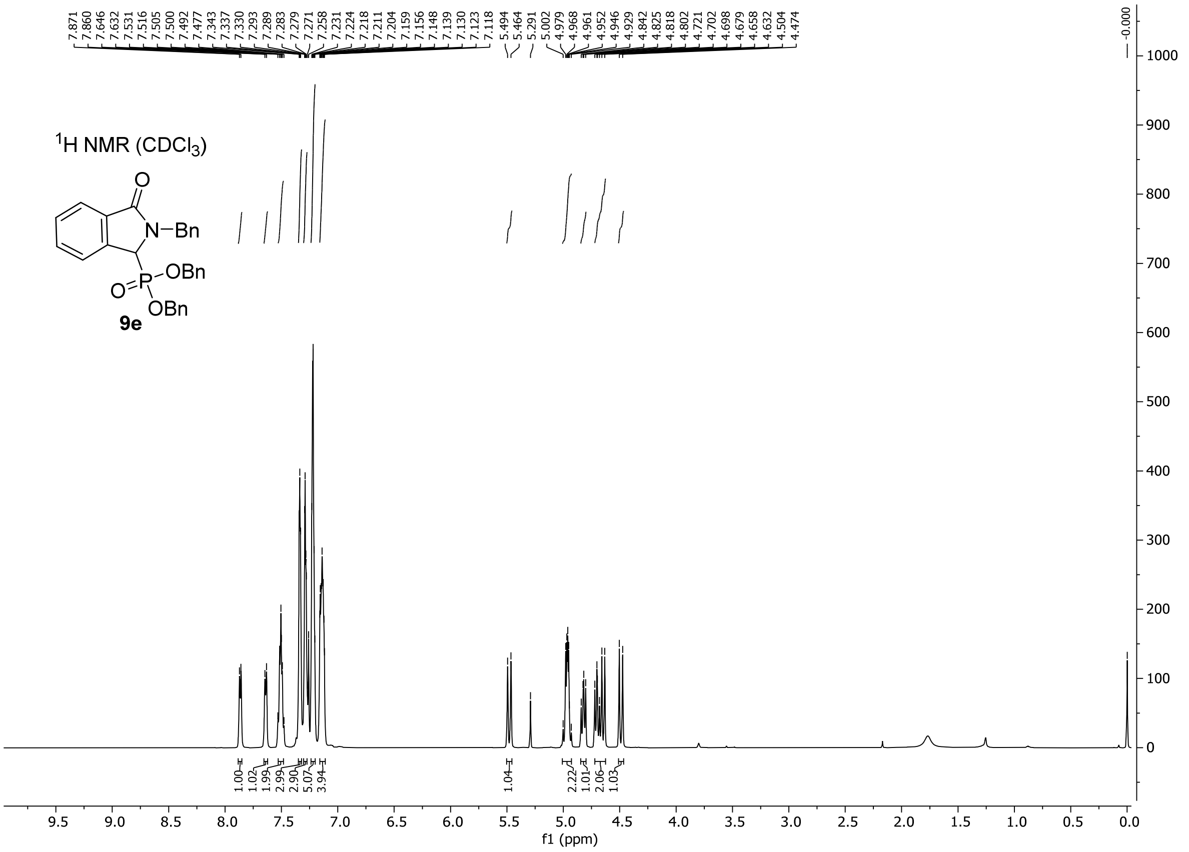
^{1}H NMR ($CDCl_3$)
N−Bn
O
OBn
OBn
9e
7.871 7.860 7.646 7.632 7.531 7.516 7.505 7.500 7.492 7.477 7.343 7.337 7.330 7.293 7.289 7.283 7.279 7.271 7.258 7.231 7.224 7.218 7.211 7.204 7.159 7.156 7.148 7.139 7.130 7.123 7.118
5.494 5.464 5.291 5.002 4.979 4.968 4.961 4.952 4.946 4.929 4.842 4.825 4.818 4.802 4.721 4.702 4.698 4.679 4.658 4.632 4.504 4.474
-0.000
1.00 1.02 1.99 2.99 2.90 5.07 3.94 1.04 2.22 1.01 2.06 1.03
f1 (ppm)

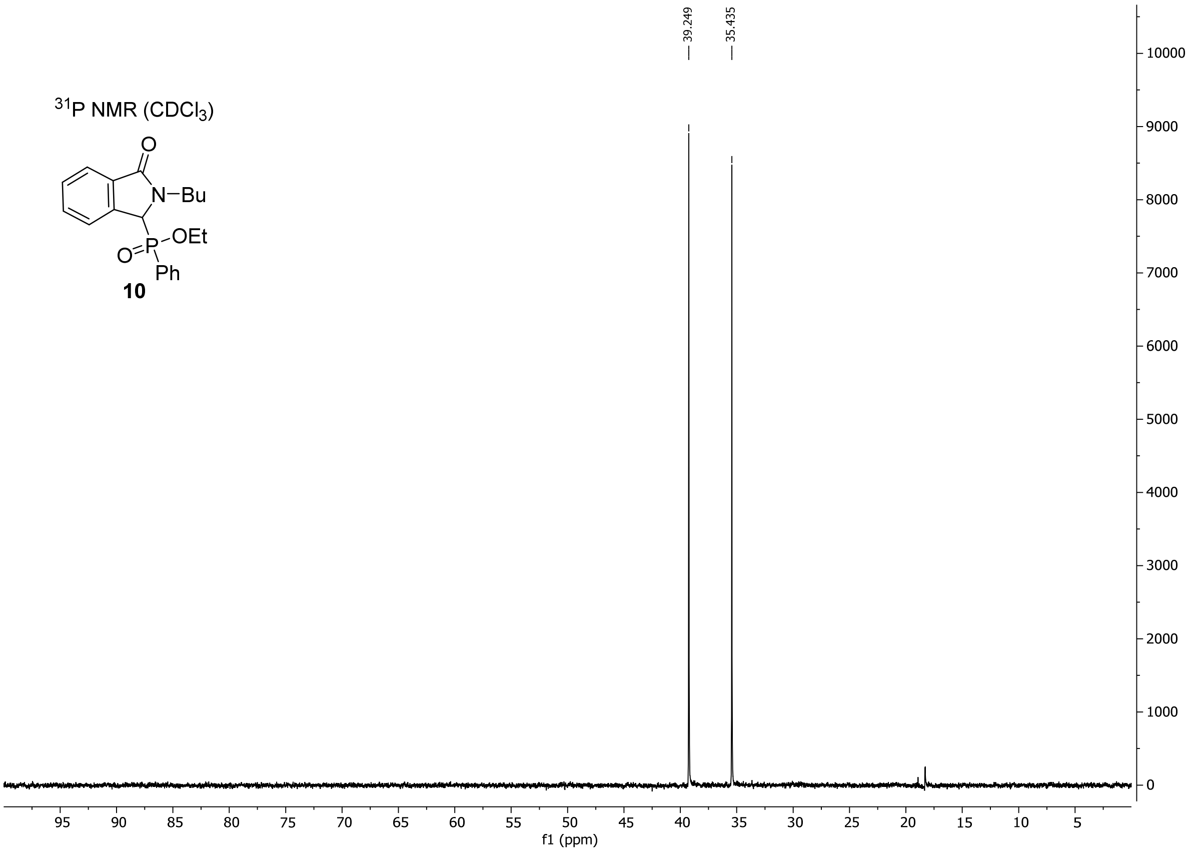
^{31}P NMR ($CDCl_3$)
N−Bu
O
OEt
Ph
10
39.249
35.435
f1 (ppm)

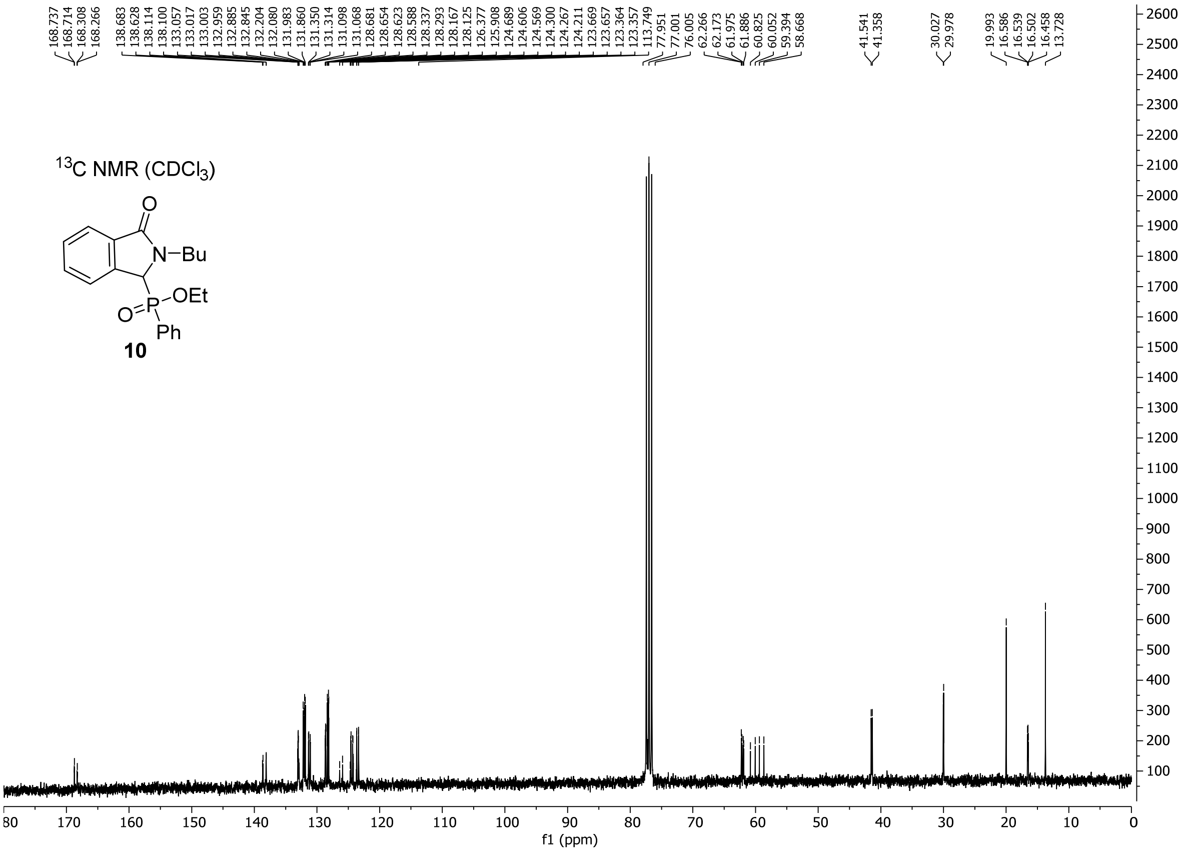
^{13}C NMR (CDCl$_3$)
N−Bu
OEt
Ph
10
f1 (ppm)

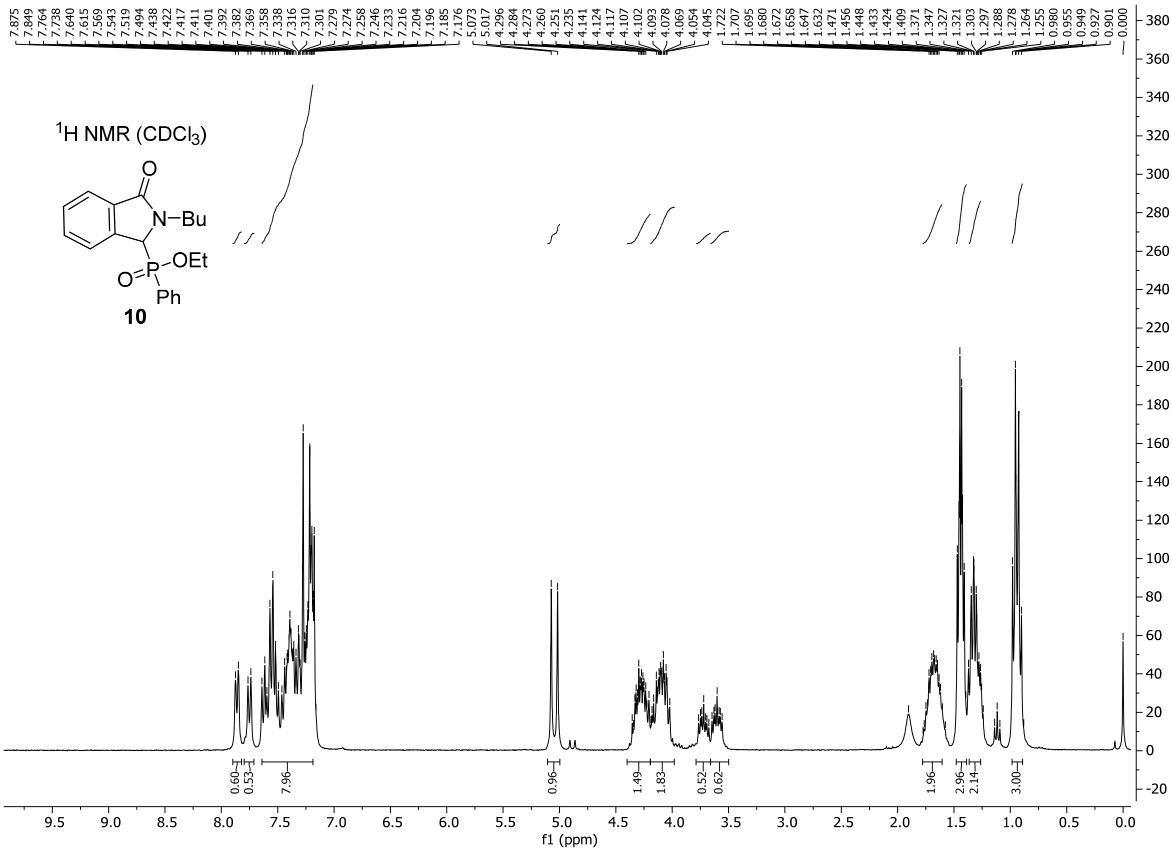
^{1}H NMR (CDCl$_3$)
N−Bu
OEt
Ph
10
f1 (ppm)
